# Fluoride-Cleavable, Fluorescently Labelled Reversible Terminators: Synthesis and Use in Primer Extension

**DOI:** 10.1002/chem.201001952

**Published:** 2011-02-03

**Authors:** Diana C Knapp, Saulius Serva, Jennifer D'Onofrio, Angelika Keller, Arvydas Lubys, Ants Kurg, Maido Remm, Joachim W Engels

**Affiliations:** 1 Institut für Organische Chemie und Chemische Biologie, J.W. Goethe Universität Frankfurt am MainMax-von-Laue Strasse 7, 60438 Frankfurt am Main (Germany), Fax: (+49) 6979829148; 2 Thermo Fisher Scientific (formerly Fermentas)V. Graiciuno 8, 02241 Vilnius (Lithuania); 3 Asper Biotech LtdVaksali 17A, 50410 Tartu (Estonia); 4 Institute of Molecular and Cell Biology, University of TartuRiia 23, 51010 Tartu (Estonia)

**Keywords:** fluorescence, fluoride ions, nucleotides, reverse transcriptase, reversible terminators

## Abstract

Fluorescent 2′-deoxynucleotides containing a protecting group at the 3′-*O*-position are reversible terminators that enable array-based DNA sequencing-by-synthesis (SBS) approaches. Herein, we describe the synthesis and full characterisation of four reversible terminators bearing a 3′-blocking moiety and a linker-dye system that is removable under the same fluoride-based treatment. Each nucleotide analogue has a different fluorophore attached to the base through a fluoride-cleavable linker and a 2-cyanoethyl moiety as the 3′-blocking group, which can be removed by using a fluoride treatment as well. Furthermore, we identified a DNA polymerase, namely, RevertAid M-MuLV reverse transcriptase, which can incorporate the four modified reversible terminators. The synthesised nucleotides and the optimised DNA polymerase were used on CodeLink slides spotted with hairpin oligonucleotides to demonstrate their potential in a cyclic reversible terminating approach.

## Introduction

The completion of the two Human Genome Projects in 2001 implemented the ultimate goal of sequencing the whole human genome.[1, 2] This milestone not only provided a reference genome, but also unexpected opportunities, such as new questions, goals, and hopes, increased demands for improvements in the cost efficiency and throughput of DNA sequencing to an astonishing extent. These requirements not only address sequencing of whole novel genomes, but also the search for individual variation within the human genome. In fact, the latter is a very important issue of the postgenome era, as it promises to elucidate how genetic variation interacts with the environment to confer individual resistance or susceptibility to disease, success of medical interventions, and drug response. For this reason, there has been a rapid development in genotyping technology. For example, many systems for detecting mutations or single nucleotide polymorphisms (SNPs) on a large-scale are currently commercially available.

One genotyping technology, arrayed primer extension (APEX), is a minisequencing microarray assay[3] capable of detecting different types of genetic variations, while combining the efficiency of microarrays (an alternative to gel-based methods) and Sanger sequencing technology.[4, 5] In general, this method can be viewed as DNA sequencing by termination with the use of labelled dideoxynucleotides in a DNA polymerase reaction. However, there is one important difference between APEX and conventional Sanger sequencing. Instead of using one primer and analysing hundreds of extension products with polyacrylamide gel electrophoresis (PAGE), hundreds to thousands of primers are spatially separated beforehand as a two-dimensional array of oligonucleotides. These primers are immobilised by attaching the 5′-end to a glass surface, and each oligonucleotide is extended at the 3′-end by only one dye-labelled dideoxynucleotide complementary to the nucleotide at the variable site. As a result, each primer identifies one base in the target sequence. The advantages of APEX include parallel analysis of hundreds to thousands of genetic variations in a single reaction, high allelic discrimination by the use of a DNA polymerase and four labelled terminators, and the possibility of locus- or disease-specific array design. Therefore, all four possible sequence variants can be detected simultaneously in one reaction. One disadvantage is that one primer is necessary for each position to be identified. This approach can lead to a very high number of oligonucleotides on the array, which can cause problems in fluorescence detection. Depending on the region of DNA that has to be sequenced, it could even exceed the scope of the array.

We have designed and synthesised a new generation of fluorescently labelled, reversibly terminating nucleotides, identified a DNA polymerase that accepts these nucleotides, and optimised the reaction conditions under which they are incorporated into the DNA fragments. The use of these reversible terminators has the potential to fulfil the needs of repeated primer extension reactions on APEX DNA arrays. In this approach, one cycle consists of three steps: 1) DNA polymerase-mediated incorporation of the complementary reversible terminator onto the immobilised oligonucleotide primer sequence, 2) detection of the fluorescence signals specific for each of the four bases, and 3) cleavage of the terminating moiety and the reporter molecule to restore the free 3′-OH group and remove fluorescence signals of already incorporated nucleotides. Repetition of this cycle leads to the template sequence. This approach, which uses our reversible terminators together with the DNA polymerase, provides an opportunity to revolutionise the future of APEX technology because the number of oligonucleotide features on the array could be decreased according to the number of consecutive primer extension cycles on the chip surface.

**Scheme 1:**
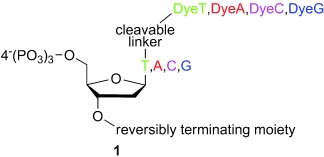
General structure **1** of a fluorescently labelled reversible terminator.

This idea of using 3′-reversibly blocked nucleotides for sequencing was proposed in the beginning of the 1990s. The first examples of potential reversible terminators were reported in 1994 by Metzker and co-workers[6] and Canard and Sarfati.^[7]^ However, the demands these molecules have to meet are challenging, and therefore implementation is difficult. The general structure of a reversible terminator **1** is shown in Scheme 1.

The structural requirements for these nucleotides include a reversibly terminating moiety at the 3′-position and a reporter molecule, such as a dye, attached to the base by a cleavable linker. In the design of a suitable reversible terminator, several important issues have to be considered: Firstly, the 3′-blocking group has to be stable during the polymerase-mediated extension step to ensure effective abortion of elongation after incorporating a single nucleotide. Secondly, a cleavable linker has to be designed to attach the reporter moiety to the base. It is disadvantageous to combine the reporter and blocking group at the 3′-position. Welch and Burgess reported the lack of acceptance of bulky 3′-modifications by DNA polymerases,[8] which was also confirmed by a crystal-structure study of a rat DNA/primer/nucleotide complex.[9] Thus, the linker has to be cleavable under conditions that match the cleavage of the 3′-blocking group to allow both the regeneration of the 3′-OH group and the removal of the linker-dye system in a single deprotection step. Thirdly, the cleavage of the reversibly terminating group and the linker should be quantitative without affecting the DNA-template stability. Fourthly, a polymerase is needed that accepts the 3′-modification and nucleotide modifications and still discriminates strictly between the four bases during the incorporation reaction. Whereas Sanger sequencing has proven that modifications at the 7-position of 7-deazapurines and the 5-position of pyrimidines are well tolerated, the choice of a suitable 3′-modification seems more difficult because the editing properties of polymerases must also be considered.[10] During the last several years, academic and industrial research groups have focused on the design of such reversible terminators. Within the scope of this study, several 3′-blocking groups were investigated, including bulky esters[7] and ethers[8] with the label attached to the blocking group and small groups. Some examples are the 3′-*O*-(2-nitrobenzyl) group investigated by Metzker and co-workers[6] and Welch and Burgess,[8, 11] the 3′-*O*-allyl group reported by Metzker,[6] Ju,[12] and Kim,[13] or the 3′-*O*-azidomethyl group, which was used by Ju and co-workers[14, 15] and was also realised in a commercially available device, the Genome Analyzer developed by Illumina/Solexa.[16, 17] Other interesting groups are the 3′-*O*-NH_2_ group from Benner and co-workers,[18] the 3′-*O*-(2-cyanoethoxy)methyl group reported by us,[19] or some 3′-blocking groups removable under mild reducing or mild acidic conditions reported by Kwiatkowski.[20] The terminators with bulky 3′-modifications exhibited problems with polymerase acceptance. Ester and carbonate linkages are easily cleaved by polymerases, thus leading to multiple incorporation events. Some cases in which the reporter is attached to the base by a cleavable linker, the cleavage conditions of the linker differ from those of the 3′-blocking group. Two different kinds of chemical treatments make these strategies more time consuming, thus showing that this research is very challenging and encourages further investigation of new candidates for reversibly terminating groups.

Herein, we present the synthesis of a complete set of four reversible terminators that bear the fluoride-cleavable 3′-*O*-(2-cyanoethyl) group as a 3′-OH blocking moiety[21] and a suitable fluoride-cleavable linker[22] to connect the nucleoside to a fluorescent dye. The polymerase incorporation experiments and first applications of the reversible terminators in an APEX system are also presented.

## Results and Discussion

**Scheme 2:**
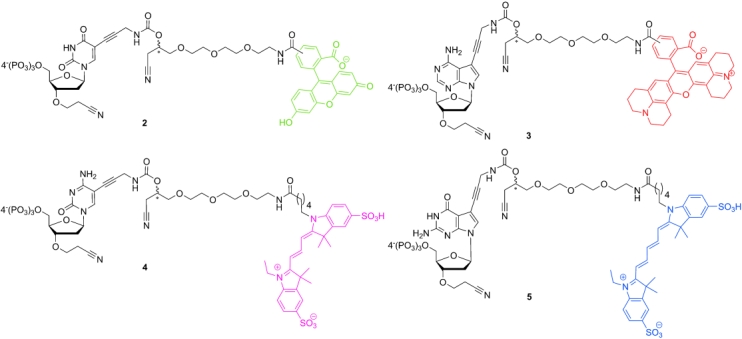
Structures of the fluorescently labelled reversible terminators **2**–**5**.

**Synthesis of the four dye-labelled reversible terminators**: Our target molecules are modified at the 3′-position with the fluoride-cleavable 2-cyanoethyl group[21] and at the base with our recently reported fluoride-cleavable linker (Scheme 2).[22] The four fluorescent dyes chosen for labelling the four reversible terminators are 5- and 6-carboxyfluorescein for thymidine, cyanine 3.0 (Cy 3.0) for 2′-deoxycytidine, 5- and 6-carboxy-X-rhodamine for 2′-deoxyadenosine, and cyanine 5.0 (Cy 5.0) for 2′-deoxyguanosine. These dyes were chosen as they are spectrally well separated from each other, well known, and commercially available. In addition, the same fluorescent dyes have been already used as dideoxy terminator conjugates for a couple of years in classical APEX reactions on a Genorama platform for SNP genotyping,[23] mutation detection,[24] and APEX-based resequencing.[25]

**Scheme 3:**
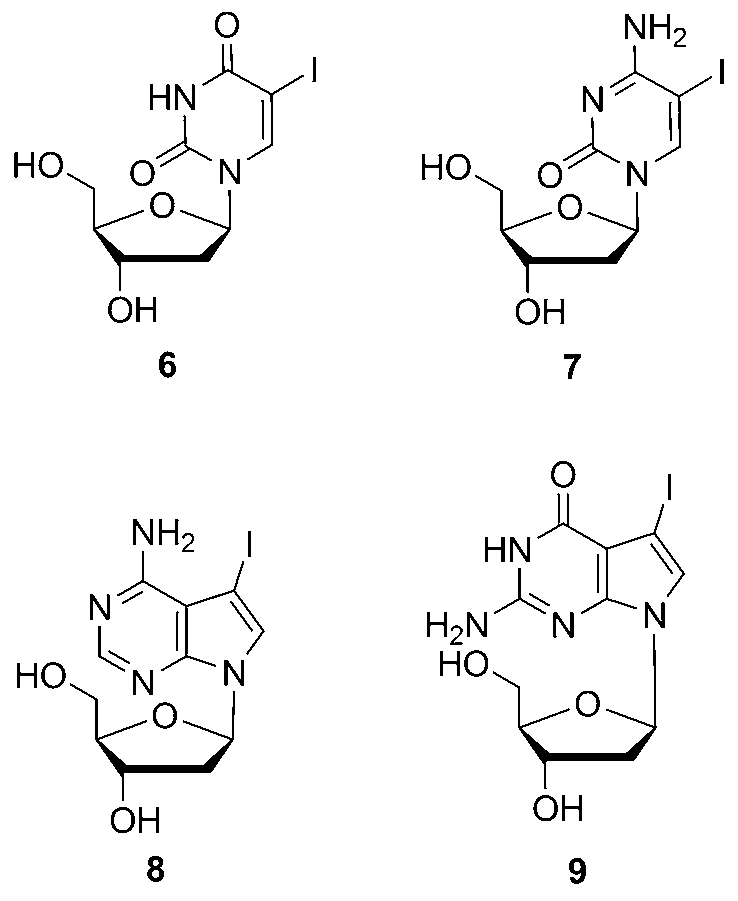
The four starting compounds **6**–**9**.

To synthesise the four reversible terminators, there are two crucial steps: 1) introduction of the 3′-OH modification and 2) introduction of the linker-dye system. The linker was incorporated by the well-known Sonogashira cross-coupling reaction.[26, 27] As a prerequisite for this method of attachment, the four iodonucleosides (i.e., 5-iodo-2′-deoxyuridine (**6**), 5-iodo-2′-deoxycytidine (**7**), 7-deaza-7-iodo-2′-deoxyadenosine (**8**), and 7-daza-7-iodo-2′-deoxyguanosine (**9**; shown in Scheme 3) are required starting compounds. Compounds **6** and **9** were purchased from commercial sources, whereas **7** and **8** were synthesised in our laboratory by combining steps from reported procedures that are not described in detail herein.[28–32]

**Scheme 4:**
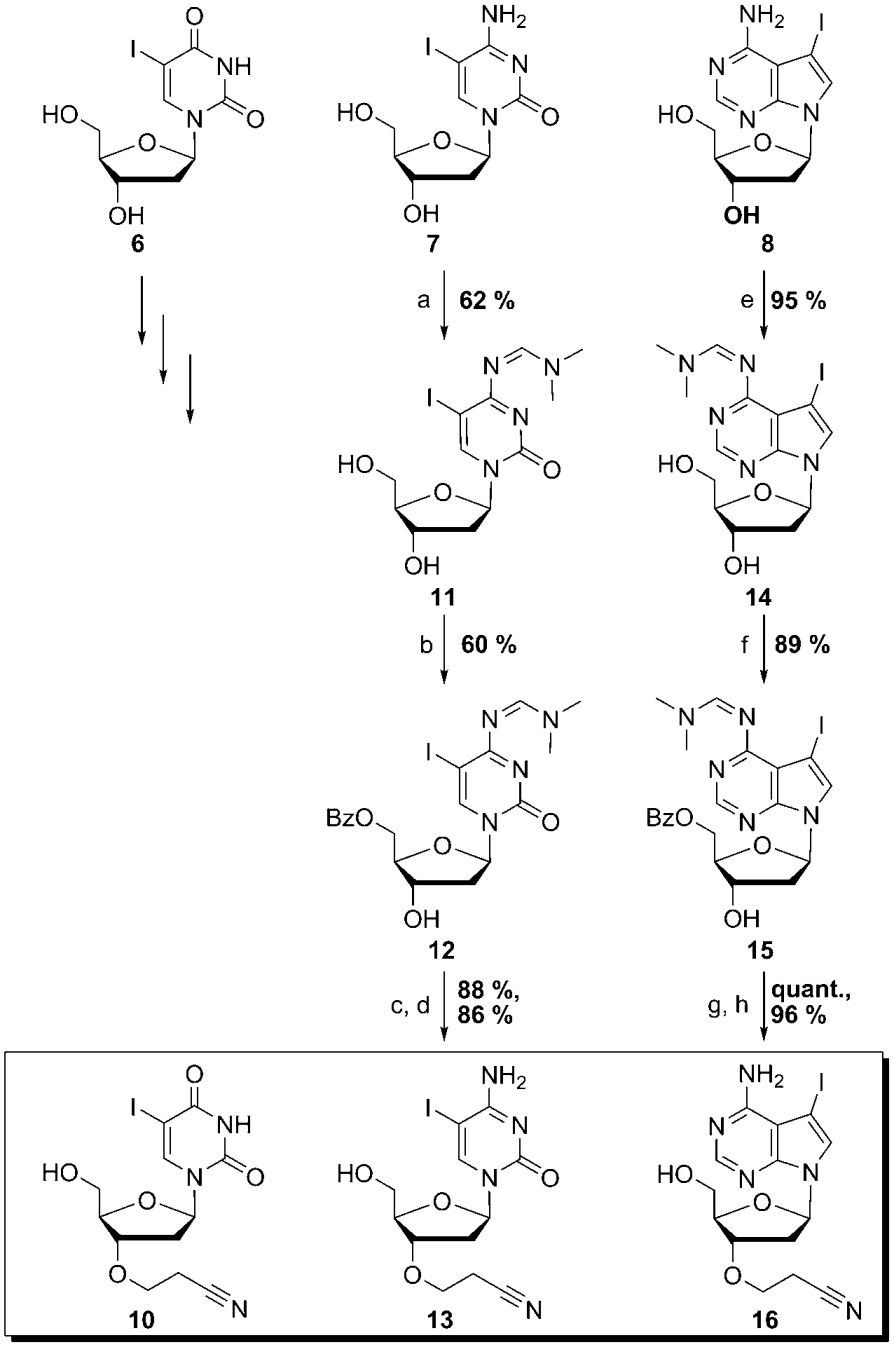
Protecting-group strategy and introduction of the 3′-modification into **6**,[22] **7**, and **8**. Reagents and conditions: a) *N*,*N*-dimethylformamide dimethylacetal, dry DMF, 55 °C, 2.5 h; b) BzCl, dry pyridine/dry DMF=4:1, 0 °C→rt, 2 h; c) acrylonitrile, Cs_2_CO_3_, *t*BuOH/dry DMF 2:1, rt, 3 h; d) saturated methanolic ammonia, rt, 2.5 h; e) *N*,*N*-dimethylformamide dimethyl acetal, dry DMF, 50 °C, 2 h; f) BzCl, dry CH_2_Cl_2_, dry pyridine, −15 °C, 1 h; g) acrylonitrile, Cs_2_CO_3_, *t*BuOH, rt, 2 h; h) saturated methanolic ammonia, 50 °C, 2 h. Bz=benzoyl, BzCl=benzoyl chloride.

**Scheme 5:**
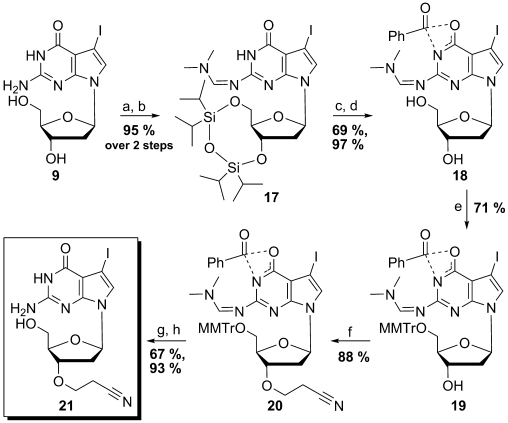
Protecting-group strategy and introduction of the 3′-modification into **9**. Reagents and conditions: a) 1,1,3,3-tetraisopropyldichlorodisiloxane, dry pyridine, 0 °C→rt, 1 h; b) *N*,*N*-dimethylformamide dimethylacetal, dry DMF, rt, 24 h; c) BzCl, dry pyridine, dry CH_2_Cl_2_, 0 °C→rt, 2 h; d) Et_3_N⋅3 HF, THF, rt, 1 h; e) MMTrCl, DMAP, dry pyridine, rt, 18 h; f) acrylonitrile, Cs_2_CO_3_, *t*BuOH, rt, 2 h; g) PTSA, CH_2_Cl_2_/EtOH 1:1, rt, 1 h; h) 32 % aqueous NH_3_, MeOH, rt, 18 h. DMAP=*N*,*N*-dimethylaminopyridine, MMTr=monomethoxytrityl, PTSA=*para*-toluenesulfonic acid.

For selective introduction of the 2-cyanoethyl group at the 3′-position, it is necessary to protect all the other functional groups. Saneyoshi et al. described the protection of the 2′-OH group with the 2-cyanoethyl group of all four RNA nucleosides with acrylonitrile in a Michael addition reaction with Cs_2_CO_3_ as a heterogeneous base in *t*BuOH.[33] For DNA, the modification of the 3′-OH group with the 2-cyanoethyl group has been reported by us for thymidine and **6**.[21, 22] For the other three nucleosides **7**–**9**, we developed protecting-group strategies to enable the selective introduction of the 3′-(2-cyanoethyl) group (Schemes 4 and 5).

We used base-labile protecting groups for the amino functions and 5′-OH group. The synthesis of the 3′-*O*-(2-cyanoethyl)-modified 2′-deoxyuridine analogue **10** was accomplished, as recently reported by us.[22] The same strategy was used for the protection of the functional groups of **7** and **8**. The exocyclic amino groups of both nucleosides were protected with formamidine. No acidic proton should be left to prevent the reaction of its conjugated base in the Michael addition. Both reactions were carried out following a procedure described for 5-iodo-2′-deoxycytidine.[34] The formamidino-protected 2′-deoxycytidine analogue **11** was obtained in a moderate yield of 62 %. Whereas the previously reported yield[35] for the 2′-deoxyadenosine analogue **14** could be enhanced from 85 to 95 %. By carrying out the 5′-selective benzoylation of **11** at 0 °C at room temperature, **12** was produced in a moderate yield of 60 %. The 2′-deoxyadenosine analogue **15** could be obtained in excellent yield by carrying out the reaction at a low temperature (−15 °C). The Michael addition was accomplished by following procedures reported by Saneyoshi et al.[33] and by our group.[21, 22] Freshly distilled acrylonitrile was used in *t*BuOH as the solvent and Cs_2_CO_3_ as the base. One modification of the procedure was necessary for **12**; in this case, DMF was used as a cosolvent to enhance the solubility of the starting material. After purification, the fully protected intermediate was isolated in 88 % yield. The fully protected 3′-modified 7-deaza-7-iodo-2′-deoxyadenosine analogue was obtained in a quantitative yield. The deprotection reactions that lead to compounds **13** and **16** were carried out in saturated methanolic ammonia to result in yields of 86 and 96 % of **13** and **16**, respectively. The first three 3′-modified key compounds, namely, **10**, **13** and **16**, could be obtained by using very similar protecting-group strategies, which effectively enabled the selective modification of the 3′-OH group. Another protecting group strategy had to be applied for the fourth nucleoside **9** because guanosine analogues have an additional functional group that has to be protected. The developed synthetic procedure was optimised first by using natural 2′-deoxyguanosine (not shown) and then transferred to the 7-deaza-7-iodo derivative **9**. The synthesis of the 3′-*O*-(2-cyanoethyl) modified derivative **21** is shown in Scheme 5.

For the protection of the N^1^ atom, we chose benzoyl as a blocking group during the synthesis of the 2′-deoxyguanosine analogue **21**. For the intermediate protection of the 3′- and 5′-OH groups, we used the Markiewicz procedure.[36] For the protection of the exocyclic amino group, the formamidino group was used. These two steps were carried out without an intermediate purification step, thus furnishing **17** in 95 %. The subsequent benzoylation of the N^1^ atom was carried out between 0 °C and room temperature and proceeded in 69 % yield. When followed by deprotection of the 5′- and 3′-OH groups with Et_3_N⋅3 HF in THF, the amino-protected compound **18** could be isolated in 97 % yield. During the synthesis of the 2′-deoxyguanosine analogue **21**, we deviated from the exclusively base-labile protecting-group strategy. To improve the solubility properties for the Michael addition reaction, we used the MMTr group to protect the 5′-OH group of **18** to obtain fully-protected **19** in 71 % yield. The 3′-modification was accomplished by following the same procedure employed for the 2′-deoxyuridine analogue and the 2′-deoxyadenosine analogue **15**, thus providing fully protected **20** in an excellent yield of 88 %. After deprotection, first with PTSA for removal of the MMTr group followed by aqueous ammonia for cleavage of the amino protecting groups, the fourth key compound **21** could be obtained in 62 % yield over the last two steps.

**Scheme 6:**
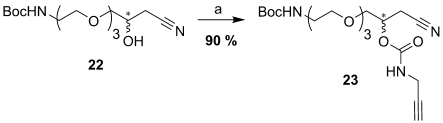
Synthesis of the linker derivative **23**. Reagents and conditions: a) 1. DSC, anhydrous K_2_CO_3_, dry CH_3_CN, 0 °C, 20 h; 2. propargylamine, KHCO_3_, 0 °C →rt, 5 h. Boc=*tert*-butoxycarbonyl, DSC=*N*,*N′*-hydroxysuccinimidyl carbonate.

Recently, we described the synthesis of the 5- and 3′-modified 2′-deoxyuridine analogue **24**.[22] We coupled 5-iodonucleoside **10** to 1,1,1-trifluoroacetyl-protected propargylamine and, after subsequent deprotection, attached the linker to the free amino group through the *N*-hydroxysuccinimidyl carbonate group. Therein, we describe an improvement of this procedure by synthesising the linker derivative **23** in a one-pot procedure (Scheme 6).

**Scheme 7:**
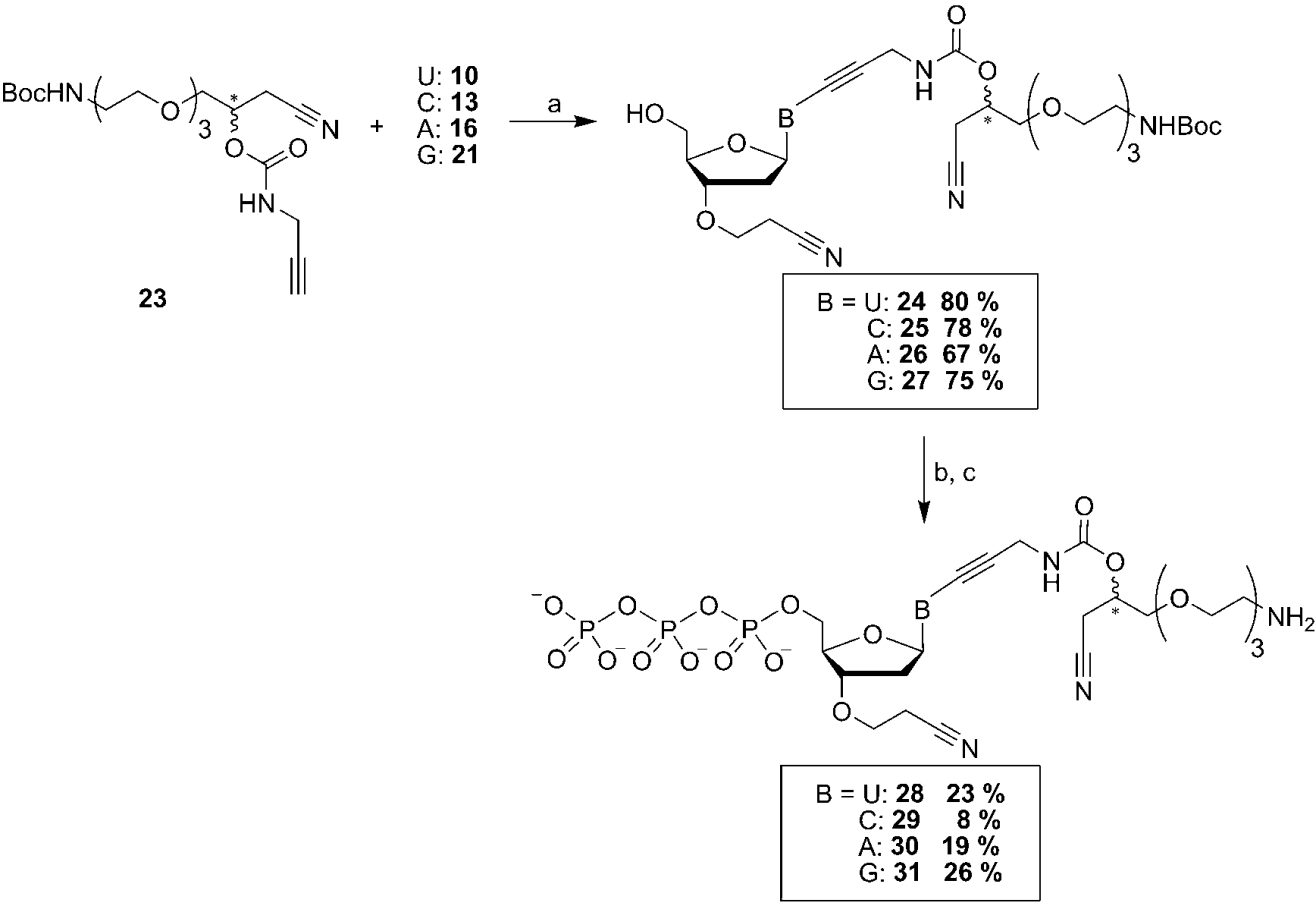
Linker attachment and triphosphate synthesis with all four 3′-O-(2-cyanoethyl) modified nucleosides **10**, **13**, **16**, and **21**. Reagents and conditions: a) dry DMF, triethylamine, CuI (0.2 equiv), [Pd(PPh_3_)_4_] (0.1 equiv), alkyne **23** (1.2–2.0 equiv), rt, 3.5–5 h; b) 1. dry pyridine/dry dioxane (1:30, 2-chloro-4*H*-1,2,3-benzodioxaphosphorin-4-one, rt, 15 min; 2. dry DMF, bis(tributylammonium)pyrophosphate, tributylamine, rt, 20 min; 2. iodine in pyridine/water (2 %, 98:2), rt, 20 min; 4. 5 % Na_2_SO_3_ solution; c) TFA (40 equiv) in water, rt, 5–6 h. TFA=trifluoroacetic acid.

Compound **23** enables a direct Sonogashira coupling to the 3′-modified iodonucleosides **10**, **13**, **16** and **21** (Scheme 7). This route saves two synthetic steps, and the yield of **24** could be improved from 49 % over three steps to 80 % in one step.

**Figure 1:**
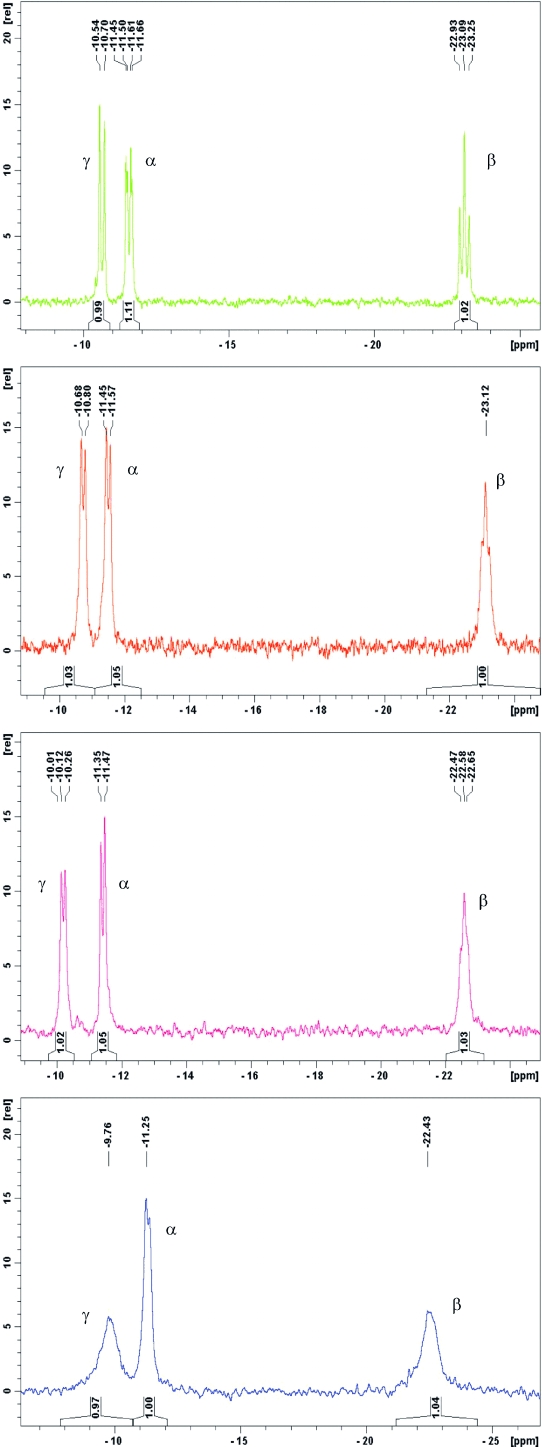
^31^P NMR spectra of the four dye-labelled reversible terminators **2**–**5**.

The Sonogashira coupling was applied to all four nucleosides **10**, **13**, **16** and **21** under the same conditions (i.e., a Pd^0^/Cu^I^ catalyst system, DMF, Et_3_N, rt). The desired derivatives were obtained in good-to-very-good yields and with very high purities. The triphosphate synthesis was accomplished by following the procedure of Ludwig and Eckstein reported in 1989.[37] In this step, 2-chloro-4*H*-1,2,3-benzodioxaphosphorin-4-one is used as the phosphorylating reagent that is subsequently opened with pyrophosphate and finally oxidised with a 2 % iodine solution in pyridine/water. In general, the reaction proceeds well, as demonstrated by analysing the crude reaction product by ^31^P NMR spectroscopic analysis (not shown). These crude products were treated directly with TFA/water to remove the Boc protecting group from the amino function of the linker moiety. The compounds were obtained in yields of around 20 % for **28**, **30** and **31** and only 8 % for **29** due to the multistep purification of these compounds, which is crucial after this step. Thus, two purification steps had to be performed to achieve the necessary purity. Anion-exchange chromatography at 4 °C on a fast-protein liquid chromatography (FPLC) system was the first step followed by reverse phase (RP)-HPLC to yield **28**–**31** with a high degree of purity in quantities between 22 and 120 mg (**31** and **28**, respectively). The obtained triphosphate derivatives produced positive results in the following labelling reactions with the selected fluorescent dyes. All the dyes (i.e., 5- and 6-carboxyfluorescein, 5- and 6-carboxy-X-rhodamine, Cy 3.0, and Cy 5.0) were used in the activated *N*-hydroxysuccinimidyl ester form. The coupling reactions were carried out in dry DMF with KHCO_3_ as a heterogeneous base in the case of **28** and *N*,*N*-diisopropylethylamine (DIPEA) as a base for the other nucleotides **29**–**31**. The purification of the final compound was also very laborious; prepurification on the RP-FPLC system with self-packed columns was necessary to remove the starting-material dye before final purification with RP-HPLC was possible. After this procedure, the four reversible terminators **2**–**5** (Scheme 2) were obtained with a high degree of purity in quantities between 1.5 and 6 mg (for **5** and **2**, respectively) and confirmed by ^1^H and ^31^P NMR spectroscopic and mass-spectrometric analysis. The ^31^P NMR spectra of all four compounds prove the structure of the obtained compounds (Figure 1).

The four labelled reversible terminators **2**–**5** were used in polymerase incorporation assays and in the demonstration of a proof-of-principle for reversible primer extension on immobilised template oligonucleotides.

**Figure 2:**
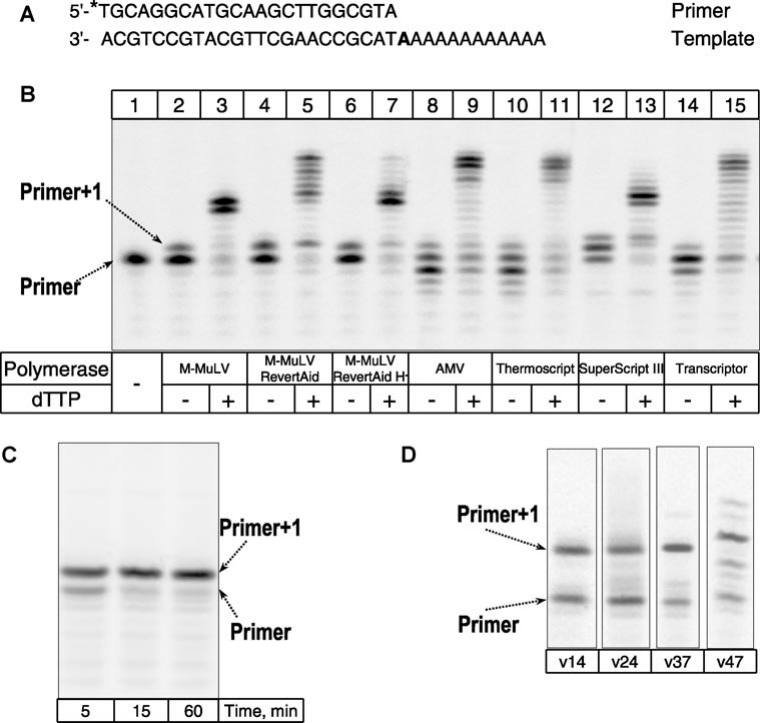
The use of 3′-*O*-(2-cyanoethyl)-dNTP derivatives for primer extension by reverse transcriptases. A) DNA substrate used (* indicates the radiolabelled 5′-terminus). The position in the template strand that directs the nucleotide to be incorporated is in bold type. B) Incorporation of 3′-*O*-(2-cyanoethyl)-dTTP. Lanes 1: starting-material primer, polymerases; 2 and 3: M-MuLV; 4 and 5: RevertAid M-MuLV; 6 and 7: RevertAid H minus M-MuLV; 8 and 9: AMV (cloned); 10 and 11: ThermoScript; 12 and 13: SuperScript III; 14 and 15: Transcriptor. dTTP was added into the reaction mix after the primer extension step, as indicated for lanes 3, 5, 7, 9, 11, 13, and 15. C) Time course of incorporation of 3′-*O*-(2-cyanoethyl)-dTTP by RevertAid M-MuLV reverse transctiptase in the presence of Mn^2+^ ions. D) Extension of the corresponding substrates by the dye-labelled 3′-*O*-(2-cyanoethyl)-modified nucleotides **2**–**5** during a reaction time of 5 min.

**Polymerase identification for primer extension**: Several aspects of the system appear to be critical to incorporate reversible terminators enzymatically into the nucleic acid substrate. The polymerase must have the proper affinity and selectivity towards modified nucleotides along with adequate turnover rate and propensity to form terminated DNA molecules that remain stable until the 3′-modifications are removed by chemical means. In turn, the reversible terminator itself must display an appropriate balance between chemical stability during enzymatic incorporation and the ability to be unblocked under conditions mild enough for the DNA structure to be retained for further enzymatic treatment. A solution-based DNA primer extension system that is suitable for simultaneous evaluation of substrate extension by either single or multiple T residue(s) was used for initial screening of a wide range of polymerases that represent major families of DNA polymerases (A, B, X, Y, and reverse transcriptases) and some of their mutants. This system enables the gel-based detection of primer extension products with single nucleotide resolution. By following the primer-extension step, natural thymine triphosphate (dTTP) was added to check the stability of the termination of the resulting +1 product. Polymerases that exhibited even minimal proofreading activity were found to be incompatible with the assay due to primer truncation (data not shown). We found 3′-*O*-(2-cyanoethyl)-dTTP[21] to be incorporated into DNA (Figure 2 A) by several reverse transcriptases only (Figure 2 B). All enzymes possess the ability to incorporate the compound (see even-numbered lanes for bands that appear just above the primer), although the efficiency of incorporation varies. RevertAid M-MuLV reverse transcriptase yielded a single primer-extension product (Figure 2 B, lane 4) that was resistant towards extension by supplemented dTTP (Figure 2 B, lane 5) due to its 3′-terminal modification. Optimisation of the reaction conditions was necessary due to the low efficiency of the +1 extension (reaction time was 60 min) and was accomplished by including 1 mm MnCl_2_ into the reaction mixture. The reaction rate increased significantly, thus driving the primer-extension reaction to completion in just 15 min (Figure 2 C). Incorporation of the dye-conjugated 3′-modified compounds **2**–**5** under identical conditions is of comparable speed. Figure 2 D presents the profiles of the primer extension reactions that involve compounds **2**–**5** after 5 min.

**Figure 3:**
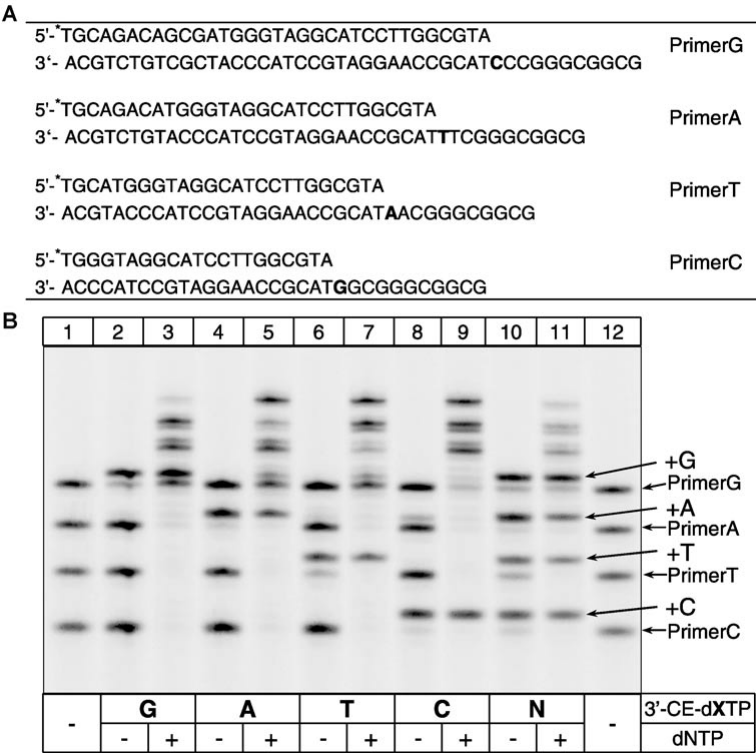
Specific primer extension with 3′-*O*-(2-cyanoethyl)-dNTP derivatives by RevertAid M-MuLV reverse transctiptase. A) Set of DNA substrates used for specificity studies (* indicates radiolabelled 5′-termini). The position in the template strand that directs the nucleotide to be incorporated is in bold type. B) The initial primer-extension step (lanes 2, 4, 6, 8, and 10) was followed by the addition of a dNTP mixture to the solution containing all four natural dNTPs dissolved in water and incubation for 5 min (lanes 3, 5, 7, 9, and 11). Lanes 1 and 12: the starting-material primer set; 2 and 3: 3′-*O*-(2-cyanoethyl)-dGTP; 4 and 5: 3′-*O*-(2-cyanoethyl)-dATP; 6 and 7: 3′-*O*-(2-cyanoethyl)-dTTP; 8 and 9: 3′-*O*-(2-cyanoethyl)-dCTP; 10 and 11: full set of 3′-*O*-(2-cyanoethyl)-dNTP nucleotides.

The specificity of primer extension was addressed by performing the reaction under multiplex conditions, in which four DNA substrates of different length, each competent for extension by a particular nucleotide only (Figure 3 A), were simultaneously present in the reaction mixture. Incorporation of the individual 3′-modified nucleotides (Figure 3 B, lanes 2, 4, 6 and 8) or a mixture of all four (Figure 3 B, lane 10) demonstrated the performance of the system under conditions typical for sequencing reactions. DNA substrates are extended by relevant 3′-modified nucleotides only and remain unaffected in presence of natural 2′-deoxynucleotide triphosphates (Figure 3 B, lanes 3, 5, 7, 9 and 11). At this stage, a more detailed assessment of polymerase specificity was not performed due to differences between the conditions for the liquid-state reactions and the ones involving the microchip surface, typical for APEX.

**Extension of immobilised DNA templates**: The complete system was tested in a reversibly terminating approach by using the set of our four fluorescently labelled reversible terminators **2**–**5**, the RevertAid M-MuLV reverse transcriptase, and the reaction buffer optimised for primer extension.

**Figure 4:**
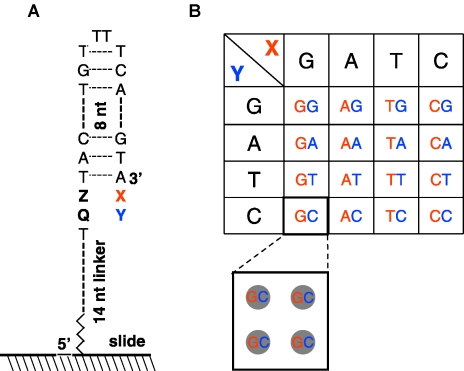
Schematic illustration of the hairpin primers and the spotting layout of the slides. A) The variable positions in the template part are marked as Z for the first template nucleotide and as Q for the second. The variable positions immediately after the 3′-terminus are marked as X and Y for the first and second nucleotide to be incorporated, respectively. B) Each of the 16 oligonucleotides is applied on the slide in four spots arranged on the grid in squares. In a column (vertically), all the four squares encode the same base for the first incorporation event (X). Each square within a row (horizontally) encodes one of the four nucleotides for the second incorporation event (Y).

**Figure 5:**
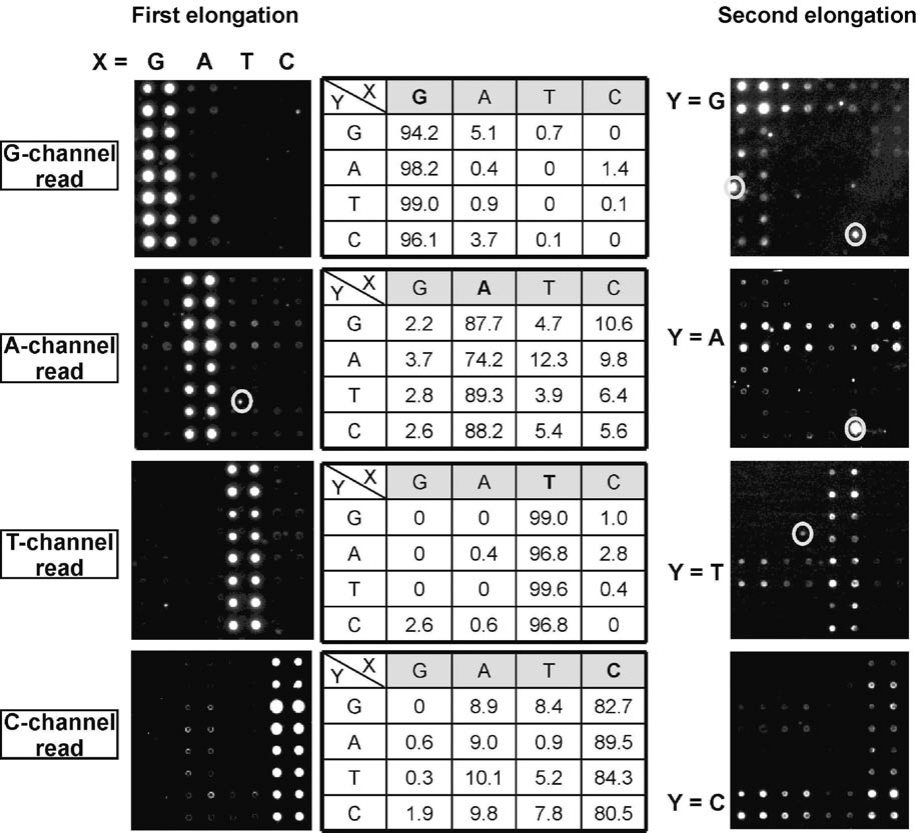
Imaging for illustration and analysis of the primer-extension events. Signals of the first elongation appear vertically (in column), signals of the second elongation appear horizontally (in rows). Evaluation of the specificity of the primer extension is given in the tables, and the target nucleotide is in bold type. Fluorescence readout channels are shaded and present the data in columns (vertically) for the first extension step. Signals in circles are artefacts.

To test the labelled reversible terminators **2**–**5** for their incorporation into immobilised DNA, a set of 16 oligonucleotides that differ at positions Q and Z was designed to provide a readout of all possible variants of two consecutive nucleotides at X and Y positions (Figure 4 A). These primers have an internal self-complementary region that allows folding into a hairpin structure, thus providing a double-stranded 3′-terminus for extension by the polymerase. All the oligonucleotides were functionalised with a 5′-C6 amino modification to enable their immobilisation onto the amino-reactive array surface. We chose CodeLink-activated slides because their glass surface is coated by a synthetic material that is compatible with our deprotection conditions (fluoride ions) in contrast to glass slides that would be harmed by a prolonged fluoride treatment (data not shown). The spotting was accomplished by using a 10 μm solution of each oligonucleotide in 100 mm carbonate buffer (pH 9.0) with the Versarray Chipwriter Pro Arrayer. The spotting layout of the CodeLink slides is shown in Figure 4 B and is arranged so that the first primer-extension event is conveniently followed by signals that appear vertically (Figure 4 B, red letters) and the second event represented by signals that appear horizontally (blue letters). The primer layout shown in Figure 4 was spotted twice on each slide (array). The composition of the reaction mixture for the extension of immobilised primer was further improved by the addition of bovine serum albumin (BSA), a reported compound to counteract protein adsorption and inactivation on wafer surfaces.[38] Furthermore, numerous osmolytes were screened and 2-hydroxymethyl[18]crown-6 was determined to be the most efficient enhancer of primer extension. Evaluation of spotted CodeLink slides for the acceptance of all four fluorescently labelled reversible terminators **2**–**5** confirmed the time-dependent character of the primer extension. The maximum signal was obtained after a reaction time of 30 min (not shown). This time-point was used in the reversible primer-extension experiment (Figure 5).

Figure 5 depicts the results of the fluorescence detection after the first and second elongation steps in which each picture represents a scan of one channel. The tables in the middle of Figure 5 show the specificity values of the first elongation step estimated from the two arrays spotted on the slide. The specificity of a particular fluorescent-nucleotide incorporation into the first position X of the primers was calculated by combining the signals from four primers located within the same row and that bear the same nucleotide at the Q position (Figure 4 A) and by evaluating the percentile of detected fluorescence within each of the four primers. This approach enables the evaluation of the polymerase specificity within the same sequence context. The imaging results obtained after the first step showed that all four reversible terminators were incorporated with high selectivity (as summarised in Figure 5). We calculated that the specificity of the incorporation of **5** (channel read G) into primer GG reached 94.2 % and the highest nonspecific incorporation of **5** is 5.1 % of the total signal into primer AG. Incorporation of **2** (channel read T) was the most specific and varied from 96.8 to 99.6 %; lower specificity was observed for **3** and **4** (channel read A and C; 74.2–89.3 and 80.5–89.5 %, respectively).

Preliminary experiments revealed that a fraction of the spotted oligonucleotides remains unextended by reverse transcriptase during the first elongation step (data not shown), thus resulting in the appearance of a high background during the second elongation step. The terminal deoxynucleotide transferase (TdT) catalyses a template-independent addition of 2′-deoxyribonucleotides to the 3′-OH group of various DNA and RNA substrates and efficiently incorporates the polymerisation terminator 2′,3′-didesoxyadenosine-5′-triphosphate (ddATP). On the other hand, we found that TdT does not extend DNA substrates that bear the 3′-*O*-(2-cyanoethyl) protecting group (results not shown). Therefore, the first elongation step was followed by incubation of the spotted slides with TdT in the presence of ddATP. During this treatment, it was expected that unlabelled (and uncleavable) 3′-dideoxy terminators would be incorporated into the structure of previously unextended primers and prevent them from being extended during the second elongation step. Afterwards, the slide was imaged and treated with a 1:1 solution of tetrabutylammonium fluoride (TBAF; 1 m)/THF and DMF at 45 °C for 15 min to cleave the fluorescent dye and 3′-*O*-(2-cyanoethyl) group. To ensure that the linker-dye moiety was completely removed, this process was controlled by a separate imaging step in which only the background-level fluorescence signal could be detected (not shown).

On these deprotected slides, the second round of primer extension was executed under conditions identical to the first round. The pictures of fluorescence detection after the second elongation are displayed in Figure 5 as well. During this step, the same nucleotide is expected to be incorporated into primers positioned in the same row (horizontally). These pictures demonstrate the feasibility of the second primer extension because the spots are detected at expected positions. However, the efficiency of the incorporation of the individual fluorescently labelled reversible terminators **2**–**5** was 15–30 times lower relative to that observed during the first elongation step. The selectivity suffered mainly from incorporation into those primers that were substrates during the first elongation step. The latter phenomenon is manifested by the appearance of additional signals in columns (images on the right part of Figure 5) the positions of which coincide with those labelled during the first elongation step (images on the left part of Figure 5). Several factors might account for this discrepancy during the primer extension on immobilised templates. During the experiments on the slides, the efficiency of the unblocking process was monitored by measuring the remnant fluorescence and expecting the 3′-*O*-(2-cyanoethyl) group to be removed as efficiently as the linker-dye system. This outcome was true for chemical unblocking in solution or on a controlled-pore glass (CPG)-immobilised oligonucleotide,[22] but not necessarily for hairpin-forming oligonucleotides immobilised on a slide. Therefore, a probable reason for the decreased incorporation efficiency during the second elongation step could be the nonquantitative chemical deprotection of the 3′-OH group. On the other hand, we observed that the enzyme we used extends a fraction rather than all spotted oligonucleotides, and this feature should also be responsible for the partial decrease in the signal intensity after the second elongation step. The apparently less-selective incorporation of modified nucleotides **2**–**5** during the second elongation step appears to be related to a fraction of substrates that remain unblocked during the first elongation step and subsequent TdT treatment (Figure 5, compare the images on the left and right). However, this finding is true for the incorporation of all the modified nucleotides, except for compound **3** (Figure 5; see the channel read A) in which no increased fluorescence was observed after the second elongation step in column A. On the basis of this observation, it might be speculated that ddATP incorporation by TdT was the most complete when the complementary T nucleobase was present in the template strand (all four primers of such structure are located within the A column). On the other hand, unextended primers might become available not only if treatment by TdT is incomplete, but also as a result of pyrophosphorolytic dismutation.[39] One also should consider that all four nucleotides employ a linker between the nucleobase and fluorescent dye, which results in a propargylamine residue after the cleavage of the dye. The modification of the nucleobase is an additional stimulus for triphosphate-maintained phosphorolysis,[40] thus resulting in truncated primers available for further extension.

Altogether, we have demonstrated the feasibility of the template-directed incorporation of the 3′-terminated dye-labelled nucleotides **2**–**5** that can be cleaved in one step to enable the next addition, thus providing the proof-of-principle of a functional system also targeted to run in cyclic mode.

## Conclusion

Herein, we have described the synthesis and full characterisation of four fluorescently labelled reversible terminators **2**–**5**. In these molecules, the 3′-blocking moiety and the linker-dye system are removable during the same fluoride-based deprotection treatment. The synthesised molecules are recognised and accepted by an identified DNA polymerase. The reversible terminators and identified polymerase, which was able to incorporate them, were also used in a cyclic reversibly terminating approach on CodeLink slides spotted with hairpin oligonucleotide probes. These results demonstrate that the entire system is applicable in a cyclic reversibly terminating approach. Further studies that address the specificity and efficiency of the complete system are ongoing.

## Experimental Section

**Materials and methods**: ^1^H NMR spectra were recorded on Bruker AM, DPX, and AV instruments at 250, 300, or 400 MHz and 300 K. ^13^C spectra were recorded on Bruker AM, DPX, and AV instruments at 62.5, 75, 100, or 150 MHz. The chemical shifts *δ* in ^1^H and ^13^C NMR spectra are reported in ppm relative to the solvent signal. The fine structure of proton signals was specified by s (singlet), d (doublet), t (triplet), q (quartet), m (multiplet), dd (doublet of doublets), or br (broad) and in quotations for pseudofine structures. Assignments in the ^1^H and ^13^C NMR spectra were made by DEPT, COSY, HSQC, and HMBC experiments. TLC analysis was carried out on polygram Sil G/UV_254_ by Macherey–Nagel & Co. (Dueren; thickness=0.2 mm), 60 F_254_ (Merck KGaA, Darmstadt; thickness=0.2 mm), or RP-18W (Sigma–Aldrich Chemie GmbH, Steinheim; fluorescence indicator=254 nm, thickness=0.15 mm). Column chromatography was carried out on silica gel 60 (Merck KGaA, Darmstadt; 40–63 μm) at normal pressure or on silica gel 60 (Merck KGaA, Darmstadt; 15–40 μm) at a pressure of 2–3 bar (flash chromatography). FPLC was performed at 4 °C on a Pharmacia FPLC system equipped with a single-path monitor UV-1 UV detector (*λ*=254 nm) and self-packed columns of different sizes with diethylamnoethyl (DEAE) sepharose material for ion-exchange FPLC (Sigma–Aldrich; 0.05 m triethylammonium hydrogencarbonate (TEAB) buffer pH 8.0 (A)/0.8 m TEAB buffer pH 8.0 (B) as the eluent) or octadecyl-functionalised silica gel (Sigma–Aldrich; water (A)/CH_3_CN (B) as the eluent) for RP-FPLC. RP-HPLC was performed on a Jasco LC-2000Plus HPLC system equipped with a Jasco UV 2075Plus detector (detection at *λ*=254 nm) and a Shimadzu RF-353 fluorescence detector (excitation and emission at the specific wavelength of the dye used). For the reversed-phase separation, Phenomenex Jupiter 4μ Proteo 90 A 4 μm columns (250×15 mm for preparative and 250×4.6 mm for analytical separations) were used with 1 m triethylammonium acetate (TEAA) buffer pH 6.5 (A)/water (B)/CH_3_CN (C) as the eluents. UV detection was accomplished at *λ*=254 nm. Ion-exchange HPLC was performed on a Jasco LC-900 HPLC system equipped with a Jasco UV-970 detector (detection at *λ*=254 nm) and a Dionex BioLC DNAPac PA-100 column (250×9 mm) with water/0.25 m tris(hydroxymethyl)aminomethane hydrochloride (Tris⋅HCl) buffer (pH 8)/1 m sodium chloride solution as the eluents. ESI mass-spectrometry was performed on a Fisons instrument equipped with a VG platform II with quadrupol analyser. UV spectroscopy was performed on a Jasco V-650 spectrometer with 0.1 cm cuvettes. Fluorescence spectroscopy was performed on a Hitachi F4500 fluorescence spectrometer with 0.3 cm cuvettes. Elemental analyses were recorded on a Foss-Heraeus CHN-O Rapid instrument. The numbering of the atoms to assign the NMR signal are not related to the IUPAC numbering or the numbers used in the names of the compounds. Some signals in the ^1^H and ^13^C NMR spectra of the triphosphate compounds are doubled, although this did not occur in the spectra of nucleoside derivatives **24**–**27**. We assume that this outcome is due to the diastereoisomers that are obtained by introduction of the linker as a racemic mixture. These nucleoside derivatives are given in the ^13^C NMR data as X.XX/Y.YY.

**General procedure for the Sonogashira coupling (GP2)**: In a heat-gun-dried Schlenk flask in an Ar atmosphere, the nucleoside was dissolved in dry DMF (0.1 mmol mL^−1^) and triethylamine (5 equiv). The mixture was degassed by using the freeze/thaw technique for three times. After the reaction mixture had been warmed to room temperature, CuI (0.2 equiv) and [Pd(PPh_3_)_4_] (0.1 equiv) were added. In a separate heat-gun-dried Schlenk flask, alkyne **23** (1.2–2.0 equiv) was dissolved in dry DMF (250 μL) and added in two portions to the reaction mixture with a syringe. One half portion was added immediately and the other half portion after 1 h. The reaction mixture was stirred for 3.5–5 h at room temperature in the dark. The reaction mixture was evaporated to dryness, the crude product redissolved in CH_2_Cl_2_ (10 mL) and 5 % ethylenediaminetetraacetate (EDTA; 5 mL) solution, and stirred vigorously for 10 min. The layers were separated and the organic layer was washed with 5 % EDTA solution (5 mL), dried over sodium sulphate, and concentrated in vacuum. The residue was purified by flash-column chromatography. For example, the synthesis of 5-(prop-2-ynyl)carbamic acid-1-{2-[2-(2-*tert*-butoxycarbonylaminoethoxy)ethoxy]ethoxymethyl}-2-cyanoethyl ester-3′-*O*-(2-cyanoethyl)-2′-deoxyuridine (**24**).


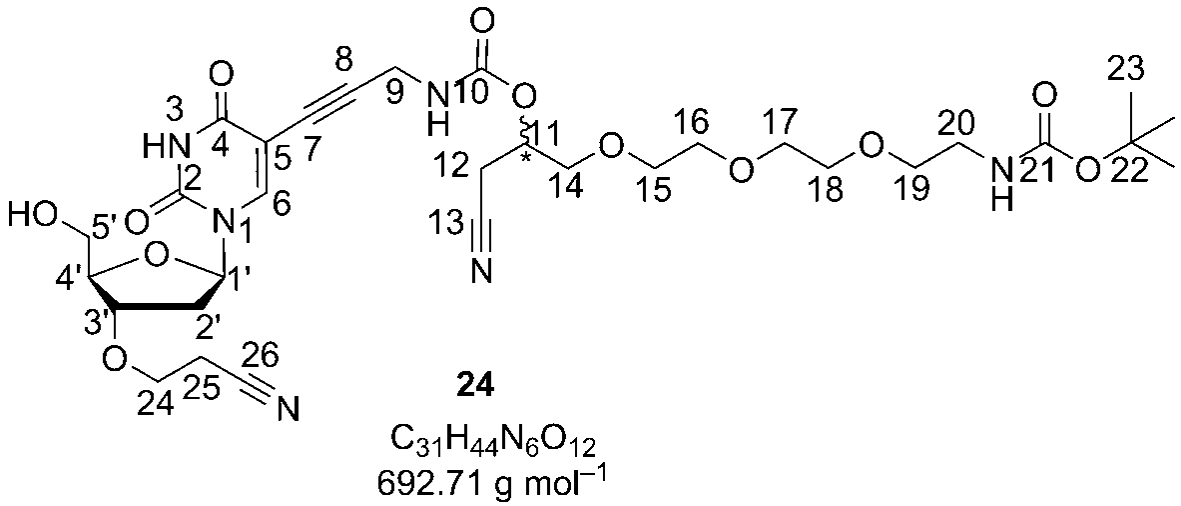


Following GP2, the reaction of 5-iodo-3′-*O*-(2-cyanoethyl)-2′-deoxyuridine (**10**; 250 mg, 0.61 mmol, 1.0 equiv) with alkyne **23** (305 mg, 0.74 mmol, 1.2 equiv) gave, after flash-column chromatography (CH_2_Cl_2_/MeOH 99:1→90:10), **24** as a slightly yellow oil (340 mg, 80 %). *R*_F_=0.34 (CH_2_Cl_2_/MeOH 90:10); ^1^H NMR (300 MHz, [D_6_]DMSO, 300 K): *δ*=1.37 (s, 23-H; 9 H), 2.16–2.31 (m, 2′-H; 2 H), 2.77 (t, 25-H; 2 H), 2.88 (m, 12-H; 2 H), 3.06 (q, 20-H; 2 H), 3.37 (t, 19-H; 2 H), 3.48–3.65 (m, 5′-H, 24-H, 14-H, 15-H, 16-H, 17-H, 18-H; 7×2 H), 3.93–3.97 (m, 4′-H; 1 H), 4.02 (d, 9-H; 2 H), 4.14 (quintet, 3′-H; 1 H), 4.95 (quintet, 11-H; 1 H), 5.13–5.19 (m, 5′-OH; 1 H), 6.07 (t, 1′-H; 1 H), 6.68–6.77 (m, 20-NH; 1 H), 7.93 (t, 9-NH; 1 H), 8.14 (s, 6-H; 1 H), 11.64 ppm (s, N^3^H, *J*_9,9−NH_=5.7, *J*_19,20_=6.0 Hz; 1 H); ^13^C NMR (75 MHz, [D_6_]DMSO, 300 K): *δ*=18.23 (25-C), 19.72 (12-C), 28.21 (23-C), 30.68 (9-C), 36.90 (2′-C), 39.69 (20-C), 61.14 (5′-C), 63.56 (24-C), 67.91 (11-C), 69.17 (19-C), 69.49, 69.60, 69.73, 70.21, 70.30 (14-C, 15-C, 16-C, 17-C, 18-C), 74.49 (7-C), 77.58 (22-C), 79.08 (3′-C), 84.64 (1′-C), 84.89 (4′-C), 89.57 (8-C), 98.23 (5-C), 117.64 (13-C), 119.26 (26-C), 143.40 (2-C), 143.56 (6-C), 154.84 (10-C), 155.54 (21-C), 161.49 ppm (4-C); ESI^+^-MS (*m/z*): calcd: 692.71 [C_31_H_44_N_6_O_12_]; found: 710.5 [*M*+H_2_O]^+^, 715.4 [*M*+Na]^+^; elemental analysis (%) calcd for C_31_H_44_N_6_O_12_: C 53.75, H 6.40, N 12.12; found: C 53.53, H 6.51, N 11.88.

**General procedure for the synthesis of** ***N*****-deprotected triphosphates (GP3)**: The *N*-Boc protected nucleoside was vacuum dried in a Schlenk flask for 2 days, the flask was flushed with argon, and the nucleoside (0.1 mmol) dissolved in dry pyridine (100 μL) and dry dioxane (300 μL). A freshly prepared solution of 2-chloro-4*H*-1,2,3-benzodioxaphosphorin-4-one (1 m, 1.15 equiv) in dry dioxane was added with a syringe and the reaction mixture stirred for 15 min at room temperature. Dry DMF (150 μL), bis(tributylammonium)pyrophosphate (1 m, 1.5 equiv), and tributylamine (1 m, 4.35 equiv) were added simultaneously with syringes. The reaction mixture was stirred for 20 min before a solution of iodine in pyridine/water (2 %, 98:2) was added (1 mL). The reaction mixture was left for a further 20 min. Afterwards, the excess of iodine was quenched with 5 % Na_2_SO_3_ solution, and the mixture was evaporated to dryness. The crude triphosphate derivatives (0.1 mmol) were dissolved in water (5 mL) containing TFA (40 equiv). The solution was stirred for 5–6 h at room temperature and subsequently concentrated in vacuum. For the ion-exchange FPLC purification, the deprotected triphosphate derivatives were dissolved in Millipore water (8–12 mL) and filtered through a 0.45 μm syringe filter. The following gradient was used for the separation at a flow rate of 4 mL min^−1^: 0 % B (0 mL)→50 % B (500 mL)→100 % B (650 mL). The combined fractions were lyophilised, dissolved in 1–2 mL of Millipore water, and filtered through a 0.45 μm syringe filter before the second purification step by preparative RP-HPLC. The following gradient was used at a flow rate of 6 mL min^−1^: 0 min: 10:90:0 A/B/C, 15 min: 10:60:30 A/B/C; 16 min: 0:0:100 A/B/C; 22 min: 0:0:100 A/B/C; 23 min: 10:90 A/B/C, 30 min: 10:90:0 A/B/C. The final yields were calculated from the ^1^H NMR spectrum of the HPLC purified triphosphate derivatives because the products contained traces of triethylammonium acetate after RP-HPLC purification (for example, the synthesis of **28**).


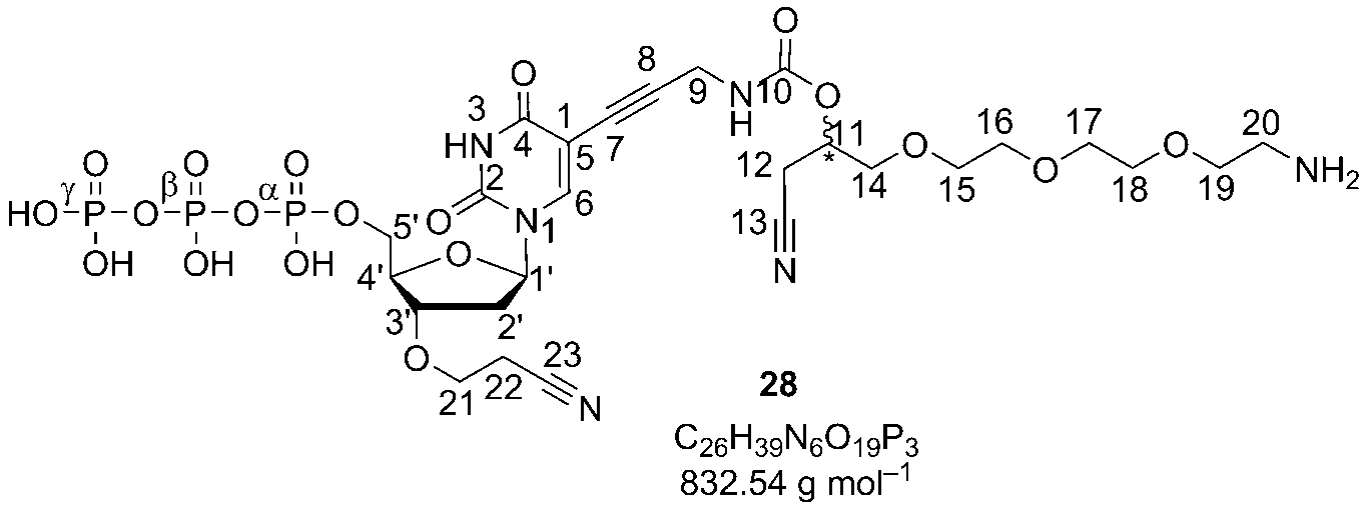


Following GP3, the reaction of the *N*-Boc-protected nucleoside **24** (500 mg, 0.72 mmol, 1.0 equiv) yielded triphosphate **28** as a colourless oil (140 mg, 23 %). *R*_F_=0.39 (*i*Pr/H_2_O/EtMe_2_N 6:3.8:0.2); IE-FPLC: elution concentration=0.29 m TEAB/buffer (32 % B); RP-HPLC: retention time=14.92 min; ^1^H NMR (300 MHz, D_2_O, 300 K): *δ*=2.32 (ddd, 2′-H; 1 H), 2.55 (ddd, 2′-H′; 1 H), 2.81 (t, 22-H; 2 H), 2.89–2.99 (m, 12-H; 2 H), 3.25 (m, 20-H; 2 H), 3.67–3.79 (m, 14-H, 15-H, 16-H, 17-H, 18-H, 19-H; 6×2 H), 3.84 (t, 21-H; 2 H), 4.12–4.26 (m, 5′-H, 9-H; 2×2 H), 4.37 (br s, 4′-H; 1 H), 4.45–4.49 (m, 3′-H; 1 H), 5.12–5.23 (m, 11-H; 1 H), 6.26 (dd, 1′-H; 1 H), 8.17 ppm (s, 6-H, *J*_2′,2′′_=14.5, *J*_1′,2′_=8.4, *J*_1′,2′′_=5.7, *J*_21,22_=6.0 Hz; 1 H); ^13^C NMR (75 MHz, D_2_O, 300 K): *δ*=18.91 (22-C), 20.21 (12-C), 31.49 (9-C), 37.28/37.30 (2′-C), 39.67 (21-C), 64.43 (21-C), 66.31 (d, 5′-C), 66.95, 70.09, 70.12, 70.19, 70.84, 71.18 (14-C, 15-C, 16-C, 17-C, 18-C, 19-C), 69.30 (11-C), 73.98/74.01 (7-C), 80.45/80.47 (3′-C), 84.31 (d, 4′-C), 86.50 (1′-C), 90.93 (8-C), 99.77 (5-C), 118.78 (13-C), 120.45 (23-C), 145.34/145.35 (6-C), 151.03 (2-C), 157.27/157.29 (10-C), 164.99 ppm (4-C, *J*_4′,α−P_=8.82, *J*_5′,α−P_=5.60 Hz); ^31^P NMR (121 MHz, D_2_O, 300 K): *δ*=−10.97 (d, γ-P), −11.69 (d, α-P), −23.43 ppm (t, β-P); ESI^−^-MS (*m/z*): calcd for [C_26_H_39_N_6_O_19_P_3_]: 832.54; found: 831.4 [*M*−H]^−^, 849.6 [*M*−H+H_2_O]^−^.

**Synthesis of 2**: KHCO_3_ (3 mg, 30 μmol, 2.0 equiv) was added to a solution of triphosphate **28** (12 mg, 14 μmol, 1.0 equiv) in dry DMF (500 μL). The reaction mixture was cooled to 0 °C, and 5- and 6-carboxyfluorescein-(*N*-hydroxysuccinimidyl)ester (10 mg, 20 μmol, 1.5 equiv) was dissolved in dry DMF (250 μL) and added in two portions to the reaction mixture, one half immediately and the other after 1 h at 0 °C. The reaction mixture was stirred for 20 h at 0 °C. After evaporation of the solvent, the crude product was purified by RP-FPLC at 4 °C and preparative RP-HPLC (see GP4 in the Supporting Information). The triethylammonium salt of the labelled triphosphate **2** was obtained as a yellow solid (6 mg, 34 %) as a mixture of the two regioisomers, each consisting of two diastereomers. *R*_F_=0.65 (H_2_O/CH_3_CN 80:20); RP-FPLC: elution concentration=2.5→6 % B; RP-HPLC: retention time=16.49 min isomer A, 16.90 min isomer B (UV detection), 16.53 min isomer A, 16.94 min isomer B (fluorescence detection); UV absorption: *λ*=494, 277, 235 nm; fluorescence: *λ*_ex_=492, *λ*_em_=512 nm; ^1^H NMR (250 MHz, D_2_O, 300 K): *δ*=2.15–2.37 (m, 2′-H; 1 H), 2.44–2.59 (m, 2′-H′; 1 H), 2.81–2.93 (m, 12-H, 23-H; 2×2 H), 3.57–3.91 (m, 14-H, 15-H, 16-H, 17-H, 18-H, 19-H, 20-H, 22-H; 8×2 H), 4.00 (br s, 9-H; 2 H), 4.10–4.32 (m, 4′-H, 5′-H; 3 H), 4.10–4.32 (m, 3′-H; 1 H), 4.99–5.14 (m, 11-H; 1 H), 6.04–6.25 (m, 1′-H; 1 H), 6.69–7.04 (m, fluorescein-H; 4 H), 7.09–7.32 (m, fluorescein-H; 2 H), 7.56–8.46 ppm (m, 6-H, fluorescein-H; 5 H); ^31^P NMR (121 MHz, D_2_O, 300 K): *δ*=−10.63 (d, γ-P), −11.56 (dd, α-P), −23.09 ppm (t, β-P, *J*_α,*β*_=19.6, *J*_γ,*β*_=19.6 Hz); ESI^−^-MS (*m/z*): calcd for [C_47_H_49_N_6_O_25_P_3_]: 1190.85; found: 594.6 [*M*−2 H]^2−^, 1110.8 [*M*−PO_3_-H]^−^, 554.8 [*M*−PO_3_-2 H]^2−^, 1190.4 [*M*−H]^−^.


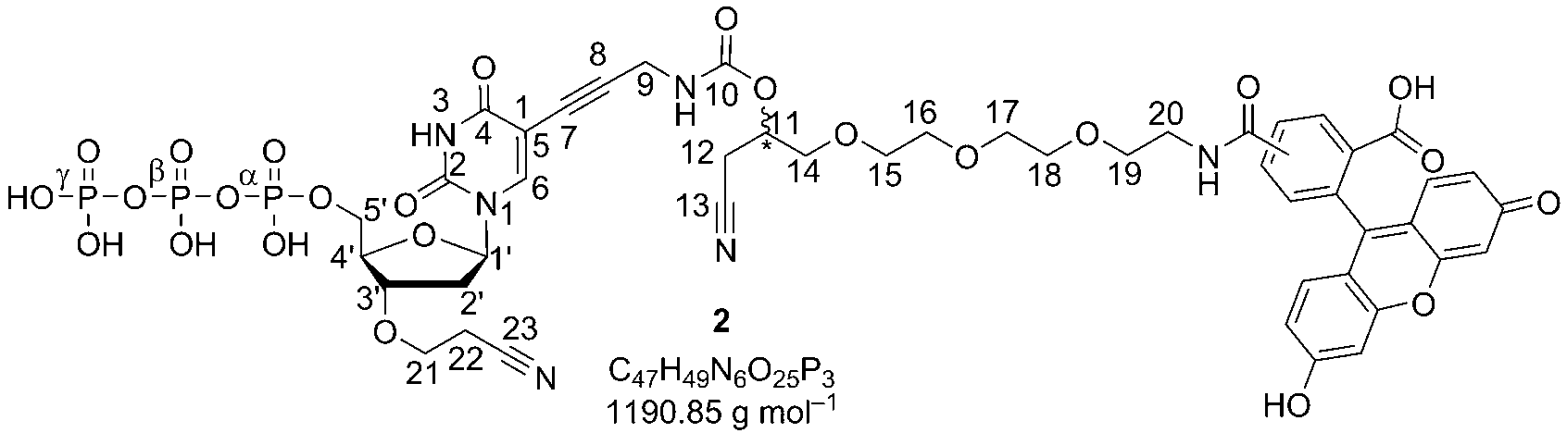


**Solution-based primer-extension experiments**: The oligodeoxynucleotides (shown in Figure 2 A) were used to form the DNA substrate for the extension and termination experiments using 3′-*O*-(2-cyanoethyl)-modified nucleotides and an extension experiment using fluorescence-labelled reversible terminators **2**–**5**. The primer strand was 5′-radiolabelled and annealed to the complementary (template) strand by heating for 5 min to 95 °C and gradually cooling to room temperature over 2 h. For the primer extension, 10 nm DNA duplex, polymerases in at least 10-fold molar excess, and 50 μm of the 3′-modified nucleotides was used. The 3′-modified nucleotides were additionally purified by enzymatic depletion of the natural nucleotide counterpart before use.[41] The reactions were performed for 60 min at 37 °C in a total volume of the reaction mixture of 20 μL, containing 33 mm Tris–acetate (pH 7.9, at 37 °C), 10 mm magnesium acetate, 66 mm potassium acetate, and BSA (0.1 mg mL^−1^). After completion of the reaction, an aliquot of the reaction mixture was supplemented by dTTP (up to 50 μm), and the reaction was allowed to proceed for additional 5 min at the same temperature. The reactions were stopped by adding an equal volume of the STOP solution: 95 % formamide and 100 mm EDTA. The products were resolved by using a denaturing polyacrylamide (PAA) gel (7 m urea; 15 % 29:1). The gel was dried on Whatman paper and autoradiographed by using a Fuji phosphorimager screen. For the evaluation of the polymerase specificity, the four substrate systems were used (Figure 3 B). Concentrations of 5 nm of each DNA duplex and 20 U μL^−1^ of the RevertAid M-MuLV reverse transcriptase were used. The reaction buffer was the same as described above with the addition of MnCl_2_ (1 mm) and dithiothreitol (DTT; 5 mm). The reactions were performed for 5 min at 37 °C, then an aliquot of the reaction mixture was supplemented with all four natural dNTP derivatives (up to 100 μm each). The reaction was allowed to proceed for additional 5 min at the same temperature. Samples were analysed as described above.

Spotting of DNA arrays and APEX reactions on CodeLink arrays with fluorescently labelled 3′-*O*-(2-cyanoethyl)-dNTP derivatives. All oligonucleotide primers were diluted to a concentration of 10 μm in carbonate buffer (100 mm, pH 9.0) and spotted onto CodeLink-activated slides from SurModics (Eden Prairie, MN, USA) with a Versarray Chipwriter Pro Arrayer (BioRad Laboratories, Hercules, CA, USA) as 8×8-probe squares according to the scheme shown in Figure 4. The oligonucleotide primers were immobilised onto the slide surface and the slides blocked before the APEX reactions, as suggested by the vendor. APEX reaction cycles on CodeLink arrays consisted of the following steps: 1) Blocking of DNA arrays with blocking solution (50 μL, Tris-acetate (33 mm, pH 7.9 at 37 °C); magnesium acetate (10 mm), potassium acetate (66 mm), BSA (1 mg mL^−1^), DTT (10 mm), Tween-20 (1 %), NP-40 (1 %)) for 10 min at 37 °C. 2) The first primer-extension reaction with **2**–**5** was carried out by treating the slide with the set of the fluorescently labelled reversible terminators **2**–**5** and RevertaAid M-MuLV reverse transcriptase at 37 °C for 30 min. An aliquot of the reaction mixture (35 μL) was used for one slide containing Tris–acetate (33 mm, pH 7.9 at 37 °C), magnesium acetate (10 mm), potassium acetate (66 mm), BSA (0.1 mg mL^−1^), MnCl_2_ (1 mm), DTT (10 mm), Tween-20 (0.5 %), Nonidet P-40 (0.5 %), 2-hydroxymethyl[18]crown-6 (1 %), **2**–**5** (100 μm each), and RevertAid M-MuLV reverse transcriptase (10 U μL^−1^). 3) Blocking of the starting-material oligonucleotide primers on the array was accomplished with TdT (MBI Fermentas, Vilnius, Lithuania) and ddATP by incubating the arrays for 20 min at 37 °C. The TdT reaction mixture consisted of the reaction mixture with an aliquot (40 μL) of potassium cacodylate (200 mm), Tris (25 mm), Triton X-100 (0.01 %, v/v), CoCl_2_ (1 mm, pH 7.2 at 25 °C), ddATP (1 mm), and TdT enzyme (80 U). 4) The reactions were stopped by washing with deionized water at 95 °C. The slides were dried, covered with a droplet of Slowfade light antifade reagent (Molecular Probes, OR, USA), and imaged with Genorama QuattroImager detector (Asper Biotech Ltd.). The fluorescence signal intensities were extracted and analysed with BaseCaller module of the Genorama genotyping software package (Asper Biotech Ltd.). 5) Cleavage of the fluorescence dye and the 3′-*O*-(2-cyanoethyl) group from the incorporated fluorescence labelled 3′-*O*-(2-cyanoethyl)-blocked nucleotides was accomplished with TBAF (1 m) in THF. Before the unblocking reaction, the DNA arrays were washed three times with THF. The unblocking reaction was carried out by immersing the DNA arrays for 15 min at 45 °C into a 1:1 solution of TBAF/THF (Fluka, Buchs, Switzerland) and dry DMF. 6) The DNA arrays were washed with deionized water, dried, and read with the Genorama QuattroImager detector to be sure that the unblocking reaction was successful. 7) Before the second primer-extension reaction, the slides were blocked again with blocking solution (see above, step 1) for 10 min at 37 °C. 8) The second primer-extension reaction using the fluorescently labelled 3′-*O*-(2-cyanoethyl)-dNTP derivatives **2**–**5** and the reverse transcriptase was carried out under the reaction conditions described for the first extension step (step 2). 9) The reactions were stopped by washing with deionized water at 95 °C. The slides were dried, read, and analysed as described above for first primer-extension reaction.
