# Supplementary material for: Fluoride-Cleavable, Fluorescently Labelled Reversible Terminators: Synthesis and Use in Primer Extension
Source: Chemistry. 2011 Feb 3;17(10):2903–15. doi: 10.1002/chem.201001952 (PMC3110862; doi:10.1002/chem.201001952)

CHEMISTRY
A EUROPEAN JOURNAL

Supporting Information

© Copyright Wiley-VCH Verlag GmbH & Co. KGaA, 69451 Weinheim, 2011

Fluoride-Cleavable, Fluorescently Labelled Reversible Terminators: Synthesis and Use in Primer Extension

Diana C. Knapp,[a] Saulius Serva,[b] Jennifer D′Onofrio,[a] Angelika Keller,[a] Arvydas Lubys,[b] Ants Kurg,[c, d] Maido Remm,[d] and Joachim W. Engels*[a]

chem_201001952_sm_miscellaneous_information.pdf

Supporting information

1 EXPERIMENTAL DATA – CHEMICAL PART 4

1.1 Materials and methods 4

1.2 Introduction of the protecting groups 6

1.2.1 Synthesis of 6-*N*-(*N,N*-dimethylaminomethylidenyl)-7-deaza-7-iodo-2'-deoxyadenosine 14 6

1.2.2 Synthesis of 6-*N*-(*N,N*-dimethylaminomethylidenyl)-7-deaza-7-iodo-5'-*O*-bezoyl-2'-deoxyadenosine 15 7

1.2.3 Synthesis of 4-*N-(N,N*-dimethylaminomethylidenyl)-5-iodo-5'-*O*-benzoyl-2'-deoxycytidine 12 8

1.2.4 Synthesis of 2-*N*-(*N,N*-dimethylaminomethylidenyl)-7-deaza-7-iodo-3',5'-*O*-(tetraisopropyldisiloxane-1,3-diyl)-2'-deoxyguanosine 17 9

1.2.5 Synthesis of 2-*N*-(*N,N*-dimethylaminomethylidenyl)-N^1-benzoyl-7-deaza-7-iodo-3',5'-*O*-(tetraisopropyldisiloxane-1,3-diyl)-2'-deoxyguanosine 32 10

1.2.6 Synthesis of 2-*N*-(*N,N*-dimethylaminomethylidenyl)-N^1-benzoyl-7-deaza-7-iodo-2'-deoxyguanosine 18 11

1.2.7 Synthesis of 2-*N*-(*N,N*-dimethylaminomethylidenyl)-N^1-benzoyl-7-deaza-7-iodo-5'-*O*-(4-monomethoxytrityl)-2'-deoxyguanosine 19 12

1.3 Introduction of the 3'-*O*-(2-cyanoethyl) group and deprotection 13

1.3.1 General procedure for the introduction of the 2-cyanoethyl group at the 3'-OH position (GP 1) 13

1.3.2 Synthesis of 6-*N*-(*N,N*-dimethylaminomethylidenyl)-7-deaza-7-iodo-3'-(2-cyanoethyl)-5'-*O*-bezoyl-2'-deoxyadenosine 33 13

1.3.3 Synthesis of 7-deaza-7-iodo-3'-(2-cyanoethyl)-2'-deoxyadenosine 16 14

1.3.4 Synthesis of 4-*N-(N,N*-dimethylaminomethylidenyl)-5-iodo-5'-*O*-benzoyl-3'-*O*-(2-cyanoethyl)-2'-deoxycytidine 34 15

1.3.5 Synthesis of 5-Iodo-3'-*O*-(2-cyanoethyl)-2'-deoxycytidine 13 16

1.3.6 Synthesis of 2-*N*-(*N,N*-dimethylaminomethylidenyl)-N^1-benzoyl-7-deaza-7-iodo-3'-*O*-(2-cyanoethyl)-5'-*O*-(4-monomethoxytrityl)-2'-deoxyguanosine 20 16

1.3.7 Synthesis of 2-*N*-(*N,N*-dimethylaminomethylidenyl)-N^3-benzoyl-7-deaza-7-iodo-3'-*O*-(2-cyanoethyl)-2'-deoxyguanosine 35 17

1.3.8 Synthesis of 7-Deaza-7-iodo-3'-*O*-(2-cyanoethyl)-2'-deoxyguanosine 21 18

1.4 Synthesis and attachment of the linker 19

1.4.1 Synthesis of the alkyne modified linker Prop-2-inylcarbamic acid 1-{2-[2-(2-*tert*-butoxycarbonylaminoethoxy)ethoxy]ethoxymethyl}-2-cyanoethyl ester 23 19
1.4.2 Synthesis of 7-Deaza-7-(prop-2-ynyl)carbamic acid-1-{2-[2-(2-*tert*-butoxycarbonylaminoethoxy)ethoxy]ethoxymethyl}-2-cyanoethyl ester-3'-*O*-(2-cyanoethyl)-2'desoxyadenosine 26 20
1.4.3 Synthesis of 5-(Prop-2-ynyl)carbamic acid-1-{2-[2-(2-*tert*-butoxycarbonylaminoethoxy)ethoxy]ethoxymethyl}-2-cyanoethyl ester-3'-*O*-(2-cyanoethyl)-2'-deoxycytidine 25 21
1.4.4 Synthesis of 7-Deaza-7-(prop-2-ynyl)carbamic acid-1-{2-[2-(2-*tert*-butoxycarbonylaminoethoxy)ethoxy]ethoxymethyl}-2-cyanoethyl ester-3'-*O*-(2-cyanoethyl)-2'-desoxyguanosine 27 22

1.5 Triphosphate synthesis and attachment of the dye 23

1.5.1 Synthesis of 7-Deaza-7-(prop-2-ynyl)carbamic acid-1-{2-[2-(2-*tert*-butoxycarbonylaminoethoxy)ethoxy]ethoxymethyl}-2-cyanoethyl ester-3'-*O*-(2-cyanoethyl)-2'-deoxyadenosine 5'-triphosphate 30 23
1.5.2 Synthesis of 5-(Prop-2-ynyl)carbamic acid-1-{2-[2-(2-*tert*-butoxycarbonylaminoethoxy)ethoxy]ethoxymethyl}-2-cyanoethyl ester-3'-*O*-(2-cyanoethyl)-2'-deoxycytidine 5'-triphosphate 29 24
1.5.3 Synthesis of 7-Deaza-7-(prop-2-ynyl)carbamic acid-1-{2-[2-(2-*tert*-butoxycarbonylaminoethoxy)ethoxy]ethoxymethyl}-2-cyanoethyl ester-3'-*O*-(2-cyanoethyl)-2'-deoxyguanosine 5'-triphosphate 31 25
1.5.4 General procedure for the purification of labelled triphosphates (GP 4) 26
1.5.5 Synthesis of 7-Deaza-7-(prop-2-ynyl)carbamic acid-1-{2-[2-(2-5(6)-carboxyTexasRed-amidoethoxy)ethoxy]ethoxymethyl}-2-cyanoethyl ester-3'-*O*-(2-cyanoethyl)-2'-desoxyadenosine 5'-triphosphate 3 26
1.5.6 Synthesis of 5-(Prop-2-ynyl)carbamic acid-1-{2-[2-(2-Cy3-amidoethoxy)ethoxy]ethoxymethyl}-2-cyanoethyl ester-3'-*O*-(2-cyanoethyl)2'-deoxycytidine 5'-triphosphate 4 27
1.5.7 Synthesis of 7-Deaza-7-(prop-2-ynyl)carbamic acid-1-{2-[2-(2-Cy5-amidoethoxy)ethoxy]ethoxymethyl}-2-cyanoethyl ester-3'-*O*-(2-cyanoethyl)-2'-desoxyguanosine 5'-triphosphate 5 28

2 SPECTRA AND CHROMATOGRAMS 29

2.1 5-(Prop-2-ynyl)carbamic acid-1-{2-[2-(2-amino-ethoxy)ethoxy]ethoxymethyl}-2-cyanoethyl ester-3'-*O*-(2-cyanoethyl)2'-desoxyuridine 5'-triphosphate 28 29

2.2 5-(Prop-2-ynyl)carbamic acid-1-{2-[2-(2-5(6)-carboxyfluoresceinamidoethoxy)ethoxy]ethoxymethyl}-2-cyanoethyl ester-3′-*O*-(2-cyanoethyl)-2′-deoxyuridine 5′-triphosphate 2 33

2.3 7-Deaza-7-(prop-2-ynyl)carbamic acid-1-{2-[2-(2-*tert*-butoxycarbonylaminoethoxy)ethoxy]ethoxy-methyl}-2-

	**cyanoethyl ester-3'-*O*-(2-cyanoethyl)-2'-desoxyadenosine 5'-triphosphate 30**	**37**
**2.4**	**7-Deaza-7-(prop-2-ynyl)carbamic acid-1-{2-[2-(2-5(6)-carboxyTexasRed-amidoethoxy)ethoxy]-ethoxymethyl}-2-cyanoethyl ester-3'-*O*-(2-cyanoethyl)-2'-desoxyadenosine 5'-triphosphate 3**	**41**
**2.5**	**5-(Prop-2-ynyl)carbamic acid-1-{2-[2-(2-*tert*-butoxycarbonylaminoethoxy)ethoxy]-ethoxymethyl}-2-cyanoethyl ester-3'-*O*-(2-cyanoethyl)-2'-deoxycytidine 5'-triphosphate 29**	**45**
**2.6**	**5-(Prop-2-ynyl)carbamic acid-1-{2-[2-(2-Cy3-amidoethoxy)ethoxy]ethoxymethyl}-2-cyanoethyl ester-3'-*O*-(2-cyanoethyl)2'-deoxycytidine 5'-triphosphate 4**	**49**
**2.7**	**7-Deaza-7-(prop-2-ynyl)carbamic acid-1-{2-[2-(2-*tert*-butoxycarbonylaminoethoxy)ethoxy]ethoxymethyl}-2-cyanoethyl ester-3'-*O*-(2-cyanoethyl)-2'-desoxyguanosine 5'-triphosphate 31**	**53**
**2.8**	**7-Deaza-7-(prop-2-ynyl)carbamic acid-1-{2-[2-(2-Cy5-amidoethoxy)ethoxy]ethoxymethyl}-2-cyanoethyl ester-3'-*O*-(2-cyanoethyl)-2'-desoxyguanosine 5'-triphosphate 5**	**57**

1 Experimental data – chemical part

1.1 Materials and methods

The following devices and methods were used for chemical analysis:

Nuclear Magnetic Resonance spectroscopy: ^{1}H-NMR spectra were recorded on Bruker AM, DPX and AV instruments at 250, 300 or 400 MHz and 300 K. ^{13}C spectra were recorded on Bruker AM, DPX and AV instruments at 62.5, 75, 100 or 150 MHz. Chemical shifts (δ) in ^{1}H-NMR and ^{13}C-NMR spectra are reported in ppm relative to the solvent signal. The fine structure of proton signals was specified by s (singlet), d (doublet), t (triplet), q (quartet), m (multiplet), dd (doublet of doublet), br (broad) and in quotations for pseudo fine structures. Assignments in the ^{1}H-NMR and ^{13}C-NMR spectra were made by DEPT, COSY, HSQC and HMBC experiments.

Thin Layer chromatography (TLC): Recorded on Polygram® Sil G/UV_{254} by Macherey Nagel & Co., Dueren (thickness of layer 0.2 mm), 60 F_{254} from Merck KGaA, Darmstadt (thickness of layer 0.2 mm) or RP-18W with fluorescence indicator 254 nm from Sigma-Aldrich Chemie GmbH, Steinheim (thickness of layer 0.15 mm).

Column-chromatography was carried out on silica gel 60 (40-63 µm) by Merck KGaA, Darmstadt at normal pressure or on silica gel 60 (15-40 µm) by Merck KGaA, Darmstadt at a pressure of 2-3 bar (**flash-chromatography**).

Fast Protein Liquid Chomatography® (FPLC®) was performed at 4 °C on a Pharmacia FPLC® system equipped with a Single Path Monitor UV-1 UV detector (254 nm) and self-packed columns of different sizes with DEAE Sepharose® material from Sigma-Aldrich for ion-exchange FPLC® (0.05 M triethylammonium hydrogencarbonate (TEAB)-buffer pH = 8.0 (A)/0.8 M TEAB-buffer pH = 8.0 (B) as eluent) or Octadecyl-functionalised silica gel from Sigma-Aldrich (water (A)/CH_3CN (B) as eluent) for reversed-phase FPLC®.

Reversed-phase (RP) HPLC was performed on a Jasco LC-2000Plus HPLC system equipped with a Jasco UV 2075Plus detector (detection at 254 nm) and a Shimadzu RF-353 fluorescence detector (excitation and emission at the specific wavelength of the dye used). For reversed-phase separation Phenomenex Jupiter 4µ Proteo 90A 4 µm columns (250x15 mm for preparative and 250x4.6 mm for analytical separations) were used with 1 M triethylammonium acetate (TEAA)-buffer pH = 6.5 (A)/water (B)/CH_3CN (C) as eluents. UV detection was accomplished at 254 nm.

Ion-exchange HPLC was performed on a Jasco LC-900 HPLC system equipped with a Jasco UV-970 detector (detection at 254 nm) and a Dionex BioLC® DNAPac® PA-100 (250x9 mm) using water/0.25 M tris(hydroxymethyl)-aminomethane hydrochloride (Tris-Cl) buffer pH = 8/1 M sodium chloride solution as eluents.

Mass spectrometry (MS): ESI-MS was performed on a Fisons instrument equipped with a VG platform II with quadrupol analyser.
UV spectroscopy was performed on a Jasco V-650 spectrometer using 0.1 cm cuvettes.
Fluorescence spectroscopy was performed on a Hitachi F4500 fluorescence spectrometer using 0.3 cm cuvettes.
Elemental analyses (EA) were recorded on a Foss-Heraeus CHN-O Rapid instrument.
The **numbering of the atoms** to assign the NMR signals, are not related to the IUPAC numbering or the numbers used in the names of the compounds.
Abbreviations used: arrayed primer extension (APEX), benzoyl (chloride) (Bz(Cl)), *tert*-butoxycarbonyl (Boc), bovine serum albumin (BSA), calculated (calc.); 2-cyanoethyl (CE), 2'-deoxynucleoside-5'-triphosphate (dNTP), 2'-deoxyadenosine-5'-triphosphate (dATP), 2'-deoxycytidine-5'-triphosphate (dCTP), 2'-deoxyguanosine-5'-triphosphate (dGTP), 2'-deoxythymidine-5'-triphosphate (dTTP), 2',3'-didesoxyadenosine-5'-triphosphate (ddATP), diethylaminoethyl (DEAE), *N,N'*-hydroxysuccinimidyl carbonate (DSC), *N,N*-dimethylaminopyridine (DMAP), *N,N*-dimethylformamide (DMF), dimethylsulfoxide (DMSO), dithiothreitol (DTT), elemental analysis (EA), electrospray ionisation mass spectroscopy (ESI-MS), ethylenediaminetetraacetate (EDTA), fast protein liquid chromatography (FPLC), high-performance liquid chromatography (HPLC), ion-exchange (IE), 4-monomethoxytrityl (chloride (MMTr(Cl)), nuclear magnetic resonance (NMR), polyacylamide (PAA), reversed-phase (RP), single nucleotide polymorphism (SNP), terminal deoxynucleotidyl transferase (TdT), tetrabutylammonium fluoride (TBAF), *p*-toluenesulfonic acid (PTSA), triethylammonium acetate (TEAA), triethylammonium hydrogencarbonate (TEAB), trifluoroacetic acid (TFA), tetrahydrofuran (THF), thin layer chromatography (TLC).

5-Iodo-2'-deoxyuridine **6** was purchased from Pharma-Waldhof GmbH, Düsseldorf, Germany. 7-Iodo-7-deaza-2'-deoxyguanosine **9** was purchased from ChemBiotech GmbH, Münster, Germany.

1.2 Introduction of the protecting groups

1.2.1 Synthesis of 6-*N*-(*N,N*-dimethylaminomethylidenyl)-7-deaza-7-iodo-2'-deoxyadenosine 14

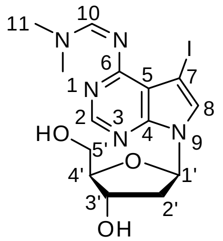

14

$C_{14}H_{18}IN_5O_3$
431.23 g/mol

7-Deaza-7-iodo-2'-deoxyadenosine **8** (2.5 g, 6.6 mmol, 1.0 eq.) was dissolved in 40 mL of DMF and treated with 15 mL (9.9 mmol, 1.5 eq.) of *N*,*N*-dimethylformamide dimethyl acetal and the reaction was stirred at 50 °C for 2 h. Thereafter the reaction was concentrated under reduced pressure and purified by silica gel chromatography (CH_2Cl_2/MeOH = 95:5 to CH_2Cl_2/MeOH = 90:10) affording 2.9 g (95 %) of compound **14** as yellow solid.

TLC: $\mathbf{R_F}$ = 0.27 (CH_2Cl_2/MeOH = 90:10); **^{1}H-NMR** (100 MHz, DMSO-d$_6$, 300 K): δ = 2.16 (m, 2'-H), 2'-H' signal is hidden under DMSO residual signal, 3.18 (s, 11-H_3), 3.23 (s, 11-H_3'), 3.55 (m, 5'-H_2), 3.82 (br. s, 4'-H), 4.34 (br. s, 3'-H), 5.03 (br. t, 5'-O*H*), 5.26 (d, 3'-O*H*), 6.53 (t, 1'-H), 7.71 (s, 8-H), 8.32 (s, 2-H), 8.83 (s, 10-H) ppm; **^{13}C-NMR** (100 MHz, DMSO-d$_6$, 300 K): δ = 34.77 (11-C), 39.72 (2'-C), 40.27 (11-C'), 53.48 (7-C), 61.80 (5'-C), 70.88 (3'-C), 82.80 (1'-C), 87.31 (4'-C), 110.08 (5-C), 128.39 (8-C), 150.67 (4-C), 151.27 (2-C), 156.02 (10-C), 159.98 (6-C) ppm; **ESI(+)-MS** (*m/z*): calc.: 431.04 [$C_{14}H_{18}IN_5O_3$], found: 431.8 $[M+H]^+$.

1.2.2 Synthesis of 6-*N*-(*N,N*-dimethylaminomethylidenyl)-7-deaza-7-iodo-5'-*O*-bezoyl-2'-deoxyadenosine 15

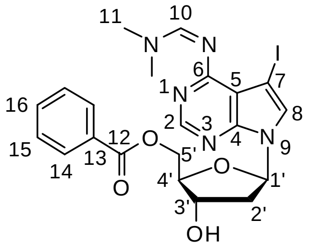

15

$C_{21}H_{22}IN_5O_4$
535.33 g/mol

Compound **14** (2.9 g, 6.2 mmol, 1.0 eq.) was dissolved in 150 mL of dry pyridine and cooled to -15 °C. Benzoyl chloride (700 µL, 6.6 mmol, 1.06 eq.) was dissolved in 40 mL of dry CH_2Cl_2, added to the reaction mixture and the mixture was stirred at -15 °C for 1 h. The reaction was quenched with MeOH, concentrated under reduced pressure and purified by silica gel chromatography (CH_2Cl_2/MeOH = 98:2 to CH_2Cl_2/MeOH = 90:10) afferding 3.0 g (5.6 mmol, 89 %) of compound **15** as slightly yellow solid.

TLC: $\mathbf{R_F}$ = 0.45 (CH_2Cl_2/MeOH = 95:5); **^{1}H-NMR** (400 MHz, DMSO-d_6, 300 K): δ = 2.30 (ddd, 2'-H), 2.66 (m, 2'-H'), 3.18 (s, 11-H_3), 3.22 (s, 11-H_3'), 4.11 (q, 4'-H), 4.41 (dd, 5'-H), 4.52 (m, 3'-H, 5'-H'), 5.52 (d, 3'-O*H*), 6.58 (t, 1'-H), 7.55 (t, 15-H_2), 7.64 (s, 8-H), 7.67 (t, 16-H), 7.98 (d, 14-H_2), 8.32 (s, 2-H), 8.83 (s, 10-H) ppm, $J_{15,16}$ = 7.38 Hz; **^{13}C-NMR** (100 MHz, DMSO-d_6, 300 K): δ = 34.76 (11-C), 38.67 (2'-C), 40.27 (11-C), 54.01 (7-C), 64.43 (5'-C), 70.45 (3'-C), 82.44 (1'-C), 83.51 (4'-C), 110.07 (5-C), 128.11 (8-C), 128.76 (15-C), 129.09 (14-C), 129.32 (13-C), 133.34 (16-C), 150.81 (4-C), 151.45 (2-C), 152.02 (10-C), 160.01 (6-C), 165.48 (12-C) ppm; **ESI(+)-MS** (*m/z*): calc.: 535.07 [$C_{21}H_{22}IN_5O_4$], found: 535.9 $[M+H]^+$; **EA** (%): calc.: C: 47.12, H: 4.14, N: 13.08, found: C: 46.95, H: 4.29, N: 12.85.

1.2.3 Synthesis of 4-*N*-(*N,N*-dimethylaminomethylidenyl)-5-iodo-5'-*O*-benzoyl-2'-deoxycytidine 12

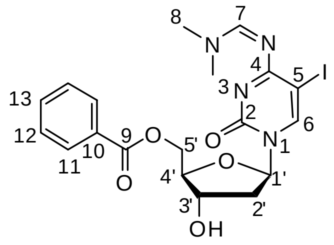

12

$C_{19}H_{21}IN_4O_5$
512.30 g/mol

Well dried *N*[4]-(*N,N*-dimethylaminomethylidenyl)-5-iodo-2'-deoxy-cytidine **11** (1.0 g, 2.45 mmol, 1.0 eq.) was dissolved in 45 mL of dry pyridine and 10 mL of dry DMF. The pale-yellow solution was cooled to 0 °C and 385 µL (3.28 mmol, 1.3 eq.) of benzoyl chloride in 4 mL of dry pyridine were added drop wise via a syringe within 2 h. After stirring the mixture under an Ar atmosphere for further 30 min at room temperature, the solvents were evaporated and the yellow oily residue was purified by column chromatography (CH_2Cl_2/MeOH = 95:5 to CH_2Cl_2/MeOH = 90:10) yielding 750 mg (1.46 mmol, 60 %) of compound **12** as colourless solid.

TLC: $\mathbf{R_F}$ = 0.22 (CH_2Cl_2/MeOH = 90:10); ^{1}**H-NMR** (400 MHz, DMSO-d_6, 300 K): δ = 2.12-2.33 (m, 2'-H_2), 3.12 (s, 8-H_3), 3.21 (s, 8-H_3'), 4.16 (m, 4'-H), 4.35 (m, 3'-H), 4.51 (m, 5'-H_2), 5.45 (d, 3'-O*H*), 6.14 („t“, 1'-H), 7.55 (t, 12-H_2), 7.68 (t, 13-H), 8.01 (d, 11-H_2), 8.07 (s, 6-H), 8.56 (s, 7-H), ppm; ^{13}**C-NMR** (100 MHz, DMSO-d_6, 300 K): δ = 34.92 (8-C), 40.16 (2'-C), 40.93 (8-C'), 64.50 (5'-C), 69.17 (5-C), 70.51 (3'-C), 84.40 (4'-C), 86.13 (1'-C), 128.96 (12-C), 129.28 (11-C), 130.51 (10-C), 133.52 (13-C), 146.59 (6-C), 154.05 (2-C), 158.32 (7-C), 165.64 (9-C), 168.11 (4-C) ppm; **ESI(+)-MS** (*m/z*): calc.: 512.30 [$C_{19}H_{21}IN_4O_5$], found: 513.0 $[M+H]^+$.

1.2.4 Synthesis of 2-*N*-(*N,N*-dimethylaminomethylidenyl)-7-deaza-7-iodo-3',5'-*O*-(tetraisopropyldisiloxane-1,3-diyl)-2'-deoxyguanosine 17

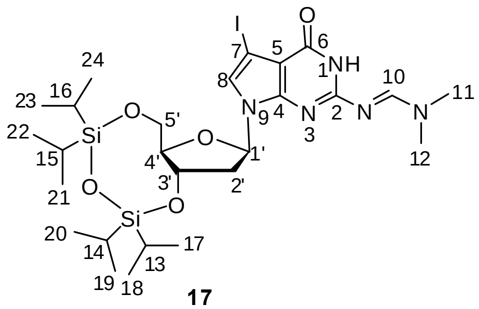

17

$C_{26}H_{44}IN_5O_5Si_2$
689.73 g/mol

7-Deaza-7-iodo-2'-deoxyguanosine **9** (1.0 g, 2.55 mmol, 1.0 eq.) was co-evaporated three times with dry pyridine, then dissolved in 10 mL of dry pyridine and cooled down to 0 °C. 1,1,3,3-Tetraisopropyldichlordisiloxane (0.88 mL, 2.81 mmol, 1.1 eq.) was added and the mixture was stirred under an Ar atmosphere for 30 min at 0 °C and for 20 h at room temperature. The reaction was stopped by addition of 10 mL of MeOH and the resulting mixture stirred for 15 min. The solvents were removed in vacuum, the oily residue was dissolved in 15 mL of dry DMF again and treated with 1.7 mL (12.8 mmol, 5.0 eq.) of *N,N*-dimethylformamide dimethylacetal. The clear solution was stirred 24 h at room temperature under an Ar atmosphere. After completion of the reaction, the mixture was concentrated and the crude product purified by column chromatography (CH_2Cl_2/MeOH = 95:5 to CH_2Cl_2/MeOH = 90:10) yielding 1.73 g (95 %) of compound **17** as yellowish foam.

TLC: $\mathbf{R_F}$ = 0.20 (CH_2Cl_2/MeOH = 95:5); **^{1}H-NMR** (300 MHz, DMSO-d_6, 300 K): δ = 0.99-1.15 (m, 13-H, 14-H, 15-H, 16-H, 17-H_3, 18-H_3, 19-H_3, 20-H_3, 21-H_3, 22-H_3, 23-H_3, 24-H_3), 2.42 (m, 2'-H_2), 3.02, 3.14 (s, 11-H_3, 12-H_3), 3.71 (m, 4'-H), 3.89 (d, 5'-H_2), 4.67 (q, 3'-H), 6.36 (dd, 1'-H), 7.12 (s, 8-H), 8.57 (s, 10-H), 11.09 (s, N^1*H*) ppm; **^{13}C-NMR** (75 MHz, DMSO-d_6, 300 K): δ = 11.86, 12.05, 12.40, 12.62 (13-C, 14-C, 15-C, 16-C), 16.66, 16.73, 16.76, 16.90, 17.04, 17.15, 17.17, 17.19 (17-C, 18-C, 19-C, 20-C, 21-C, 22-C, 23-C, 24-C), 34.45, 40.41 (11-C, 12-C), 40.26 (2'-C), 55.35 (7-C), 61.85 (5'-C), 70.55 (3'-C), 80.30 (1'-C), 83.89 (4'-C), 102.60 (5-C), 122.46 (8-C), 149.02 (4-C), 156.15 (2-C), 157.54 (10-C), 158.72 (6-C) ppm; **ESI(+)-MS** (*m/z*): calc.: 689.19 [$C_{26}H_{44}IN_5O_5Si_2$], found.: 690.1 $[M+H]^+$; **EA** (%): calc.: C: 45.28, H: 6.43, N: 10.15, found: C: 45.45, H: 6.47, N: 10.12.

1.2.5 Synthesis of 2-*N*-(*N,N*-dimethylaminomethylidenyl)-*N*[1]-benzoyl-7-deaza-7-iodo-3',5'-*O*-(tetraisopropyldisiloxane-1,3-diyl)-2'-deoxyguanosine 32

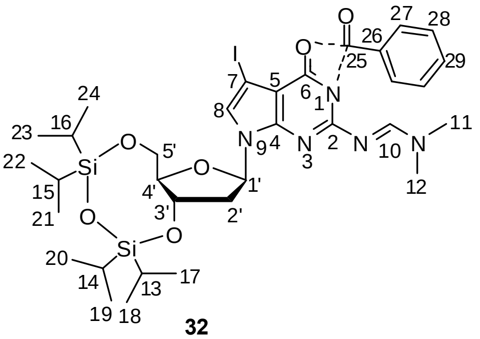

32

$C_{33}H_{48}IN_5O_6Si_2$
793.84 g/mol

Compound **17** (1.5 g, 2.17 mmol, 1.0 eq.) was dissolved in 15 mL of dry pyridine and the reaction was cooled to 0 °C. Benzoyl chloride (514 µL, 4.42 mmol, 2.0 eq.) was dissolved in 500 µL of dry CH_2Cl_2 and added drop wise. The reaction was warmed to room temperature and stirred for 2 h. After 2 h another 100 µL (0.86 mmol, 0.4 eq.) of benzoyl chloride were added and the reaction mixture stirred at room temperature for 18 h. The reaction was quenched by addition of MeOH, concentrated under reduced pressure and purified by flash-column chromatography (CH_2Cl_2/MeOH = 99:1 to CH_2Cl_2/MeOH = 98:2) affording 1.2 g (69 %) of compound **32** as colourless foam.

TLC: $\mathbf{R_F}$ = 0.61 (CH_2Cl_2/MeOH = 95:5); **^{1}H-NMR** (300 MHz, DMSO-d_6, 300 K): δ = 1.13-1.01 (m, 13-H, 14-H, 15-H, 16-H, 17-H_3, 18-H_3, 19-H_3, 20-H_3, 21-H_3, 22-H_3, 23-H_3, 24-H_3), 2'-H_2 signals are hidden below the DMSO residual peak, 2.62, 3.08 (s, 11-H_3, 12-H_3) 3.76 (m, 4'-H), 3.96 (d, 5'-H_2), 4.71 (q, 3'-H), 6.44 (dd, 1'-H), 7.25 (s, 8-H), 7.54 (t, 28-H_2), 7.69 (t, 29-H), 7.78 (d, 27-H_2), 8.60 (s, 10-H) ppm.; **^{13}C-NMR** (75 MHz, DMSO-d_6, 300 K): δ = 11.87, 12.06, 12.41, 12.64, (13-C, 14-C, 15-C, 16-C), 16.66, 16.73, 16.78, 16.92, 17.04, 17.16, 17.19, 17.21 (17-C, 18-C, 19-C, 20-C, 21-C, 22-C, 23-C, 24-C), 34.23, 40.47 (11-C, 12-C), 39.22 (2'-C), 55.79 (7-C), 61.78 (5'-C), 70.44 (3'-C), 80.50 (1'-C), 84.82 (4'-C), 102.01 (5-C), 123.37 (8-C), 128.99 (28-C), 129.39 (27-C), 132.93 (26-C), 134.09 (29-C), 148.33 (4-C), 153.82 (2-C), 156.57 (10-C), 157.68 (6-C), 171.30 (25-C) ppm; **ESI(+)-MS** (*m/z*): calc.: 793.22 [$C_{33}H_{48}IN_5O_6Si_2$], found: 794.5 $[M+H]^+$.

1.2.6 Synthesis of 2-*N*-(*N,N*-dimethylaminomethylidenyl)-*N*^{1}-benzoyl-7-deaza-7-iodo-2'-deoxyguanosine 18

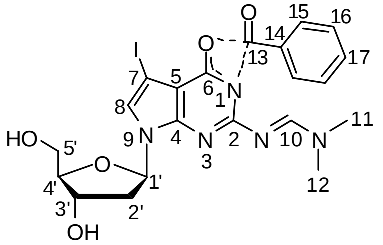

18

$C_{21}H_{22}IN_5O_5$
551.33 g/mol

Compound **32** (1.0 g, 1.26 mmol, 1.0 eq.) was dissolved in 15 mL of THF and 720 µL of Et_3N□ 3HF (approx. 37 % HF) were added. The reaction was stirred at room temperature for 1 h, afterwards the reaction was concentrated under reduced pressure and purified by flash-column chromatography (CH_2Cl_2/MeOH = 98:2 to CH_2Cl_2/MeOH = 95:5) affording 650 mg (97 %) of compound **18** as yellowish solid.

TLC: $\mathbf{R_F}$ = 0.10 (CH_2Cl_2/MeOH = 90:10); **^{1}H-NMR** (300 MHz, DMSO-d_6, 300 K): δ = 2.17 (ddd, 2'-H), 2.42 (m, 2'-H'), 2.61, 3.09 (s, 11-H_3, 12-H_3), 3.56 (m, 5'-H_2), 3.81 (m, 4'-H), 4.35 (m, 3'-H), 4.94 (t, 5'-O*H*), 6.29 (d, 3'-O*H*), 6.48 (dd, 1'-H), 7.40 (s, 8-H), 7.57 (t, 16-H_2), 7.71 (m, 17-H), 7.80 (m, 15-H_2), 8.58 (s, 10-H) ppm; **^{13}C-NMR** (75 MHz, DMSO-d_6, 300 K): δ = 34.39, 40.60 (11-C, 12-C), 39.98 (2'-C), 55.60 (7-C), 61.84 (5'-C), 70.94 (3'-C), 82.48 (1'-C), 87.43 (4'-C), 102.15 (5-C), 124.18 (8-C), 129.23 (16-C), 129.54 (15-C), 133.08 (14-C), 134.25 (17-C), 148.69 (4-C), 153.78 (2-C), 156.50 (10-C), 157.84 (6-C), 171.53 (13-C) ppm; **ESI(+)-MS** (*m/z*): calc.: 551.07 [$C_{21}H_{22}IN_5O_5$] found: 552.0 $[M+H]^+$.

1.2.7 Synthesis of 2-*N*-(*N,N*-dimethylaminomethylidenyl)-N^1-benzoyl-7-deaza-7-iodo-5'-*O*-(4-monomethoxytrityl)-2'-deoxyguanosine 19

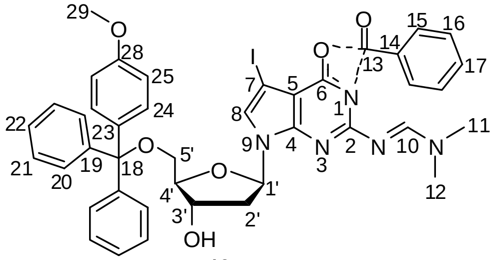

19

$C_{41}H_{38}IN_5O_6$
823.67 g/mol

Compound **18** (600 mg, 1.17 mmol, 1.0 eq.) was dissolved in 10 mL of dry pyridine, 13 mg (0.12 mmol, 0.1 eq.) of *N,N*-dimethylaminopyridine (DMAP) and 500 mg (1.52 mmol, 1.3 eq.) of MMTrCl were added and the reaction stirred at room temperature for 18 h. After 18 h the reaction was quenched with MeOH, concentrated under reduced pressure and purified by flash-column chromatography (CH_2Cl_2/Et_3N = 100:0.1 to $CH_2Cl_2/MeOH/Et_3N$ = 99:1:0.1) affording 1 g (71 %) of compound **19** as yellow foam.

TLC: $\mathbf{R_F}$ = 0.25 (CH_2Cl_2/MeOH = 95:5); **^{1}H-NMR** (400 MHz, DMSO-d_6, 300 K): δ = 2.25 (m, 2'-H_2), 2.61, 3.08 (s, 11-H_3, 12-H_3), 3.11 (m, 5'-H), 3.25 (m, 5'-H'), 3.75 (s, 29-H_3), 3.94 (m, 4'-H), 4.36 (br. s, 3'-H), 5.35 (d, 3'-O*H*), 6.51 (t, 1'-H), 6.89 (br. d, 25-H_2), 7.24-7.42 (m, 8-H, 20-H_4, 21-H_4, 22-H_2, 24-H_2), 7.54 (t, 16-H_2), 7.70 (t, 17-H), 7.79 (d, 15-H_2), 8.58 (s, 10-H) ppm, $J_{16,17}$ = 7.31 Hz; **^{13}C-NMR** (100 MHz, DMSO-d_6, 300 K): δ = 34.27, 40.47 (11-C, 12-C), 39.85 (2'-C), 54.95 (29-C), 55.70 (7-C), 64.29 (5'-C), 70.64 (3'-C), 82.26 (1'-C), 85.76 (4'-C), 102.13 (5-C), 113.09 (25-C), 123.71 (8-C), 126.71, 127.73, 127.80 128.99 (20-C, 21-C, 22-C, 24-C), 129.40 (16-C), 129.84 (15-C), 132.95 (14-C), 134.09 (17-C), 134.83 (23-C), 144.01 (19-C), 144.14 (19-C'), 148.53 (4-C), 153.69 (2-C), 156.37 (10-C), 157.70 (28-C), 158.04 (6-C), 171.36 (13-C), ppm; **ESI(+)-MS** (*m/z*): calc.: 823.19 [$C_{41}H_{38}IN_5O_6$], found: 824.5 $[M+H]^+$; **EA** (%): calc.: C: 59.79, H: 4.65, N: 8.50, found: C: 60.00, H: 4.65, N: 8.50.

1.3 Introduction of the 3'-*O*-(2-cyanoethyl) group and deprotection

1.3.1 General procedure for the introduction of the 2-cyanoethyl group at the 3'-OH position (GP 1)

Derived from the method developed for the 2'-position of RNA by Saneyoshi *et al.*[1] the following procedure was used to introduce the 2-cyanoethyl group to the 3'-OH function of our protected DNA nucleosides **15**, **12** and **19**.

In a well dried Erlenmeyer flask, equipped with a big triangle stirring bar and a septum connected to an argon line, the fully protected nucleoside was dissolved in 20 eq. of freshly distilled acrylonitrile and stirred vigorously for 5 min. To the concentrated solution *t*BuOH (3 mL/mmol to 9 mL/mmol, depending on the solubility of the nucleoside) was added. For the 2'-deoxycytidine derivative **12** DMF was necessary as additional co-solvent (3 mL/mmol). The resulting solution again was stirred vigorously for 5 min before 1 eq. of Cs_2CO_3 was added. The heterogeneous mixture was stirred vigorously for 2 to 3 h at room temperature, then filtrated over silica gel and the solvents removed under reduced pressure. The crude product was either additionally purified by column chromatography or directly used for the subsequent deprotection.

[1] H. Saneyoshi; K. Seio; M. Sekine *J. Org. Chem.* **2005**, *70*, 10453-10460.

1.3.2 Synthesis of 6-*N*-(*N,N*-dimethylaminomethylidenyl)-7-deaza-7-iodo-3'-(2-cyanoethyl)-5'-*O*-bezoyl-2'-deoxyadenosine 33

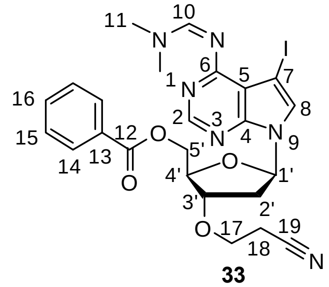

33

$C_{24}H_{25}IN_6O_4$
588.40 g/mol

Following **GP 1** the reaction of 3.0 g (5.6 mmol, 1.0 eq.) of compound **15**, dissolved in 7.5 mL (113.37 mmol, 20 eq.) of acrylonitrile and 20 mL of *t*BuOH, with 1.8 g (5.6 mmol, 1.0 eq.) of Cs_2CO_3 gave after filtration over silica gel (CH_2Cl_2/MeOH = 90:10) 3.2 g (quantitative yield) of compound **33** as yellowish foam.

TLC: $\mathbf{R_F}$ = 0.50 (CH_2Cl_2/MeOH = 95:5); ^{1}**H-NMR** (400 MHz, DMSO-d_6, 300 K): δ = 2.46 (m, 2'-H), 2.76 (m, 2'-H'), 2.84 (t, 18-H_2), 3.18 (s, 11-H_3), 3.22 (s, 11-H_3'), 3.75 (t, 17-H_2), 4.29 (br. q, 4'-H), 4.42-4.55 (m, 3'-H, 5'-H_2), 6.55 (t, 1'-H), 7.55 (t, 15-H_2), 7.67 (m, 8-H, 16-H), 7.99 (d, 14-H_2), 8.31 (s, 2-H), 8.83 (s, 10-H) ppm, $J_{17,18}$ = 5.99 Hz; ^{13}**C-NMR** (100 MHz, DMSO-d_6, 300 K): δ = 18.15 (18-C), 34.76 (11-C), 36.05 (2'-C), 40.25 (11-C'), 54.18 (7-C), 63.65 (17-C), 64.46 (5'-C), 79.08 (3'-C), 80.95 (4'-C), 82.54 (1'-C), 110.05 (5-C), 119.05 (19-C), 128.02 (8-C), 128.74 (15-C), 129.10 (14-C), 129.23 (13-C), 133.35 (16-C), 150.89 (4-C), 151.46 (2-C), 156.03 (10-C), 160.02 (6-C), 165.42 (12-C) ppm; **ESI(+)-MS** (*m/z*): calc.: 588.09 [$C_{24}H_{25}IN_6O_4$], found: 588.9 $[M+H]^+$.

1.3.3 Synthesis of 7-deaza-7-iodo-3'-(2-cyanoethyl)-2'-deoxy-adenosine 16

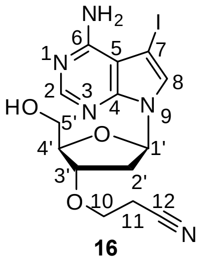

16

$C_{14}H_{16}IN_5O_3$
429.21 g/mol

Compound **33** (3.2 g, 5.6 mmol, 1.0 eq.) was dissolved in 200 mL of saturated methanolic ammonia and stirred at 50 °C for 2 h. After 2 h the mixture was evaporated under reduced pressure and purified by silica gel chromatography (CH_2Cl_2/MeOH = 95:5 to CH_2Cl_2/MeOH = 80:20) affording 2.3 g (96 %) of compound **16** colourless solid.

TLC: $\mathbf{R_F}$ = 0.49 (CH_2Cl_2/MeOH = 95:5); ^{1}**H-NMR** (400 MHz, DMSO-d_6, 300 K): δ = 2.33 (ddd, 2'-H), 2.55 (m, 2'-H'), 2.81 (t, 11-H_2), 3.55 (m, 5'-H), 3.67 (t, 10-H_2), 3.97 (m, 4'-H), 4.24 (m, 3'-H), 5.15 (t, 5'-O*H*), 6.44 (dd, 1'-H), 6.68 (br. s, 6-NH_2), 7.68 (s, 8-H), 8.10 (s, 2-H) ppm, $J_{1',2'}$ = 5.80 Hz, $J_{10,11}$ = 6.15 Hz; ^{13}**C-NMR** (100 MHz, DMSO-d_6, 300 K): δ = 18.15 (11-C), 36.42 (2'-C), 51.89 (7-C), 61.70 (5'-C), 63.28 (10-C), 79.74 (3'-C), 82.79 (1'-C), 84.50 (4'-C), 103.05 (5-C), 119.06 (12-C), 126.57 (8-C), 149.68 (4-C), 151.85 (2-C), 157.04 (6-C) ppm; **ESI(+)-MS** (*m/z*): calc.: 429.03 [$C_{14}H_{16}IN_5O_3$], found: 430.1 $[M+H]^+$; **EA** (%): calc.: C: 39.18, H: 3.76, N: 16.32, found: C: 39.25, H: 3.89, N: 16.07.

1.3.4 Synthesis of 4-*N*-(*N,N*-dimethylaminomethylidenyl)-5-iodo-5'-*O*-benzoyl-3'-*O*-(2-cyanoethyl)-2'-deoxycytidine 34

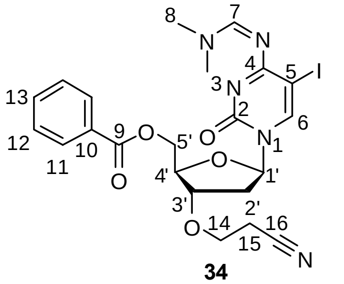

34

$C_{22}H_{24}IN_5O_5$
565.36 g/mol

Following **GP 1** 652 mg (2 mmol, 1.0 eq.) of Cs_2CO_3 were added to a solution of 1.02 g (2 mmol, 1.0 eq.) of compound **12**, dissolved in 2.6 mL (40 mmol, 20 eq.) of acrylonitrile, 12 mL of *t*BuOH and 6 mL of dry DMF. The reaction afforded after filtration over silica gel (CH_2Cl_2/MeOH = 90:10) and additional purification by column chromatography (CH_2Cl_2/MeOH = 98:2 to CH_2Cl_2/MeOH = 90:10) 1.0 g (88 %) of the target compound **34** as yellow foam.

TLC: $\mathbf{R_F}$ = 0.37 (CH_2Cl_2/MeOH = 90:10); ^{1}**H-NMR** (400 MHz, DMSO-d_6, 300 K): δ = 2.59-2.77 (m, 2'-H_2), 2.61 (t, 15-H_2), 3.16 (s, 8-H_3), 3.18 (s, 8-H_3'), 3.72 (t, 14-H_2), 4.18 (m, 4'-H), 4.42 (m, 3'-H), 4.59 (m, 5'-H_2), 6.21 (dd, 1'-H), 7.44 („t“, 12-H_2), 7.56 („t“, 13-H), 8.01 (m, 11-H_2), 8.06 (s, 6-H), 8.68 (s, 7-H) ppm, $J_{13,14}$ = 6.06 Hz; ^{13}**C-NMR** (100 MHz, DMSO-d_6, 300 K): δ = 19.08 (15-C), 35.62 (8-C), 38.81 (2'-C), 41.59 (8-C'), 64.34 (14-C), 64.41 (5'-C), 69.46 (5-C), 80.33 (3'-C), 83.04 (4'-C), 87.20 (1'-C), 117.55 (16-C), 128.86 (12-C), 129.29 (11-C), 129.72 (10-C), 133.64 (13-C), 146.08 (6-C), 155.34 (2-C), 158.91 (7-C), 166.26 (9-C), 168.93 (4-C) ppm; **ESI(+)-MS** (*m/z*): calc.: 565.36 [$C_{22}H_{24}IN_5O_5$], found: 565.8 $[M+H]^+$.

1.3.5 Synthesis of 5-Iodo-3'-*O*-(2-cyanoethyl)-2'-deoxycytidine 13

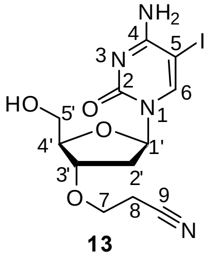

13

$C_{12}H_{15}IN_4O_4$
406.18 g/mol

The fully protected compound **34** (250 mg, 0.44 mmol, 1.0 eq.) was dissolved in 50 mL saturated methanolic ammonia and stirred at room temperature for 2.5 h. The mixture was concentrated in vacuum and the residue purified on a silica gel column (CH_2Cl_2/MeOH = 95:5) affording 147 mg (82 %) of compound **13** as colourless solid.

TLC: $\mathbf{R_F}$ = 0.41 (CH_2Cl_2/MeOH = 90:10); **^{1}H-NMR** (400 MHz, DMSO-d_6, 300 K): δ = 2.06-2.30 (m, 2'-H_2), 2.77 (t, 8-H_2), 3.60 (m, 5'-H_2), 3.62 (t, 7-H_2), 3.94 (m, 4'-H), 4.12 (m, 3'-H), 5.18 (t, 5'-O*H*), 6.05 (dd, 1'-H), 6.62 (br. s, 4-N*H*), 7.82 (br. s, 4-N*H*), 8.24 (s, 6-H) ppm, $J_{7,8}$ = 6.06 Hz; **^{13}C-NMR** (100 MHz, DMSO-d_6, 300 K): δ = 18.34 (8-C), 37.68 (2'-C), 56.81 (5-C), 61.15 (5'-C), 63.62 (7-C), 79.19 (3'-C), 84.74 (4'-C), 85.32 (1'-C), 119.26 (9-C), 147.24 (6-C), 153.83 (2-C), 163.74 (4-C) ppm; **ESI(-)-MS** (*m/z*): calc.: 406.18 [$C_{12}H_{15}IN_4O_4$], found: 404.9 $[M-H]^-$. **EA** (%): calc.: C: 35.48, H: 3.72, N: 13.79, found: C: 35.35, H: 3.93, N: 13.62.

1.3.6 Synthesis of 2-*N*-(*N,N*-dimethylaminomethylidenyl)-*N*^{1}-benzoyl-7-deaza-7-iodo-3'-*O*-(2-cyanoethyl)-5'-*O*-(4-monomethoxytrityl)-2'-deoxyguanosine 20

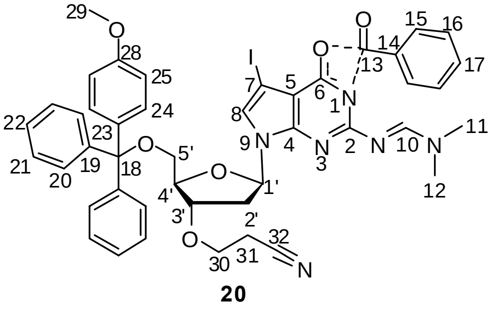

20

$C_{44}H_{41}IN_6O_6$
876.74 g/mol

Following **GP 1** the reaction of 700 mg (0.84 mmol, 1.0 eq.) of compound **19**, dissolved in 1.2 mL (17 mmol, 20 eq.) of acrylonitrile and 7.5 mL of *t*BuOH with 280 mg (0.84 mmol, 1.0 eq.) of

Cs_2CO_3 afforded after filtrated over silica gel (CH_2Cl_2 to CH_2Cl_2/MeOH = 95:5) 650 mg (88 %) of compound **20** as yellow foam.

TLC: $\mathbf{R_F}$ = 0.27 (CH_2Cl_2/MeOH = 95:5); **^{1}H-NMR** (300 MHz, DMSO-d_6, 300 K): δ = 2.43 (ddd, 2'-H), 2.58 (m, 2'-H'), 2.78 (t, 31-H_2), 2.61, 3.06 (s, 11-H_3, 12-H_3), 3.18 (m, 5'-H), 3.29 (m, 5'-H'), 3.65 (m, 30-H_2), 3.75 (s, 29-H_3), 4.02 (m, 4'-H), 4.31 (m, 3'-H), 6.47 (t, 1'-H), 6.89 (d, 25-H_2), 7.23-7.40 (m, 8-H, 20-H_4, 21-H_4, 22-H_2, 24-H_2), 7.41 (t, 16-H_2), 7.67 (t, 17-H), 7.81 (d, 15-H_2), 8.58 (s, 10-H) ppm; **^{13}C-NMR** (75 MHz, DMSO-d_6, 300 K): δ = 18.17 (31-C), 34.25, 40.45 (11-C, 12-C), 36.69 (2'-C), 54.95 (29-C), 55.88 (7-C), 63.45 (30-C), 63.02 (5'-C), 79.26 (3'-C), 82.24 (1'-C), 82.81 (4'-C), 102.15 (5-C), 113.14 (25-C), 123.76 (8-C), 126.77, 127.79, 127.85, 129.01, (20-C, 21-C, 22-C, 24-C), 118.98 (32-C), 129.40 (16-C), 129.85 (15-C), 132.93 (14-C), 134.11 (17-C), 134.76 (23-C), 143.95 (19-C), 144.05 (19-C'), 148.63 (4-C), 153.76 (2-C), 156.45 (10-C), 157.70 (28-C), 158.09 (6-C), 171.34 (13-C) ppm; **ESI(+)-MS** (*m/z*): calc.: 876.21 [$C_{41}H_{38}IN_5O_6$], found: 877.6 $[M+H]^+$; **EA** (%): calc.: C: 60.28, H: 4.71, N: 9.59, found: C: 60.36, H: 4.98, N: 9.62.

1.3.7 Synthesis of 2-*N*-(*N,N*-dimethylaminomethylidenyl)-*N*^{3}-benzoyl-7-deaza-7-iodo-3'-*O*-(2-cyanoethyl)-2'-deoxyguanosine 35

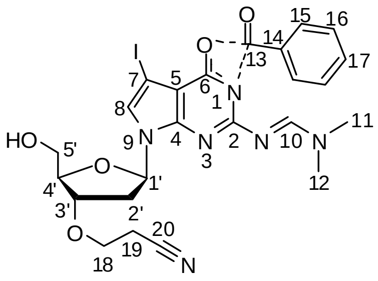

35

$C_{24}H_{25}IN_6O_5$
604.40 g/mol

Compound **20** (650 mg, 0.74 mmol, 1.0 eq.) was dissolved in 5 mL of CH_2Cl_2 and 5 mL of EtOH, 300 mg (1.48 mmol, 2.0 eq.) of PTSA were added and the reaction stirred at room temperature for 1 h. Afterwards the solvents were evaporated under reduced pressure, the crude product dissolved in 100 mL of CH_2Cl_2 and washed with 50 mL of saturated $NaHCO_3$ and saturated NaCl solution. The combined organic layers were dried over sodium sulfate, the solvents evaporated in vacuum and the residue purified by flash-column chromatography (CH_2Cl_2/MeOH = 99:1 to CH_2Cl_2/MeOH = 90:10) affording 300 mg (67 %) of compound **35** as yellow foam.

TLC: $\mathbf{R_F}$ = 0.39 (CH_2Cl_2/MeOH = 95:5); **^{1}H-NMR** (300 MHz, DMSO-d_6, 300 K): δ = 2.42 (ddd, 2'-H), 2'-H' signal hidden below DMSO residual signal, 2.82 (t, 19-H_2), 2.61, 3.08 (s, 11-H_3, 12-

H_3), 3.58 (m, 5'-H_2), 3.69 (t, 18-H_2), 3.97 (m, 4'-H), 4.25 (m, 3'-H), 5.06 (t, 5'-O*H*), 6.45 (dd, 1'-H), 7.44 (s, 8-H), 7.55 (t, 16-H_2), 7.70 (m, 17-H), 7.81 (m, 15-H_2), 8.58 (s, 10-H) ppm; **^{13}C-NMR** (75 MHz, DMSO-d_6, 300 K): δ = 18.22 (19-C), 34.26, 40.47 (11-C, 12-C), 36.90 (2'-C), 55.69 (7-C), 61.69 (5'-C), 63.28 (18-C), 79.70 (3'-C), 82.35 (1'-C), 84.60 (4'-C), 102.05 (5-C), 119.15 (20-C), 123.98 (8-C), 129.06 (16-C), 129.43 (15-C), 132.93 (14-C), 134.16 (17-C), 148.65 (4-C), 153.74 (2-C), 156.49 (10-C), 157.72 (6-C), 171.38 (13-C) ppm; **ESI(+)-MS** (*m/z*): calc.: 604.09 [$C_{24}H_{25}IN_6O_5$], found: 605.2 $[M+H]^+$; **EA** (%): calc.: C: 47.69, H: 4.17, N: 13.90, found: C: 47.41, H: 4.26, N: 13.70.

1.3.8 Synthesis of 7-Deaza-7-iodo-3'-*O*-(2-cyanoethyl)-2'-deoxyguanosine 21

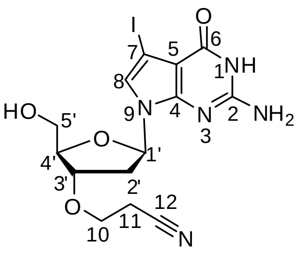

21

$C_{14}H_{16}IN_5O_4$
445.21 g/mol

Compound **35** (250 mg, 0.41 mmol) was dissolved in 10 mL of MeOH and 10 mL of 32 % aqueous NH_3 were added. The reaction was stirred at room temperature for 18 h. The mixture was then evaporated under reduced pressure and purified by flash-column chromatography (CH_2Cl_2/MeOH = 98:2 to CH_2Cl_2/MeOH = 90:10) affording 170 mg (93 %) of compound **21** as yellowish solid.

TLC: $\mathbf{R_F}$ = 0.02 (CH_2Cl_2/MeOH = 90:10); **^{1}H-NMR** (300 MHz, DMSO-d_6, 300 K): δ = 2.24 (ddd, 2'-H), 2.42 (m, 2'-H'), 2.79 (t, 11-H_2), 3.50 (t, 5'-H_2), 3.65 (t, 10-H_2), 3.90 (m, 4'-H), 4.17 (m, 3'-H), 5.01 (t, 5'-O*H*), 6.22 (dd, 1'-H), 6.37 (br. s, 2-N*H₂*), 7.15 (s, 8-H), 10.47 (s, N^1H) ppm, $J_{1',2'}$ = 5.61 Hz, $J_{10,11}$ = 6.03 Hz; **^{13}C-NMR** (75 MHz, DMSO-d_6, 300 K): δ = 18.27 (11-C), 36.43 (2'-C), 55.21 (7-C), 61.82 (5'-C), 63.41 (10-C), 80.01 (3'-C), 82.17 (1'-C), 84.32 (4'-C), 99.90 (5-C), 119.26 (12-C), 121.61 (8-C), 150.67, 152.75 (2-C, 4-C), 158.01 (6-C) ppm; **ESI(+)-MS** (*m/z*): calc.: 445.02 [$C_{14}H_{16}IN_5O_4$], found: 446.0 $[M+H]^+$.

1.4 Synthesis and attachment of the linker

1.4.1 Synthesis of the alkyne modified linker Prop-2-inylcarbamic acid 1-{2-[2-(2-*tert*-butoxycarbonylaminoethoxy)ethoxy]ethoxymethyl}-2-cyanoethyl ester 23

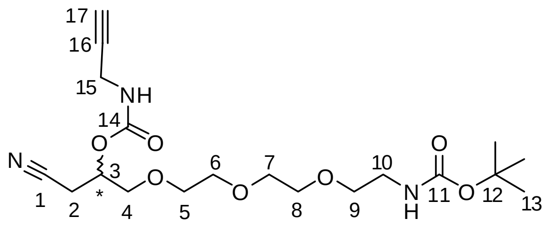

23
$C_{19}H_{31}N_3O_7$
413.47 g/mol

The solution of compound **22** (867 mg, 2.61 mmol, 1.0 eq.) in 50 mL of dry CH_3CN was cooled to 0 °C and 364 mg (2.61 mmol, 1.0 eq.) of water free K_2CO_3 were added. The suspension was stirred for 2 h at 0 °C before 1.41 g (5.22 mmol, 2.0 eq.) of DSC were added and stirring was continued for further 20 h at 0 °C. Afterwards 655 mg (6.52 mmol, 2.5 eq.) of $KHCO_3$ and 540 µL (8.35 mmol, 3.2 eq.) of propargylamine were added and the mixture stirred at 0 °C for 1 h and at room temperature for 4 h. The solvent was removed in vacuum and the residue divided between 50 mL of saturated $NaHCO_3$-solution and 50 mL of CH_2Cl_2. The organic layer was washed with 50 mL of saturated NH_4Cl-solution and the combined aqueous layers extracted again two times with 50 mL of CH_2Cl_2. The combined organic layers were dried over Na_2SO_4 and the solvent was removed in vacuum after filtration. The oily residue was purified by flash-column chromatography (CH_2Cl_2/MeOH = 99:1 to CH_2Cl_2/MeOH = 98:2) affording 728 mg (68 %) of compound **23** as colourless oil.

TLC: $\mathbf{R_F}$ = 0.25 (CH_2Cl_2/MeOH = 95:5); **^{1}H-NMR** (250 MHz, DMSO-d_6, 300 K): δ = 1.37 (s, 13-H_9), 2.77-2.95 (m, 2-H_2), 3.06 (q, 10-H_2), 3.10 (t, 17-H), 3.37 (t, 9-H_2), 3.47-3.62 (m, 4-H_2, 5-H_2, 6-H_2, 7-H_2, 8-H_2), 3.78 (dd, 15-H_2), 4.95 („quintett“, 3-H), 6.74 (t, $N^{Boc}H$), 7.87 (t, N*H*) ppm, $J_{9,10}$ = 5.9 Hz, $J_{15,NH'}$ = 5.8 Hz, $J_{15,17}$ = 2.5 Hz; **^{13}C-NMR** (62.5 MHz, DMSO-d_6, 300 K): δ = 19.71 (2-C), 28.22 (13-C), 29.82 (15-C), 39.68 (10-C), 67.86 (3-C), 69.17 (9-C), 69.50, 69.61, 69.74, 70.20, 70.31 (4-C, 5-C, 6-C, 7-C, 8-C), 73.14 (17-C), 77.60 (12-C), 81.06 (16-C), 117.67 (1-C), 154.87 (14-C), 155.57 (11-C) ppm; **ESI(+)-MS** *m/z*: calc.: 413.47 [$C_{19}H_{31}N_3O_7$], found: 436.1 $[M+Na]^+$, 414.2 $[M+H]^+$, 314.0 $[M-Boc+H]^+$; **EA** (%): calc.: C: 55.19, H: 7.56, N: 10.16, found: C: 55.09, H: 7.59, N: 10.04.

1.4.2 Synthesis of 7-Deaza-7-(prop-2-ynyl)carbamic acid-1-{2-[2-(2-*tert*-butoxycarbonylaminoethoxy)ethoxy]ethoxymethyl}-2-cyanoethyl ester-3'-*O*-(2-cyanoethyl)-2'desoxyadenosine 26

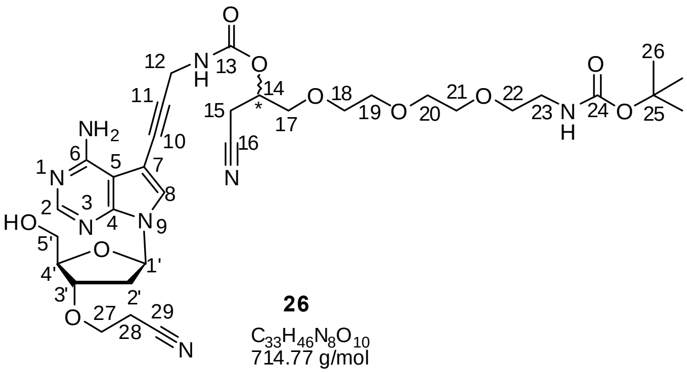

26

$C_{33}H_{46}N_8O_{10}$
714.77 g/mol

Following **GP 2** (see experimental section in the article) 150 mg (0.35 mmol, 1.0 eq.) of 7-deaza-7-iodo-3'-*O*-(2-cyanoethyl)-2'-deoxyadenosine **16** and 217 mg (0.52 mmol, 1.5 eq.) of alkyne **23** gave, after flash-column chromatography (CH_2Cl_2/MeOH = 99:1 to CH_2Cl_2/MeOH = 95:5), 168 mg (67 %) of compound **26** as slightly yellow foam.

TLC: $\mathbf{R_F}$ = 0.38 (CH_2Cl_2/MeOH = 90:10); **^{1}H-NMR** (400 MHz, DMSO-d_6, 300 K): δ = 1.36 (s, 26-H_9), 2.33-2.41 (m, 2'-H), 2.52-2.60 (m, 2'-H'), 2.80 (t, 28-H_2), 2.86 (dd, 15-H), 2.92 (dd, 15-H'), 3.05 (q, 23-H_2), 3.35 (t, 22-H_2), 3.44-3.62 (m, 5'-H_2, 17-H_2, 18-H_2, 19-H_2, 20-H_2, 21-H_2), 3.65-3.71 (m, 27-H_2), 3.96-3.99 (m, 4'-H), 4.07 (d, 12-H_2), 4.23-4.27 (m, 3'-H), 4.99 (quintett, 14-H), 5.17 (t, 5'-O*H*), 6.42 (dd, 1'-H), 6.69-6.75 (m, 23-N*H*), 6.00-7.45 (br. s, 6-NH_2), 7.73 (s, 8-H), 8.03 (t, 12-N*H*), 8.10 (s, 2-H) ppm, $J_{12,12\text{-N}H}$ = 5.2 Hz, $J_{15,15'}$ = 17.1 Hz; **^{13}C-NMR** (100 MHz, DMSO-d_6, 300 K): δ = 18.26 (28-C), 19.73 (15-C), 28.21 (26-C), 30.97 (12-C), 36.58 (2'-C), 39.67 (23-C), 61.83 (5'-C), 63.40 (27-C), 67.93 (14-C), 69.15 (22-C), 69.46, 69.59, 69.71, 70.20, 70.29 (17-C, 18-C, 19-C, 20-C, 21-C), 75.42 (10-C), 77.59 (25-C), 79.90 (3'-C), 83.19 (1'-C), 84.71 (4'-C), 88.81 (11-C), 94.70 (7-C), 102.28 (5-C), 117.68 (16-C), 119.21 (29-C), 125.97 (8-C), 149.28 (4-C), 152.71 (2-C), 155.14 (13-C), 155.56 (24-C), 157.51 (6-C) ppm; **ESI(+)-MS** (*m/z*): calc.: 714.77 [$C_{33}H_{46}N_8O_{10}$], found: 715.8 $[M+H]^+$; **EA** (%): calc.: C: 55.45, H: 6.49, N: 15.68, found: C: 55.41, H: 6.59, N: 15.42.

1.4.3 Synthesis of 5-(Prop-2-ynyl)carbamic acid-1-{2-[2-(2-*tert*-butoxycarbonylaminoethoxy)ethoxy]ethoxymethyl}-2-cyano-ethyl ester-3'-*O*-(2-cyanoethyl)-2'-deoxycytidine 25

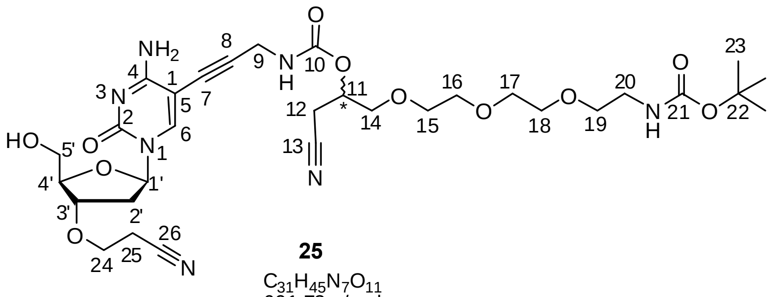

25

$C_{31}H_{45}N_7O_{11}$
691.73 g/mol

Following **GP 2** (see experimental section in the article) the reaction of 77 mg (0.19 mmol, 1.0 eq.) 5-iodo-3'-*O*-(2-cyanoethyl)-2'-deoxycytidine **13** and 157 mg (0.38 mmol, 2.0 eq.) of alkyne **23** gave, after flash-column chromatography (CH_2Cl_2/MeOH = 98:2 to CH_2Cl_2/MeOH = 92:8), 102 mg (78 %) of compound **25** as yellowish foam.

TLC: $\mathbf{R_F}$ = 0.11 (CH_2Cl_2/MeOH = 90:10); **^{1}H-NMR** (400 MHz, DMSO-d_6, 300 K): δ = 1.37 (s, 23-H_9), 2.03-2.12 (m, 2'-H), 2.25-2.32 (m, 2'-H'), 2.77 (t, 25-H_2), 2.84 (dd, 12-H), 2.91 (dd, 12-H'), 3.05 (q, 20-H_2), 3.36 (t, 19-H_2), 3.47-3.65 (m, 5'-H_2, 24-H_2, 14-H_2, 15-H_2, 16-H_2, 17-H_2, 18-H_2), 3.93-3.97 (m, 4'-H), 4.06 (d, 9-H_2), 4.10-4.15 (m, 3'-H), 4.93-4.99 (m, 11-H), 5.11-5.16 (m, 5'-O*H*), 6.08 (t, 1'-H), 6.70-6.76 (m, 20-N*H*), 6.82 (s, 4-N*H*), 7.80 (s, 4-N*H*'), 7.88-7.94 (m, 9-N*H*), 8.09 (s, 6-H) ppm, $J_{12,12'}$ = 17.3 Hz, $J_{19,20}$ = 5.9 Hz; **^{13}C-NMR** (100 MHz, DMSO-d_6, 300 K): δ = 18.22 (25-C), 19.73 (12-C), 28.22 (23-C), 31.08 (9-C), 37.58 (2'-C), 39.67 (20-C), 61.20 (5'-C), 63.53 (24-C), 67.93 (11-C), 69.16 (19-C), 69.48, 69.60, 69.73, 70.20, 70.30 (14-C, 15-C, 16-C, 17-C, 18-C), 74.59 (7-C), 77.58 (22-C), 79.17 (3'-C), 84.77 (4'-C), 85.31 (1'-C), 89.46 (5-C), 91.64 (8-C), 117.68 (13-C), 119.15 (26-C), 143.89 (6-C), 153.35 (2-C), 154.82 (10-C), 155.56 (21-C), 164.30 (4-C) ppm; **ESI(+)-MS** (*m/z*): calc.: 691.73 [$C_{31}H_{45}N_7O_{11}$], found: 692.3 $[M+H]^+$, 714.4 $[M+Na]^+$; **EA** (%): calc.: C: 53.83, H: 6.56, N: 14.17, found: C: 53.57, H: 6.62, N: 13.98.

1.4.4 Synthesis of 7-Deaza-7-(prop-2-ynyl)carbamic acid-1-{2-[2-(2-*tert*-butoxycarbonylaminoethoxy)ethoxy]ethoxymethyl}-2-cyanoethyl ester-3'-*O*-(2-cyanoethyl)-2'-desoxyguanosine 27

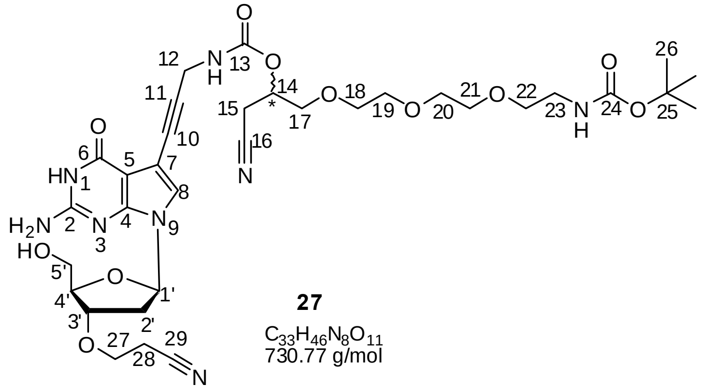

27
$C_{33}H_{46}N_8O_{11}$
730.77 g/mol

Following **GP 2** (see experimental section in the article) the reaction of 187 mg (0.42 mmol, 1.0 eq.) of 7-deaza-7-iodo-3'-*O*-(2-cyanoethyl)-2'-deoxyguanosine **21** and 260 mg (0.63 mmol, 1.5 eq.) of alkyne **23** yielded, after flash-column chromatography (CH_2Cl_2 to CH_2Cl_2/MeOH = 90:10), 230 mg (75 %) of compound **27** as slightly yellow foam.

TLC: $\mathbf{R_F}$ = 0.36 (CH_2Cl_2/MeOH = 90:10); ^{1}**H-NMR** (300 MHz, DMSO-d_6, 300 K): δ = 1.37 (s, 26-H_9), 2.26 (ddd, 2'-H), 2.36-2.47 (m, 2'-H'), 2.79 (t, 28-H_2), 2.82-2.97 (m, 15-H_2), 3.05 (q, 23-H_2), 3.36 (t, 22-H_2), 3.46-3.62 (m, 5'-H_2, 17-H_2, 18-H_2, 19-H_2, 20-H_2, 21-H_2), 3.65 (t, 27-H_2), 3.87-3.94 (m, 4'-H), 4.02 (d, 12-H_2), 4.14-4.20 (m, 3'-H), 4.91-5.05 (m, 14-H, 5'-O*H*), 6.22 (dd, 1'-H), 6.35 (br. s, 2-NH_2), 6.73 (t, 23-N*H*), 7.27 (s, 8-H), 7.917 (t, 12-N*H*), 10.50 (s, N^1H) ppm, $J_{12,12\text{-}NH}$ = 5.5 Hz, $J_{27,28}$ = 6.1 Hz; ^{13}**C-NMR** (75 MHz, DMSO-d_6, 300 K): δ = 18.23 (28-C), 19.70 (15-C), 28.18 (26-C), 30.62 (12-C), 36.50 (2'-C), 39.65 (23-C), 61.79 (5'-C), 63.40 (27-C), 67.78 (14-C), 69.13 (22-C), 69.45, 69.56, 69.69, 70.18, 70.28 (17-C, 18-C, 19-C, 20-C, 21-C), 76.24 (10-C), 77.55 (25-C), 79.99 (3'-C), 82.23 (1'-C), 84.37 (4'-C), 86.37 (11-C), 98.46 (7-C), 99.34 (5-C), 117.64 (16-C), 119.20 (29-C), 122.02 (8-C), 150.34 (4-C), 153.18, 157.72 (2-C, 6-C), 154.83 (13-C), 155.52 (24-C) ppm; **ESI(+)-MS** (*m/z*): calc.: 730.77 [$C_{33}H_{46}N_8O_{11}$], found: 731.6 $[M+H]^+$, 753.6 $[M+Na]^+$, 769.6 $[M+K]^+$; **EA** (%): calc.: C: 54.24, H: 6.34, N: 15.33, found: C: 54.00, H: 6.31, N: 15.21.

1.5 Triphosphate synthesis and attachment of the dye

1.5.1 Synthesis of 7-Deaza-7-(prop-2-ynyl)carbamic acid-1-{2-[2-(2-*tert*-butoxycarbonylaminoethoxy)ethoxy]ethoxymethyl}-2-cyanoethyl ester-3'-*O*-(2-cyanoethyl)-2'-deoxyadenosine 5'-triphosphate 30

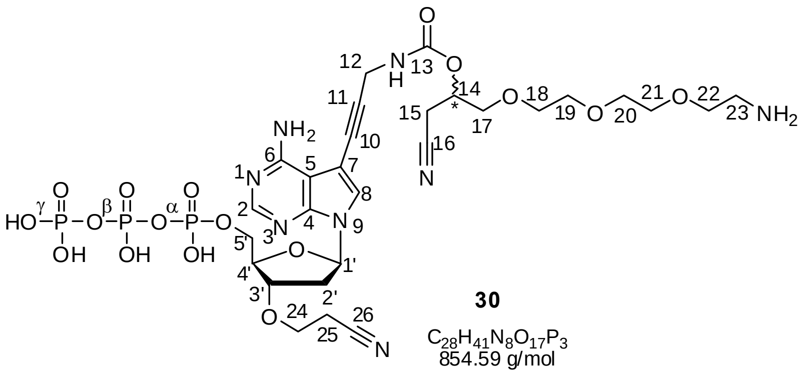

30

$C_{28}H_{41}N_8O_{17}P_3$
854.59 g/mol

Following **GP 3** (see experimental section in the article) 161 mg (0.23 mmol, 1.0 eq.) of the *N*-Boc protected nucleoside **26** gave 36 mg (19 %) of triphosphate **30** as yellowish oil. The yield was calculated from the ^{1}H-NMR spectrum as the product contained rests of triethylammonium acetate after HPLC purification.

RP-TLC: $\mathbf{R_F}$ = 0.71 (H_2O/CH_3CN = 80:20); **IE-FPLC**: elution concentration: 0.34 M TEAB-buffer (45 % B); **RP-HPLC**: retention time: 16.65 min; ^{1}**H-NMR** (400 MHz, D_2O, 300 K): δ = 2.63 (m, 2'-H_2), 2.87 (t, 25-H_2), 2.92-3.04 (m, 15-H_2), 3.12-3.18 (m, 23-H_2), 3.49-3.81 (m, 17-H_2, 18-H_2, 19-H_2, 20-H_2, 21-H_2, 22-H_2), 3.90 (t, 24-H_2), 4.09-4.27 (m, 5'-H_2, 12-H_2), 4.41 (br. s, 4'-H), 4.55 (br. s, 3'-H), 5.20-5.29 (m, 14-H), 6.54 (t, 1'-H), 7.72 (s, 8-H), 8.18 (s, 2-H) ppm, $J_{23,24}$ = 5.8 Hz; ^{13}**C-NMR** (62.5 MHz, D_2O, 300 K): δ = 19.01 (25-C), 20.26 (15-C), 31.66 (9-C), 36.85/37.05 (2'-C), 39.44/39.49 (23-C), 64.46 (24-C), 66.52 (d, 5'-C), 66.90/66.94, 69.82, 69.89, 70.02, 70.72/70.78, 71.24 (17-C, 18-C, 19-C, 20-C, 21-C, 22-C), 69.12/69.23 (14-C), 75.43/75.48 (10-C), 80.77/80.88 (3'-C), 83.65 (m, 4'-C), 83.82 (1'-C), 89.71 (11-C), 96.63/96.71 (7-C), 103.31 (5-C), 118.76 (16-C), 120.55 (26-C), 126.52/126.70 (8-C), 149.09/149.19 (4-C), 152.78/152.85 (2-C), 157.42 (6-C), 157.59 (13-C) ppm, $J_{5',\alpha\text{-}P}$ = 5.81 Hz; ^{31}**P-NMR** (121 MHz, D_2O, 300 K): δ = -10.62 (d, γ-P), -11.32 (d, α-P), -23.04 (t, β-P) ppm; **ESI(-)-MS** (*m/z*) calc.: 854.59 [$C_{28}H_{41}N_8O_{17}P_3$], found: 853.6 $[M-H]^-$.

1.5.2 Synthesis of 5-(Prop-2-ynyl)carbamic acid-1-{2-[2-(2-*tert*-butoxycarbonylaminoethoxy)ethoxy]ethoxymethyl}-2-cyano-ethyl ester-3'-*O*-(2-cyanoethyl)-2'-deoxycytidine 5'-triphosphate 29

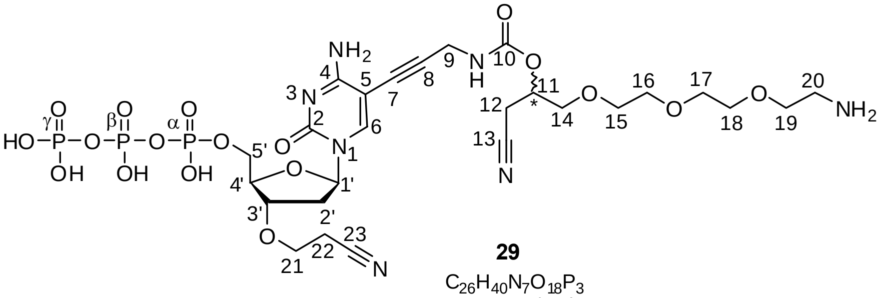

29
$C_{26}H_{40}N_7O_{18}P_3$
831.55 g/mol

Following **GP 3** (see experimental section in the article) 236 mg (0.34 mmol, 1.0 eq.) of the *N*-Boc protected nucleoside **25** yielded 23 mg (8 %) of triphosphate **29** as colourless oil. The yield was calculated from the ^{1}H-NMR spectrum as the product contained rests of triethylammonium acetate after HPLC purification.

RP-TLC: $\mathbf{R_F}$ = 0.77 (H_2O/CH_3CN = 80:20); **IE-FPLC**: elution concentration: 0.31 M TEAB-buffer (35 % B); **RP-HPLC**: retention time: 15.10 min; **^{1}H-NMR** (300 MHz, D_2O, 300 K): δ = 2.24-2.34 (m, 2'-H), 2.58-2.67 (m, 2'-H'), 2.86 (t, 22-H_2), 2.92-2.99 (m, 12-H_2), 3.24 (m, 20-H_2), 3.69-3.79 (m, 14-H_2, 15-H_2, 16-H_2, 17-H_2, 18-H_2, 19-H_2), 3.88 (t, 21-H_2), 4.18-4.30 (m, 5'-H_2, 9-H_2), 4.39-4.45 (m, 4'-H), 4.46-4.52 (m, 3'-H), 5.20-5-29 (m, 11-H), 6.26-6.34 (m, 1'-H), 8.22/8.23 (s, 6-H) ppm, $J_{21,22}$ = 5.9 Hz; **^{13}C-NMR** (75 MHz, D_2O, 300 K): δ = 18.90 (22-C), 20.18/20.21 (12-C), 31.54 (9-C), 37.82/37.91 (2'-C), 39.65 (20-C), 64.44/64.45 (21-C), 66.28 (d, 5'-C), 66.93, 70.05, 70.13, 70.18, 70.80, 71.22 (14-C, 15-C, 16-C, 17-C, 18-C, 19-C), 69.31 (11-C), 73.47 (7-C), 80.42/80.51 (3'-C), 84.29 (m, 4'-C), 87.07/87.22 (1'-C), 92.78, 93.12 (5-C, 8-C), 118.79 (13-C), 120.44 (23-C), 145.57 (6-C), 156.09/156.13 (2-C), 157.39 (10-C), 165.31 (4-C) ppm, $J_{5',\alpha\text{-P}}$ = 6.49 Hz; **^{31}P-NMR** (121 MHz, D_2O, 300 K): δ = -10.85 (d, γ-P), -11.53 (d, α-P), -23.21 (t, β-P) ppm, $J_{\alpha,\beta}$ = 20.0 Hz, $J_{\gamma,\beta}$ = 20.0 Hz; **ESI(-)-MS** (*m/z*): calc.: 831.55 [$C_{26}H_{40}N_7O_{18}P_3$], found: 830.5 [M-H]$^-$.

1.5.3 Synthesis of 7-Deaza-7-(prop-2-ynyl)carbamic acid-1-{2-[2-(2-*tert*-butoxycarbonylaminoethoxy)ethoxy]ethoxymethyl}-2-cyanoethyl ester-3'-*O*-(2-cyanoethyl)-2'-deoxyguanosine 5'-triphosphate 31

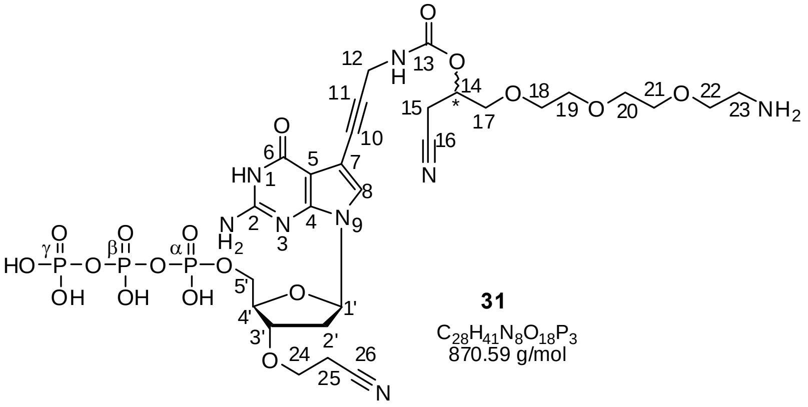

31
$C_{28}H_{41}N_8O_{18}P_3$
870.59 g/mol

Following **GP 3** (see experimental section in the article) 72 mg (0.1 mmol, 1.0 eq.) of the *N*-Boc protected nucleoside **27** gave 22 mg (26 %) of triphosphate **31** as colourless oil. The yield was calculated from the ^{1}H-NMR spectrum as the product contained rests of triethylammonium acetate after HPLC purification.

RP-TLC: $\mathbf{R_F}$ = 0.64 (H_2O/CH_3CN = 90:10); **IE-FPLC**: elution concentration: 0.35 M TEAB-buffer (40 % B); **RP-HPLC**: retention time: 15.13 min; ^{1}**H-NMR** (300 MHz, D_2O, 300 K): δ = 2.54 (ddd, 2'-H), 2.58-2.70 (m, 2'-H'), 2.85 (t, 25-H_2), 2.89-2.98 (m, 15-H_2), 3.17 (23-H_2, from 2D-NMR, hidden below Et_3N signal), 3.55-3.80 (m, 17-H_2, 18-H_2, 19-H_2, 20-H_2, 21-H_2, 22-H_2), 3.89 (dt, 24-H_2), 4.06-4.28 (m, 5'-H_2, 12-H_2), 4.36 (m, 4'-H), 4.53 (br. d, 3'-H), 5.21 (m, 14-H), 6.38 (dd, 1'-H), 7.40 (s, 8-H) ppm, $J_{1',2'}$ = 5.8 Hz; ^{13}**C-NMR** (150 MHz, D_2O, 300 K): δ = 18.97 (25-C), 20.16 (15-C), 31.64 (12-C), 36.69 (2'-C), 39.56 (23-C), 64.36 (24-C), 66.50 (d, 5'-C), 66.92, 69.96, 70.10 (2C), 70.78, 71.25 (17-C, 18-C, 19-C, 20-C, 21-C, 22-C), 69.13 (14-C), 76.09 (10-C), 80.89 (3'-C), 83.63 (d, 4'-C), 83.92/83.98 (1'-C), 87.74, 99.22, 100.61 (5-C, 7-C, 11-C), 118.72 (16-C), 120.48 (26-C), 124.44/124.63 (8-C), 151.49 (4-C), 153.84, 161.23 (2-C, 6-C), 157.38/157.42 (13-C) ppm, $J_{4'\text{-}\alpha\text{-}P}$ = 9.22 Hz, $J_{5'\text{-}\alpha\text{-}P}$ = 5.63 Hz; ^{31}**P-NMR** (121 MHz, D_2O, 300 K): δ = -10.70 (d, γ-P), -11.39 (d, α-P), -23.14 (t, β-P) ppm; **ESI(-)-MS** (*m/z*): calc.: 870.59 [$C_{26}H_{40}N_7O_{18}P_3$], found: 870.0 $[M-H]^-$.

1.5.4 General procedure for the purification of labelled triphosphates (GP 4)

Two chromatographic purification steps were carried out on the concentrated, crude reaction mixtures of compounds **2**, **3**, **4**, and **5**. The first step was a purification by RP-FPLC at 4 °C as described in the materials and methods. The following gradient was used for all four compounds:
RP-FPLC: flow rate: 3 mL/min, gradient: 0 mL: 0 % B→60 mL: 1 % B→120 mL: 5 % B→160 mL: 10 % B→220 mL: 20 % B→250 mL: 50 % B→280 mL: 100 % B→310 mL: 100 % B,
The second step was a purification by RP-HPLC as described in the materials and methods. The following gradients were used.
For compounds **3** and **4**:
RP-HPLC (A, C): flow rate: 6 mL/min, gradient: 0 min.: 10 % A, 90 % B, 0 % C→15 min.: 10 % A, 60 % B, 30 % C→16 min.: 0 % A, 0 % B, 100 % C→22 min.: 0 % A, 0 % B, 100 % C→23 min.: 10 % A, 90 % B, 0 % C→30 min.: 10 % A, 90 % B, 0 % C
For compounds **2** and **5**:
RP-HPLC (T, G): flow rate: 6 mL/min, gradient: 0 min.: 10 % A, 90 % B, 0 % C□18 min.: 10 % A, 54 % B, 36 % C→18.5 min.: 0 % A, 0 % B, 100 % C→26.5 min.: 0 % A, 0 % B, 100 % C→27 min.: 10 % A, 90 % B, 0 % C→30 min.: 10 % A, 90 % B, 0 % C.

1.5.5 Synthesis of 7-Deaza-7-(prop-2-ynyl)carbamic acid-1-{2-[2-(2-5- and 6-carboxy-X-rhodamineamidoethoxy)ethoxy]ethoxy-methyl}-2-cyanoethyl ester-3'-*O*-(2-cyanoethyl)-2'-desoxy-adenosine 5'-triphosphate 3

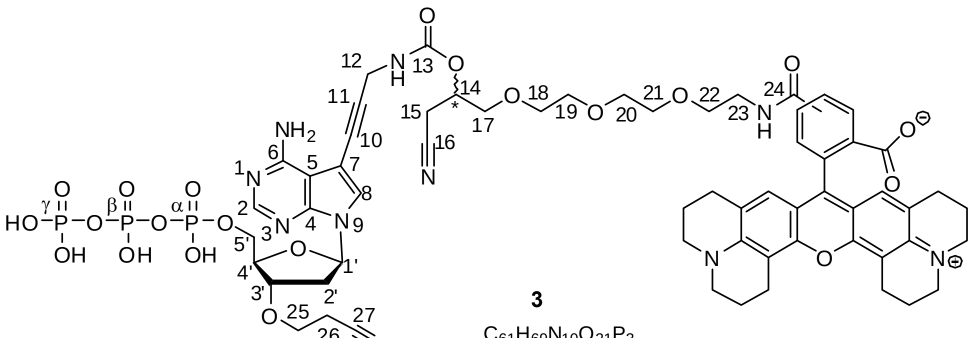

3
$C_{61}H_{69}N_{10}O_{21}P_3$
1371.18 g/mol

To a solution of 20 mg (23 µmol, 1.0 eq.) of the triphosphate **30** in 500 µL of dry DMF and 12 µL (70 µmol, 3 eq.) of *N*-ethyldiisopropylamine a solution of 5- and 6-carboxy-X-rhodamine-(*N*-hydroxyssuccinimidyl)ester (30 mg, 50 µmol, 2.0 eq.) in 500 µL of dry DMF was added in two portions. One half directly and the other half after 1 h at room temperature. The mixture was left stirring for 20 h at room temperature in the dark. After evaporation of the solvent the crude product

was dissolved in 3 mL of Millipore water and filtered through a 45 µm syringe filter before purification by RP-FPLC and preparative RP-HPLC (see **GP 4**). The triethylammonium salt of the labelled triphosphate **3** was obtained as dark red solid (5 mg, 16 %) as a mixture of the two regioisomers, each consisting of two diastereomers.

RP-TLC: $\mathbf{R_F}$ = 0.52 (H_2O/CH_3CN = 50:50); **RP-FPLC**: elution concentration: 13 % B – 100 % B; **RP-HPLC**: retention time: 18.87 min (UV detection), 19.06 min (fluorescence detection); **UV absorption**: 594 nm, 552 nm, 374 nm, 271 nm; **fluorescence**: excitation 592 nm, emission 610 nm; **^{1}H-NMR** (400 MHz, D_2O, 300 K): δ = 1.55-2.13 (9H), 2.22-3.11 (20H), 3.33-4.32 (22H), 4.38-4.58 (1H) (each signal m, 2'-H_2, 3'-H, 4'-H, 5'-H_2, 12-H_2, 15-H_2, 17-H_2, 18-H_2, 19-H_2, 20-H_2, 21-H_2, 22-H_2, 23-H_2, 25-H_2, 26-H_2, 5- and 6-carboxy-X-rhodamine-$H_{24,aliphatic}$), 4.96-5.21 (m, 14-H), 5.92-8.81 (m, 1'-H, 2-H, 8-H, 5- and 6-carboxy-X-rhodamine-$H_{5,aromatic}$) ppm; **^{31}P-NMR** (121 MHz, D_2O, 300 K): δ = -10.73 (d, γ-P), -11.50 (d, α-P), -22.70 - -23.45 (m, β-P) ppm; **ESI(-)-MS** (*m/z*): calc.: 1371.18 [$C_{61}H_{69}N_{10}O_{21}P_3$], found: 684.7 $[M-2H]^{2-}$, 1370.4 $[M-H]^-$.

1.5.6 Synthesis of 5-(Prop-2-ynyl)carbamic acid-1-{2-[2-(2-Cy 3.0-amidoethoxy)ethoxy]ethoxymethyl}-2-cyanoethyl ester-3'-*O*-(2-cyanoethyl)2'-deoxycytidine 5'-triphosphate 4

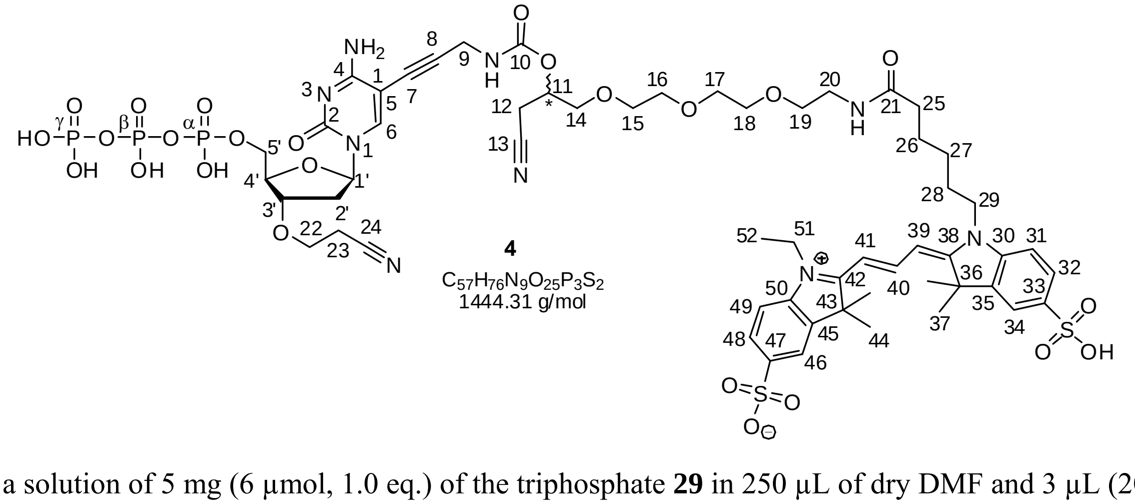

To a solution of 5 mg (6 µmol, 1.0 eq.) of the triphosphate **29** in 250 µL of dry DMF and 3 µL (20 µmol, 3 eq.) of *N*-ethyldiisopropylamine a solution of Cy 3.0-*N*-hydroxysuccinimidyl ester monopotassium salt (5 mg, 6 µmol, 1.0 eq.) in 250 µL of dry DMF was added. The mixture was left stirring for 20 h at room temperature in the dark. After evaporation of the solvent the crude product was dissolved in 3 mL of Millipore water and filtered through a 45 µm syringe filter before purification by RP-FPLC and preparative RP-HPLC (see **GP 4**). The triethylammonium salt of the labelled triphosphate **4** was obtained as pink solid (3 mg, 36 %) as a mixture of two diastereomers.

RP-TLC: $\mathbf{R_F}$ = 0.66 (H_2O/CH_3CN = 80:20); **RP-FPLC**: elution concentration: 5 % B – 10 % B; **RP-HPLC**: retention time: 17.74 min (UV detection), 17.56 min (fluorescence detection); **UV absorption**: 550 nm, 519 nm, 292 nm; **fluorescence**: excitation 550 nm, emission 563 nm; ^{1}**H-NMR** (400 MHz, D_2O, 300 K): δ = 1.36 (aus 2D, 25/26/27/28-H_2, 52-H_3), 1.55-1.65 (m, 25/26/27/28-H_2), 1.701 (s, 37-H_6, 44-H_6), 1.812 (br. s, 25/26/27/28-H_2) 2.00-2.12 (m, 2'-H), 2.20 (m, 25/26/27/28-H_2), 2.37-2.47 (m, 2'-H'), 2.67 (t, 23-H_2), 2.87 (br. s, 12-H_2), 3.20-3.27 (m, 20-H_2), 3.39-3-68 (m, 14-H_2, 15-H_2, 16-H_2, 17-H_2, 18-H_2, 19-H_2), 3.70 (t, 22-H_2), 4.12 (m, 5'-H_2, 9-H_2, 29-H_2, 51-H_2), 4.22 (br. s, 4'-H), 4.34 (d, 3'-H), 5.12 (m, 11-H), 6.04 (“t”, 1'-H), 6.33 (t, 39-H, 41-H), 7.36 (dd, 31/32/48/49-H_2), 7.79-7.85 (31/32/48/49-H_2), 7.87 (s, 32-H, 46-H), 8.05 (s, 6-H), 8.46 (t, 40-H) ppm, $J_{22,23}$ = 6.0 Hz, $J_{39,40}$ = 13.4 Hz, $J_{40,41}$ = 13.4 Hz; ^{31}**P-NMR** (121 MHz, D_2O, 300 K): δ = -11.28 (br. s, α-P, γ-P), -23.61 (br. s, β-P) ppm; **ESI(-)-MS** (*m/z*): calc.: 1444.31 [$C_{57}H_{76}N_9O_{25}P_3S_2$], found: 480.4 $[M-3H]^{3-}$, 721.0 $[M-2H]^{2-}$, 681.0 $[M-PO_3-2H]^{2-}$, 453.5 $[M-PO_3-3H]^{2-}$, 732.1 $[M+Na-2H]^{2-}$.

1.5.7 Synthesis of 7-Deaza-7-(prop-2-ynyl)carbamic acid-1-{2-[2-(2-Cy 5.0-amidoethoxy)ethoxy]ethoxymethyl}-2-cyanoethyl ester-3'-*O*-(2-cyanoethyl)-2'-desoxyguanosine 5'-triphosphate 5

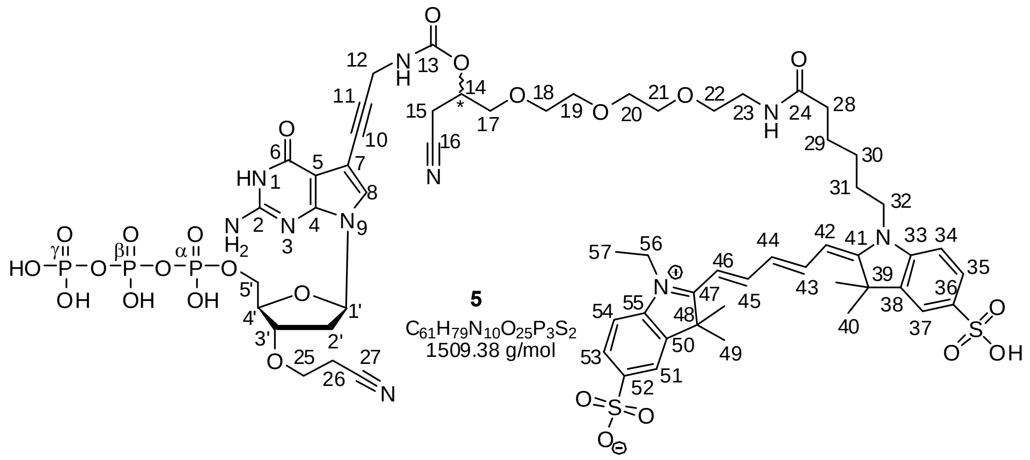

To a solution of 5 mg (6 µmol, 1.0 eq.) of the triphosphate **31** in 250 µL of dry DMF and 4 µL (23 µmol, 4 eq.) of *N*-ethyldiisopropylamine a solution of Cy 5.0-*N*-hydroxysuccinimidyl ester monopotassium salt (5 mg, 6 µmol, 1.0 eq.) in 250 µL of dry DMF was added. The mixture was left stirring for 20 h at room temperature in the dark. After evaporation of the solvent the crude product was dissolved in 3 mL of Millipore water and filtered through a 0.45 µm syringe filter before purification by RP-FPLC and preparative RP-HPLC (see **GP 4**). The triethylammonium salt of the labelled triphosphate **5** was obtained as dark blue solid (1.5 mg, 17 %) as a mixture of two diastereomers.

RP-TLC: $\mathbf{R_F}$ = 0.38 (H_2O/CH_3CN = 90:10); **RP-FPLC**: elution concentration: 5 % B – 10 % B; **RP-HPLC**: retention time: 18.55 min. (UV detection), 18.68 min (fluorescence detection); **UV absorption**: 648 nm, 605 nm, 319 nm, 245 nm; **fluorescence**: excitation 646 nm, emission 666 nm; ^{1}**H-NMR** (400 MHz, D_2O, 300 K): δ = 1.37 (from 2D, 28,29,30,31-H_2, 57-H_3), 1.61-1.69 (m, 28,29,30,31-H_2), 1.73, 1.74 (s, 40-H_6, 49-H_6), 1.78-1.86 (m, 28,29,30,31-H_2), 2.27 (t, 28,29,30,31-H_2), 2.44 (dd, 2'-H), 2.60-2.72 (m, 2'-H'), 2.78-2.90 (m, 15-H_2, 29-H_2), 3.32 (m, 23-H_2), 3.54-3.70 (m, 17-H_2, 18-H_2, 19-H_2, 20-H_2, 21-H_2, 22-H_2) 3.76-3.89 (m, 25-H2), 3.93-4.28 (m, 5'-H_2, 12-H_2, 32-H_2, 56-H_2), 4.35 (br. s, 4'-H), 4.55 (d, 3'-H), 5.04 (m, 17-H), 6.18-6.45 (m, 1'-H, 42,43,44,45,46-H_2), 6.58 (t, 42,43,44,45,46-H_1), 7.30, 7.38 (d, 34-H, 54-H), 7.74-8.29 (8-H, 35-H, 37-H, 42,43,44,45,46-H_2, 51-H, 53-H) ppm; ^{31}**P-NMR** (121 MHz, D_2O, 300 K): δ = -9.80 (br. s, γ-P), -11.32 (d, α-P), -22.56 (br. s, β-P) ppm; **ESI(-)-MS** (*m/z*): calc.: 1509.38 [$C_{61}H_{79}N_{10}O_{25}P_3S_2$], found: 508.1 $[M+H_2O]^{3-}$, 722.9 $[M-PO_4+Na]^{2-}$.

2 Spectra and chromatograms

2.1 5-(Prop-2-ynyl)carbamic acid-1-{2-[2-(2-amino-ethoxy)ethoxy]ethoxymethyl}-2-cyanoethyl ester-3'-*O*-(2-cyanoethyl)2'-desoxyuridine 5'-triphosphate 28

Analytical RP-HPLC chromatogram:

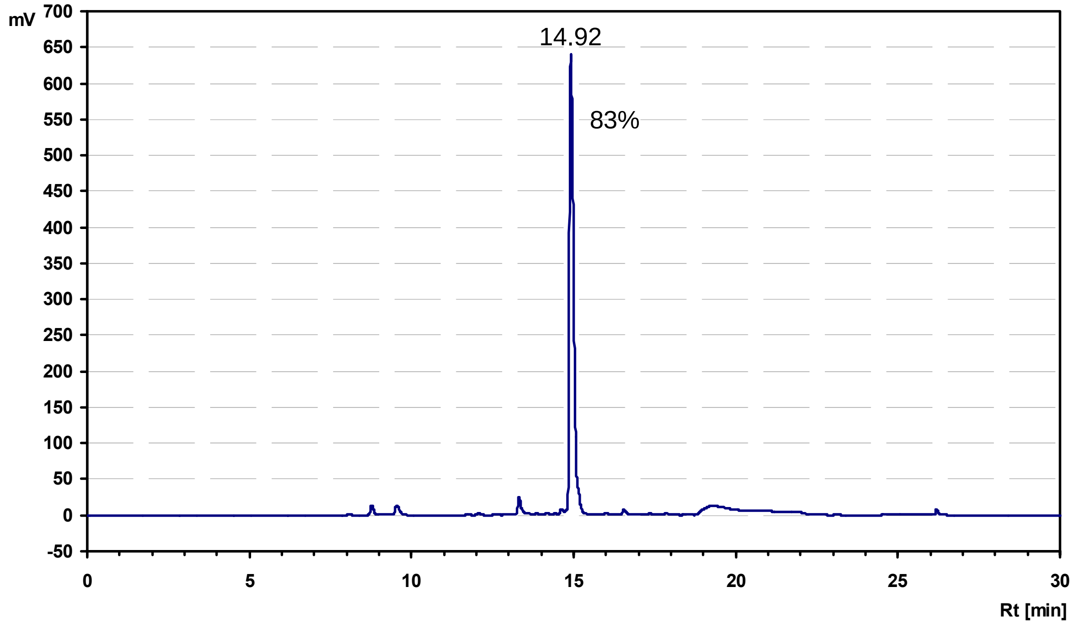

^1H-NMR (D_2O):

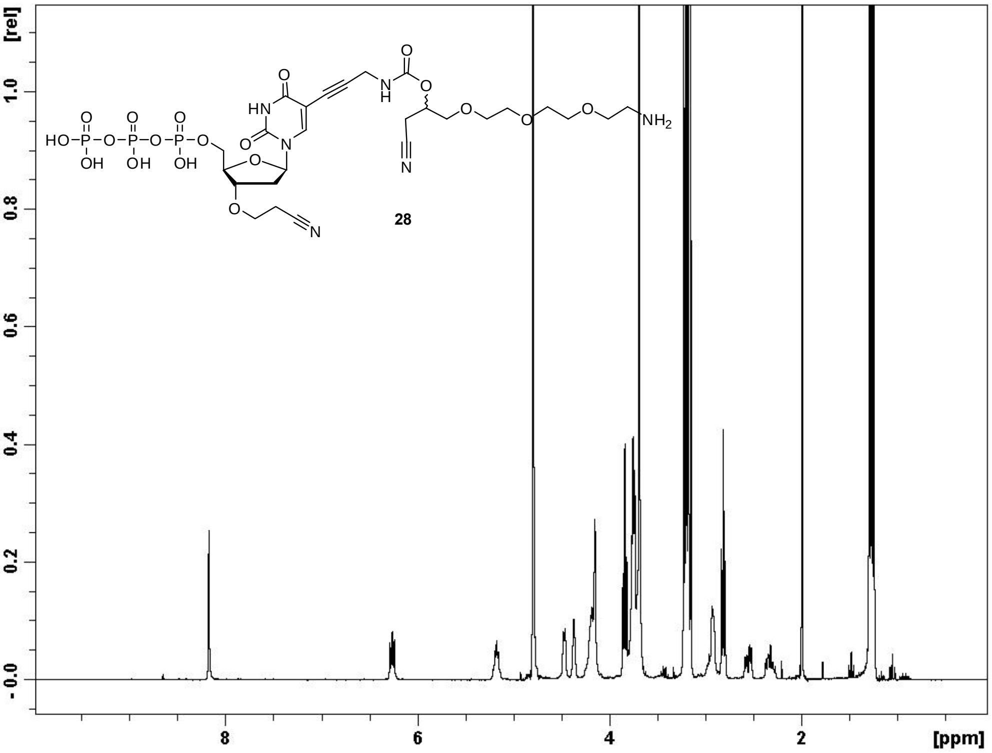

^{13}C-NMR (D_2O):

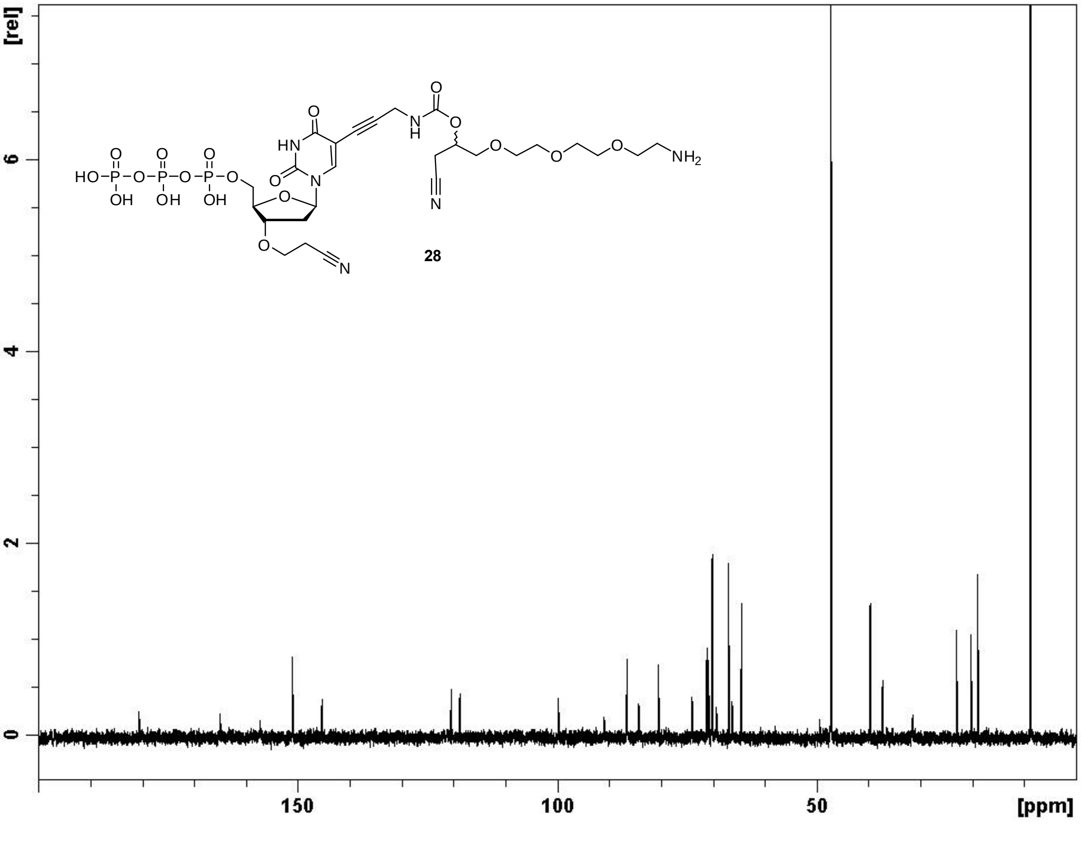

^{31}P-NMR (D_2O):

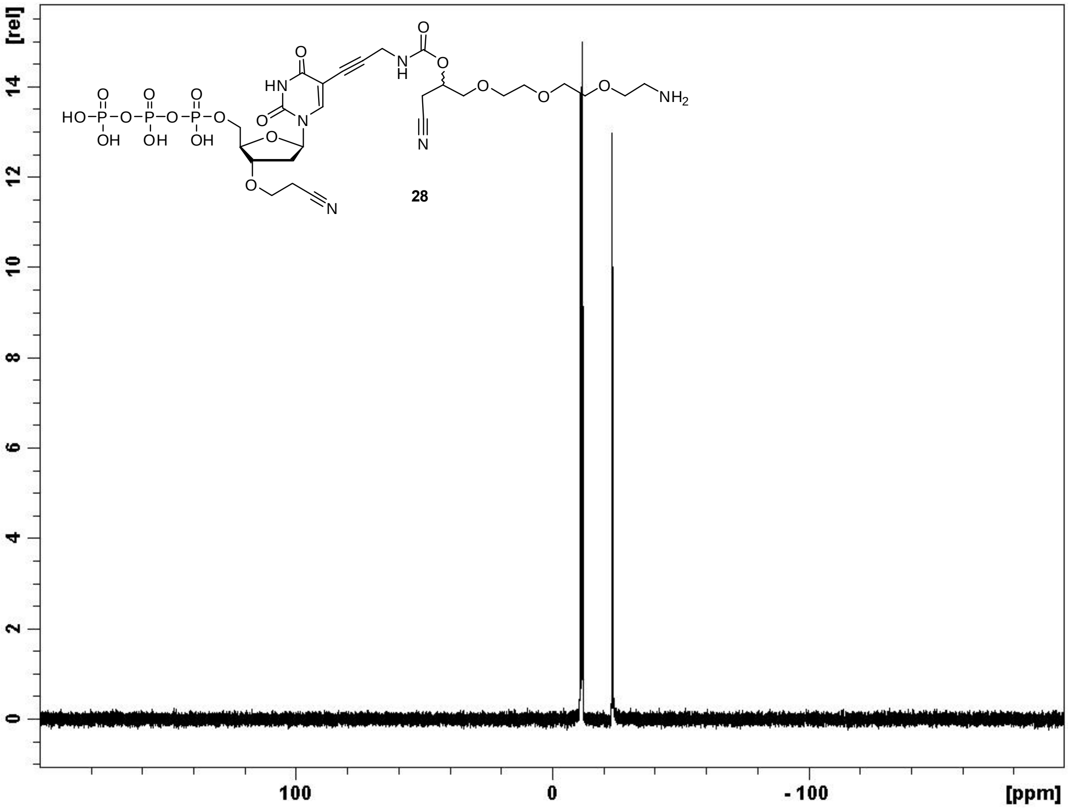

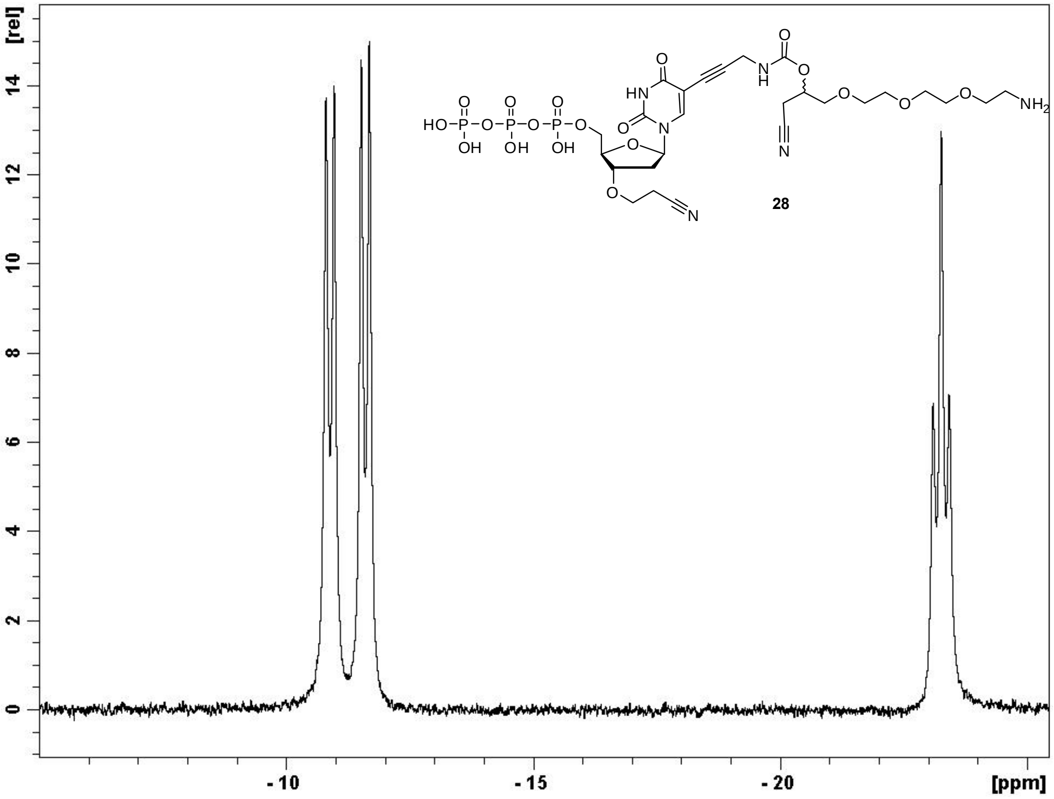

ESI-MS spectrum:

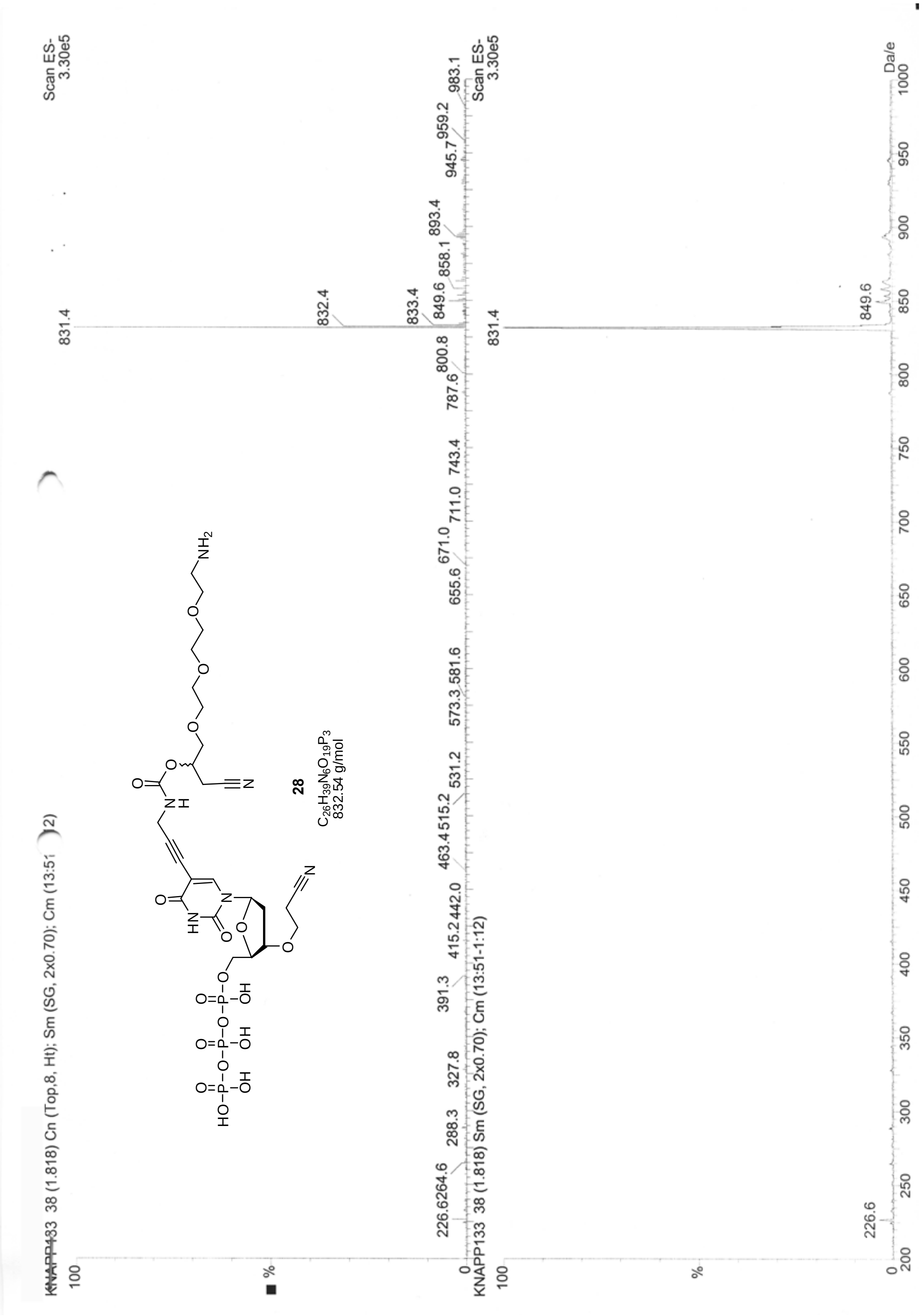
KNAPP133 38 (1.818) Cn (Top,8, Ht); Sm (SG, 2x0.70); Cm (13:51 2)
Scan ES-
3.30e5
100
%
0
226.6
264.6
288.3
327.8
391.3
415.2
442.0
463.4
515.2
531.2
573.3
581.6
655.6
671.0
711.0
743.4
787.6
800.8
831.4
832.4
833.4
849.6
858.1
893.4
945.7
959.2
983.1
28
$C_{26}H_{39}N_6O_{19}P_3$
832.54 g/mol
KNAPP133 38 (1.818) Sm (SG, 2x0.70); Cm (13:51-1:12)
Scan ES-
3.30e5
100
%
0
226.6
831.4
849.6
200
250
300
350
400
450
500
550
600
650
700
750
800
850
900
950
1000
Da/e

2.2 5-(Prop-2-ynyl)carbamic acid-1-{2-[2-(2-5- and 6-carboxyfluoresceinamidoethoxy)ethoxy]ethoxymethyl}-2-cyanoethyl ester-3′-*O*-(2-cyanoethyl)-2′-deoxyuridine 5′-triphosphate 2

Analytical RP-HPLC chromatogram:

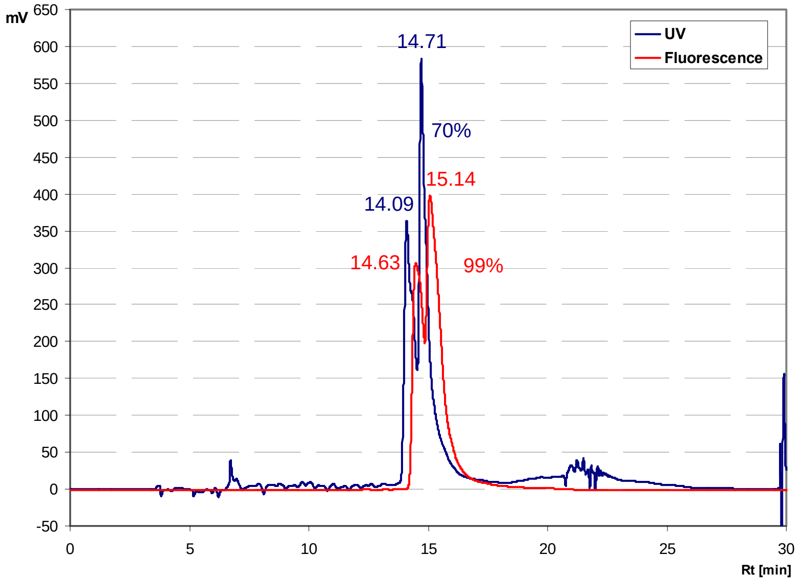

Fluorescence spectrum (acquisition at 25 °C in deionised water):

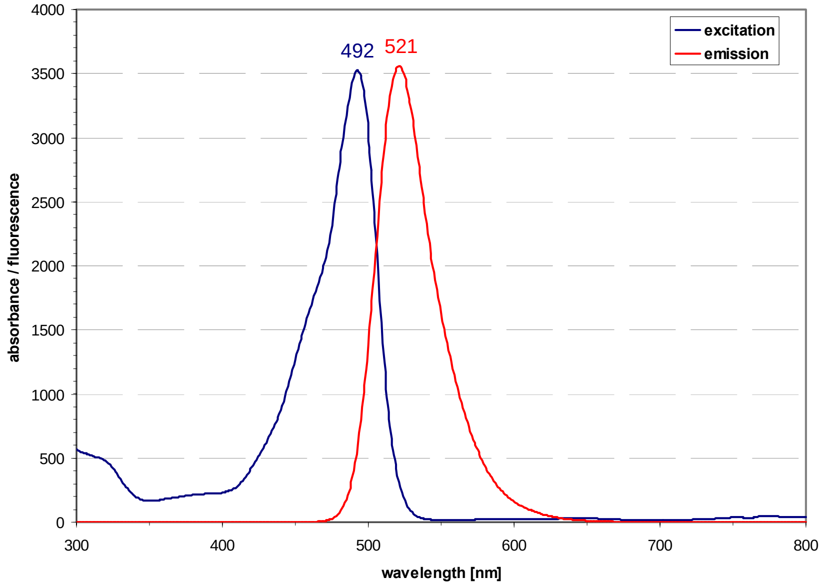

^{1}H-NMR (D_2O):

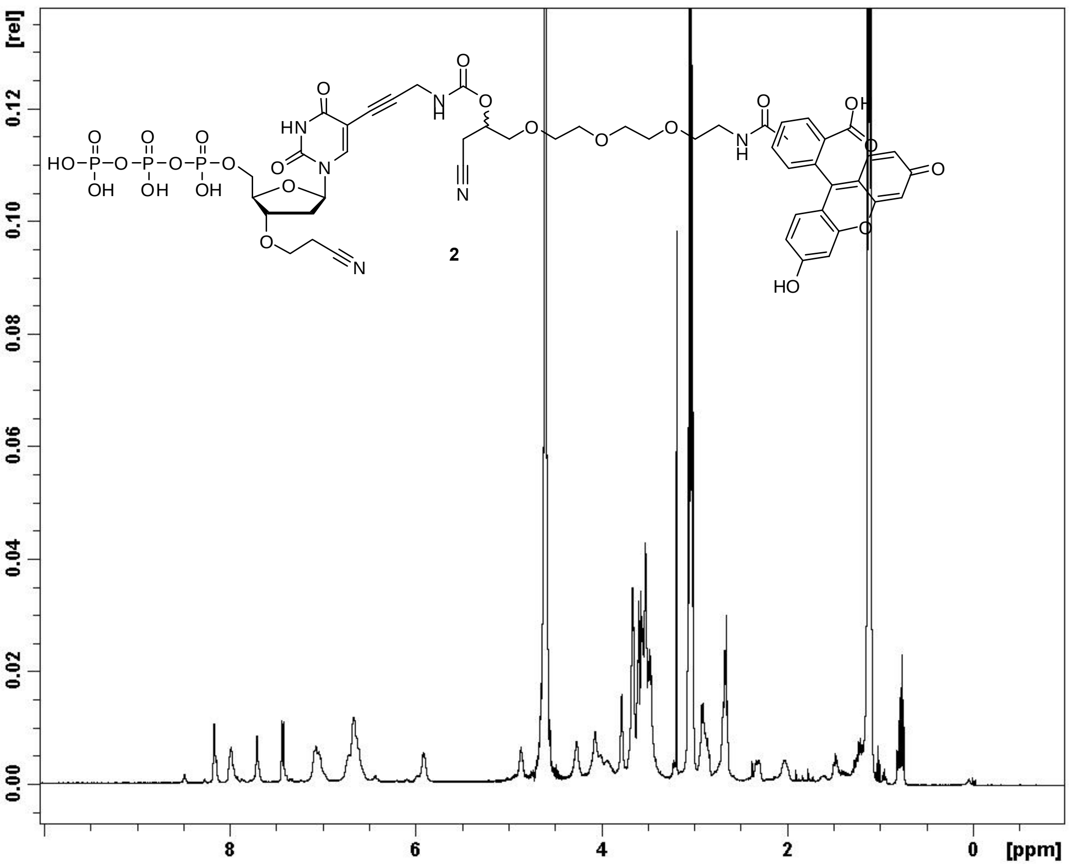

^{31}P-NMR (D_2O):

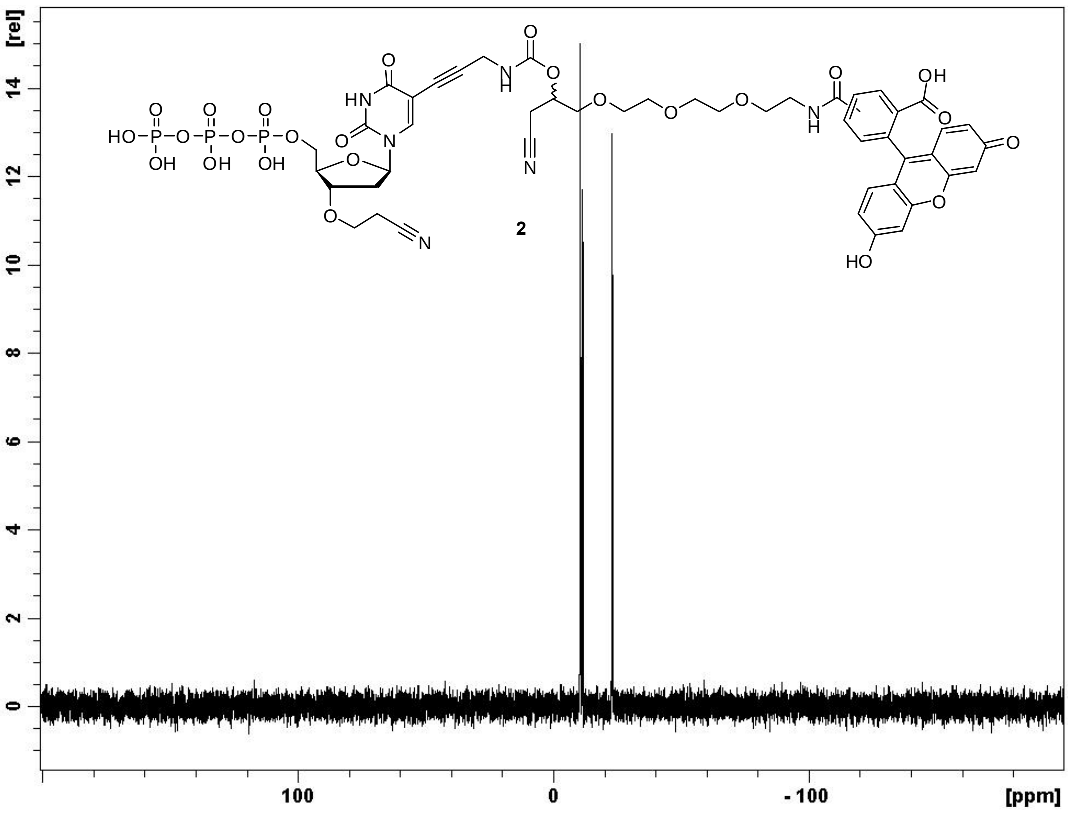

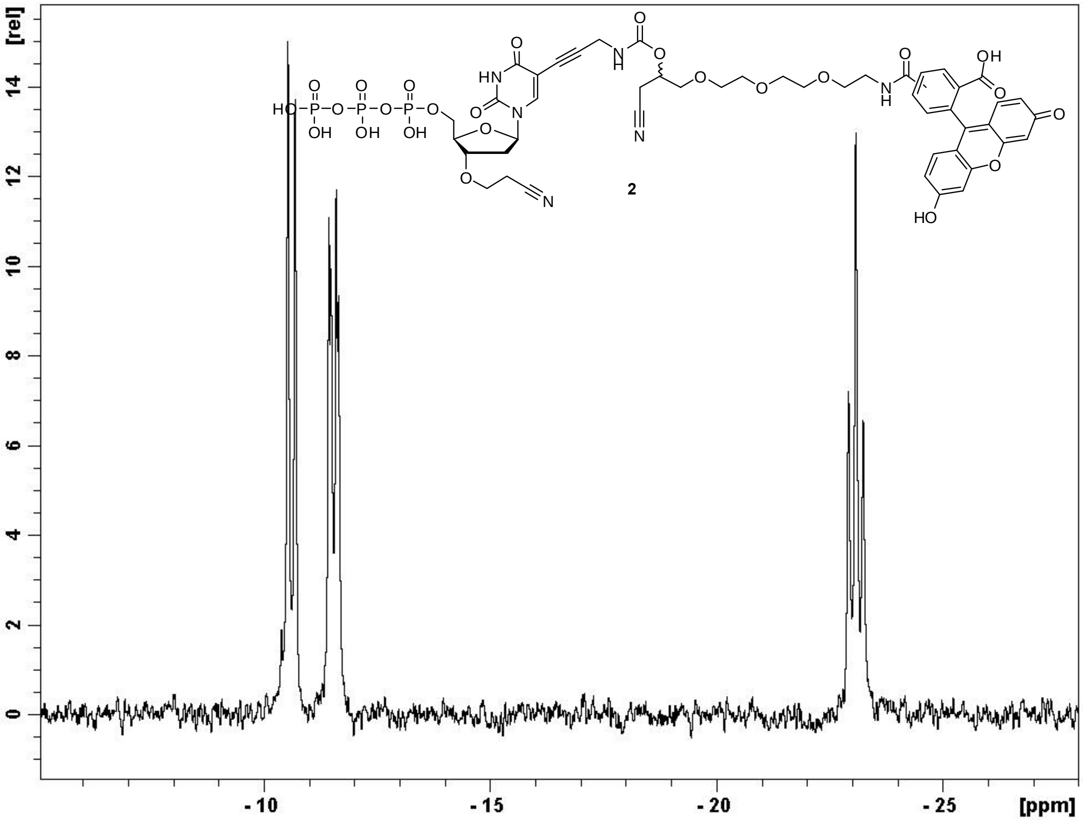
[rel]
14
12
10
8
6
4
2
0
- 10
- 15
- 20
- 25
[ppm]
2

ESI-MS spectrum:

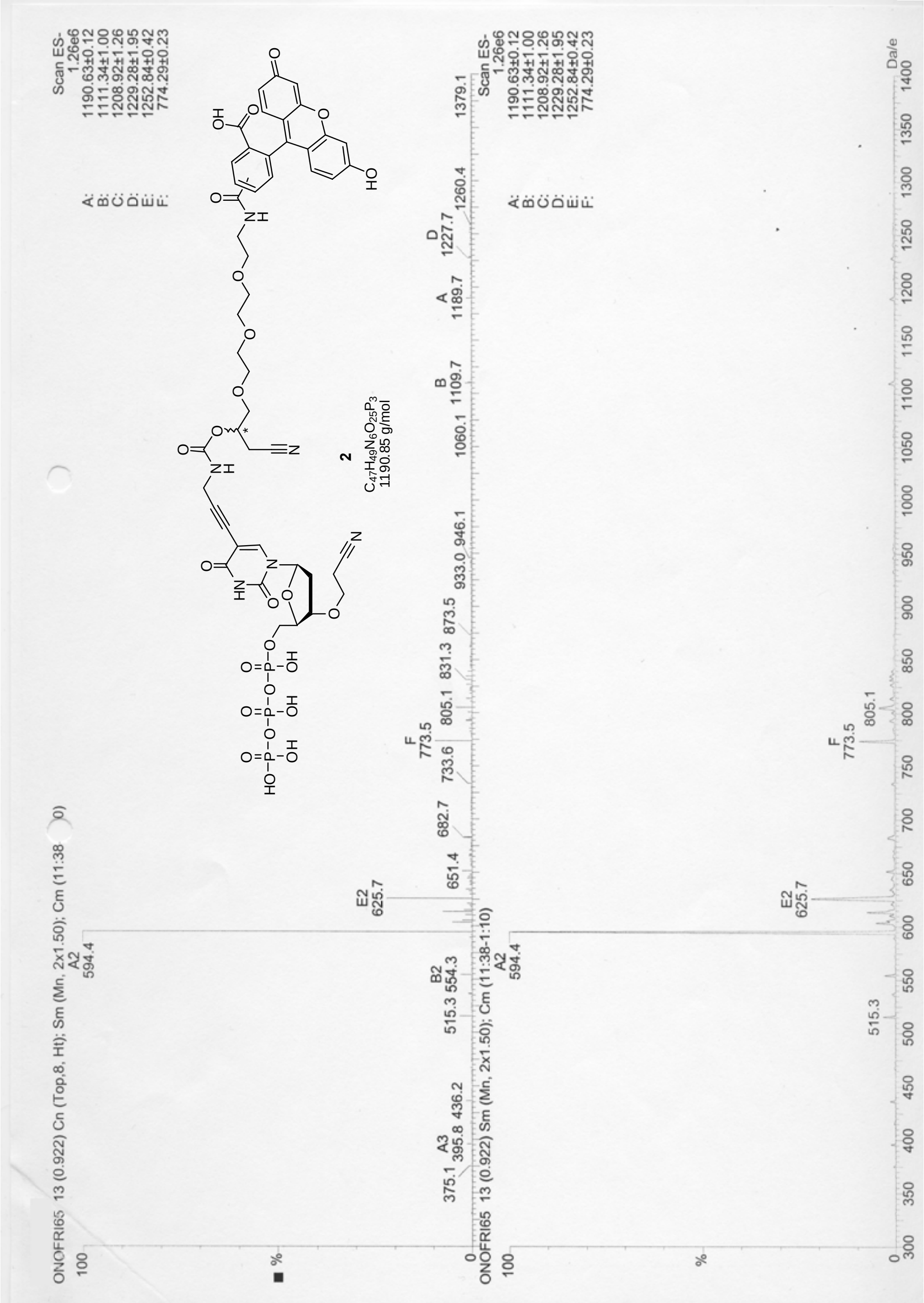

2.3 7-Deaza-7-(prop-2-ynyl)carbamic acid-1-{2-[2-(2-*tert*-butoxycarbonylaminoethoxy)ethoxy]ethoxy-methyl}-2-cyanoethyl ester-3'-*O*-(2-cyanoethyl)-2'-desoxy-adenosine 5'-triphosphate 30

Analytical RP-HPLC chromatogram:

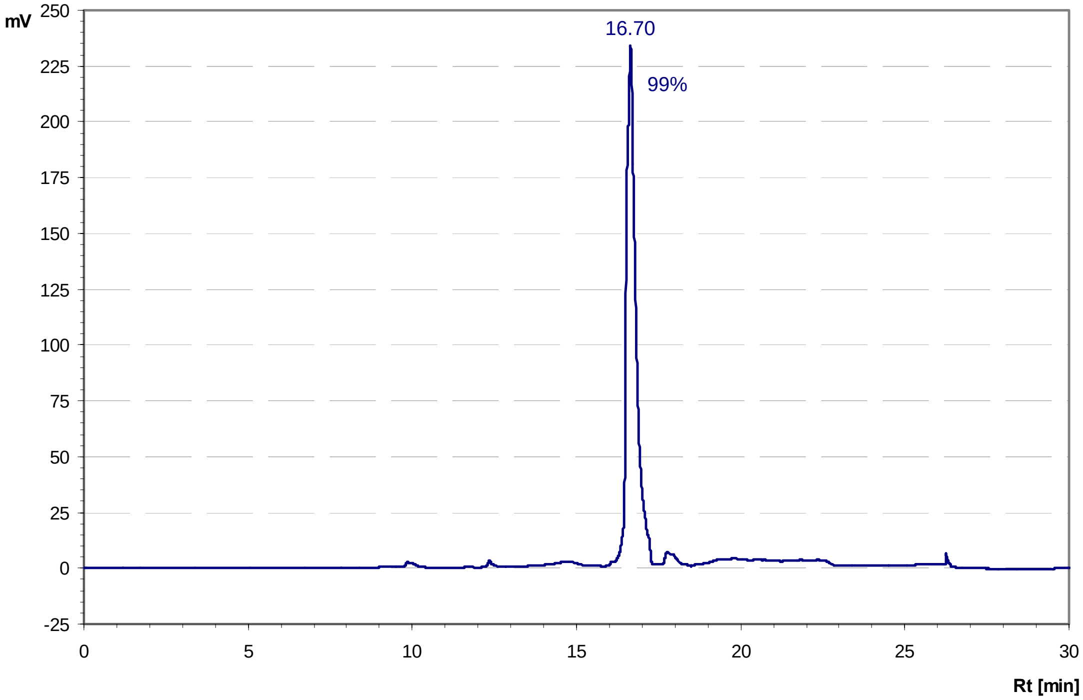

^1H-NMR (D_2O):

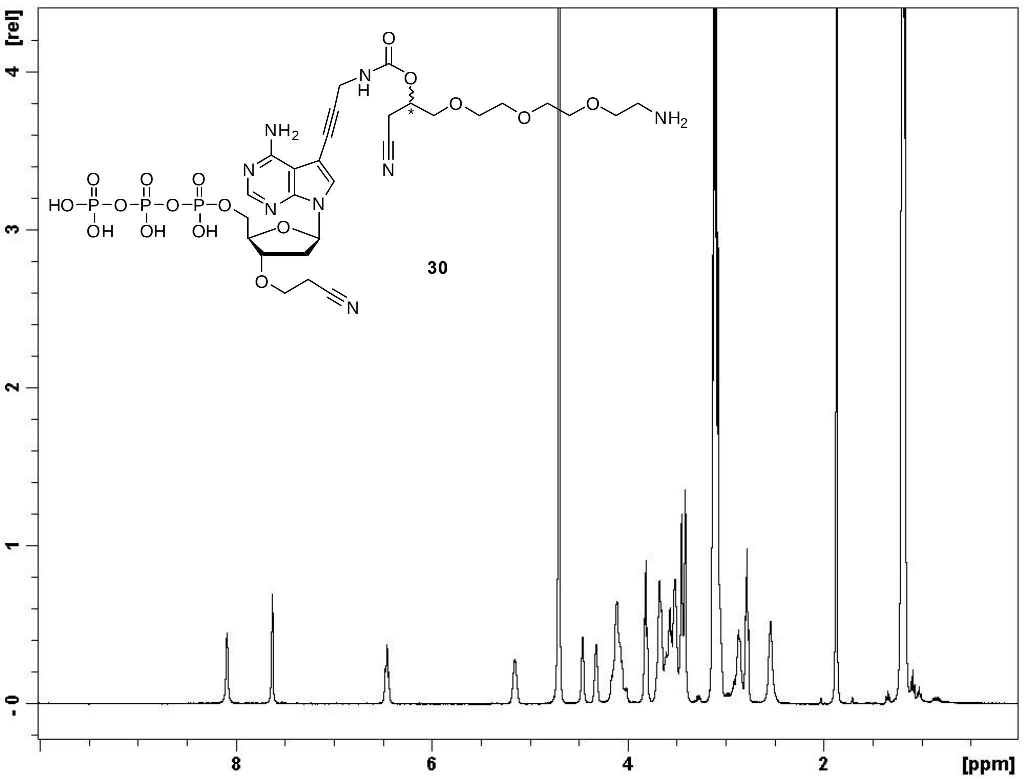

^{13}C-NMR (D_2O):

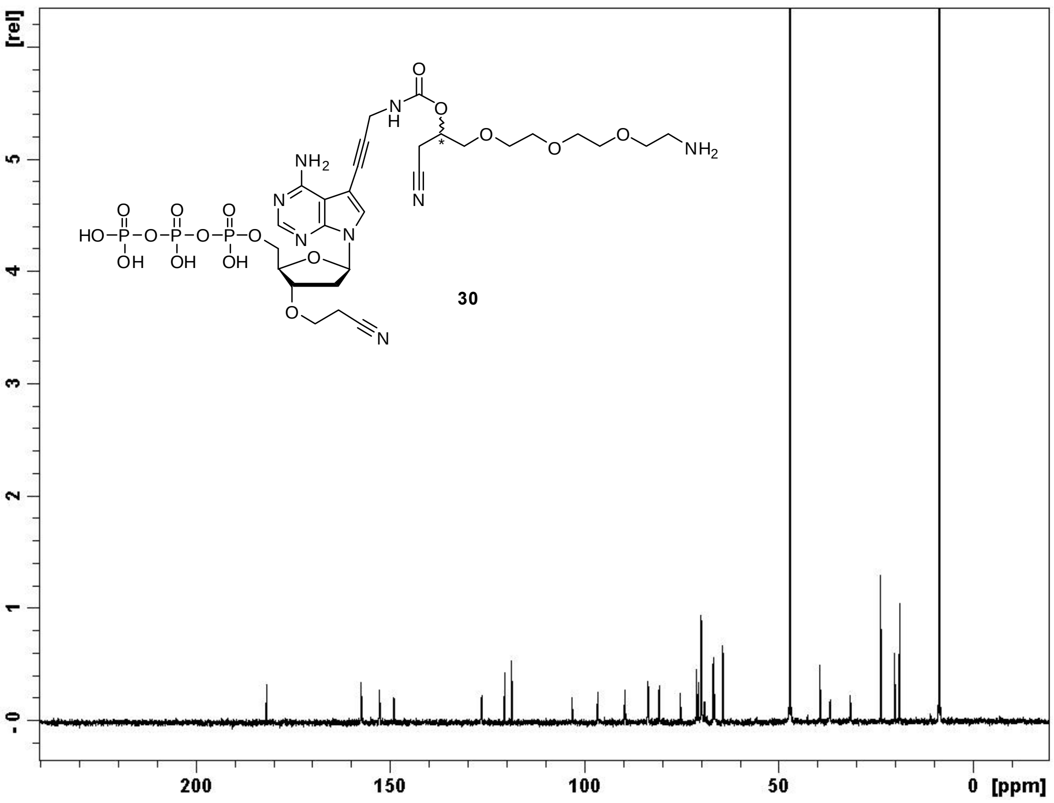

^{31}P-NMR (D_2O):

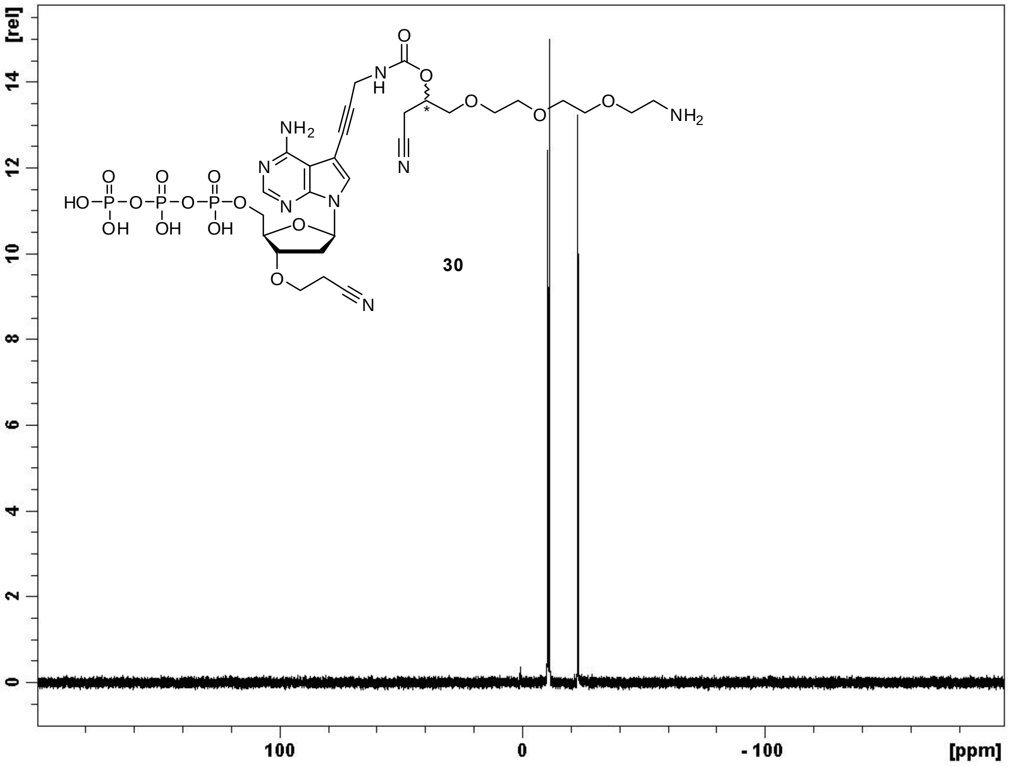

[rel]
14
12
10
8
6
4
2
0
100
0
- 100
[ppm]
NH$_2$
HO
OH
N
H
O
NH$_2$
30

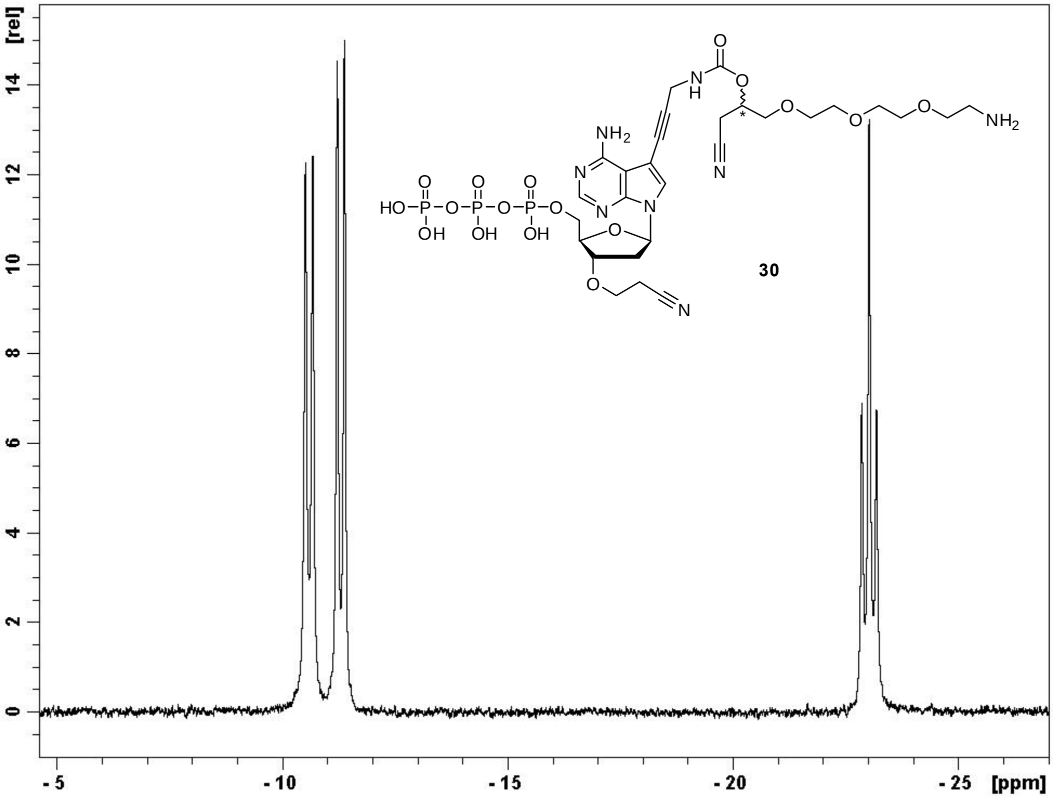

[rel]
14
12
10
8
6
4
2
0
- 5
- 10
- 15
- 20
- 25
[ppm]
NH$_2$
HO
OH
N
H
O
NH$_2$
30

ESI-MS spectrum:

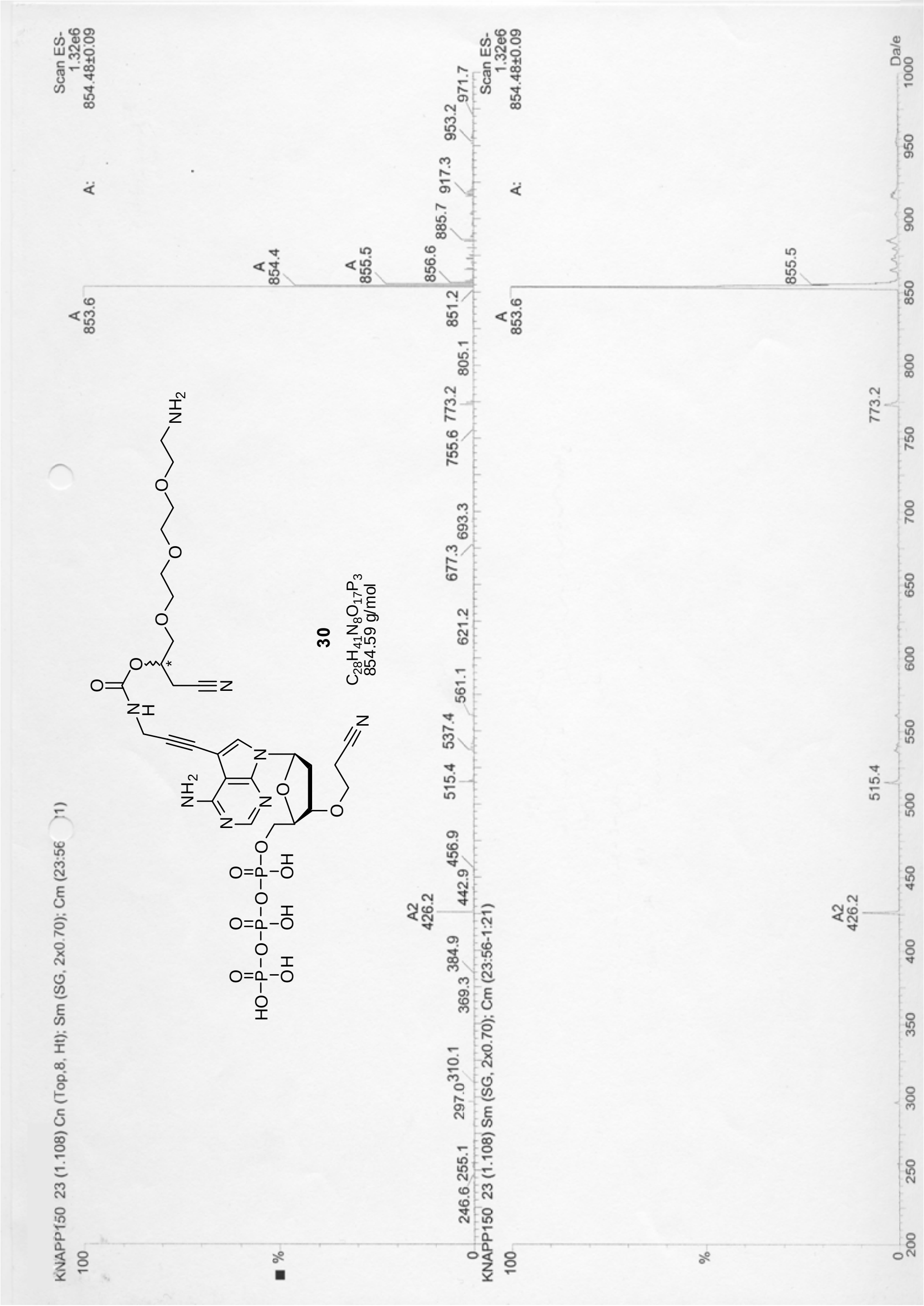

2.4 7-Deaza-7-(prop-2-ynyl)carbamic acid-1-{2-[2-(2-5- and 6-carboxy-X-rhodamineamidoethoxy)ethoxy]-ethoxymethyl}-2-cyanoethyl ester-3'-*O*-(2-cyanoethyl)-2'-desoxyadenosine 5'-triphosphate 3

Analytical RP-HPLC chromatogram:

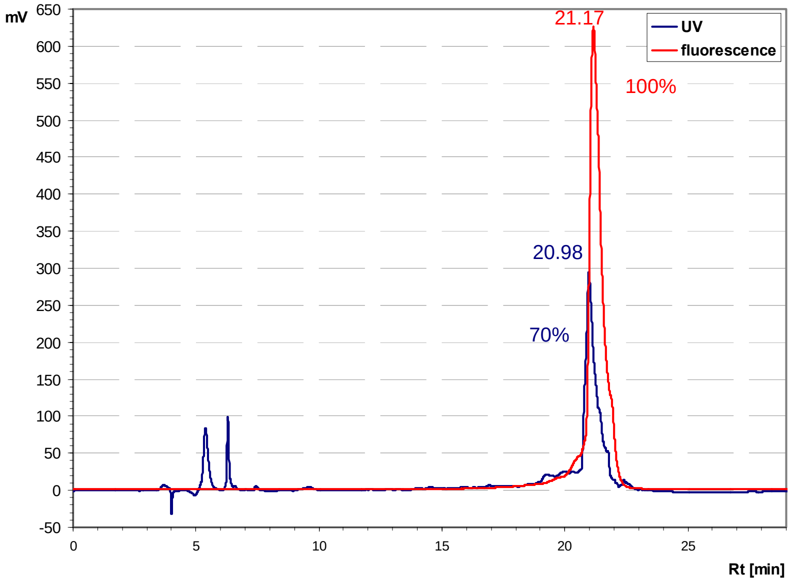

Fluorescence spectrum (acquisition at 25 °C in deionised water):

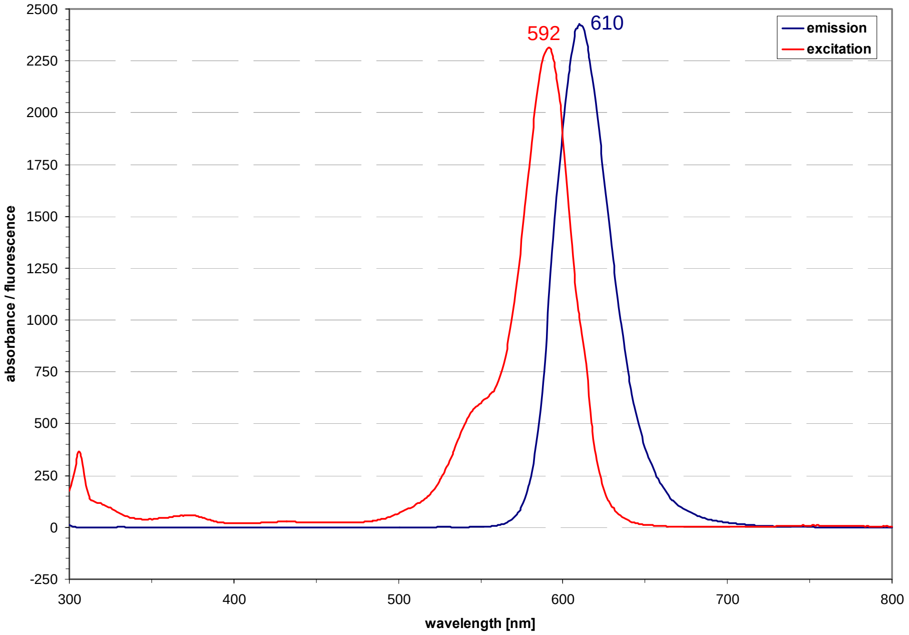

^1H-NMR (D_2O):

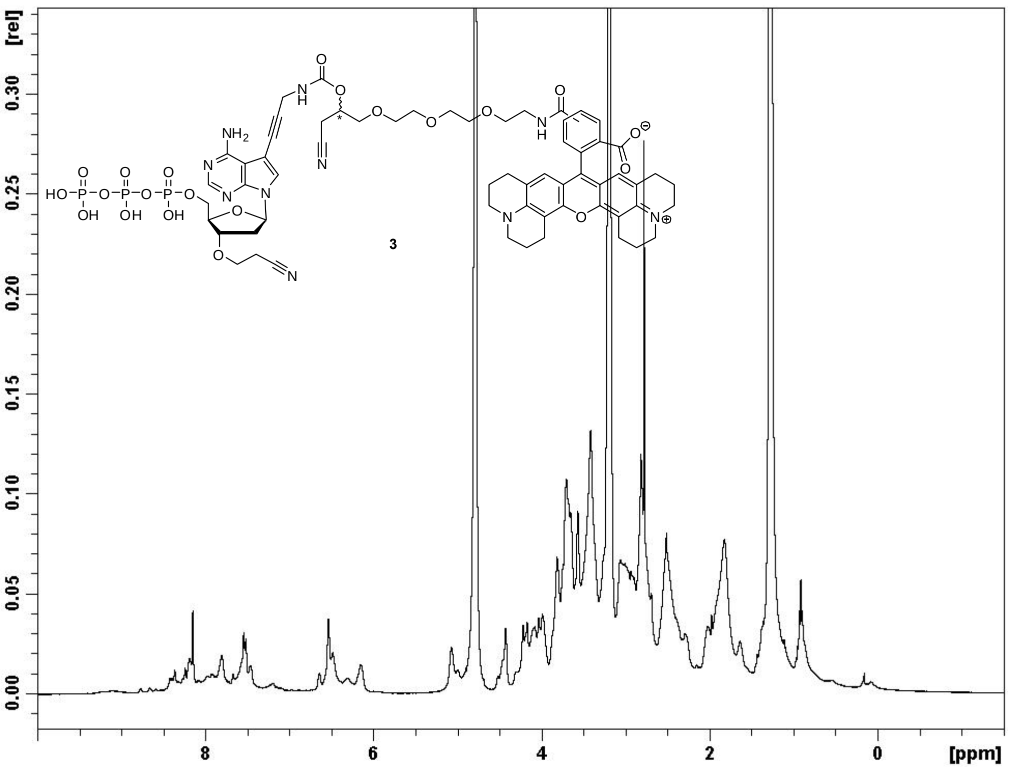

^{31}P-NMR (D_2O):

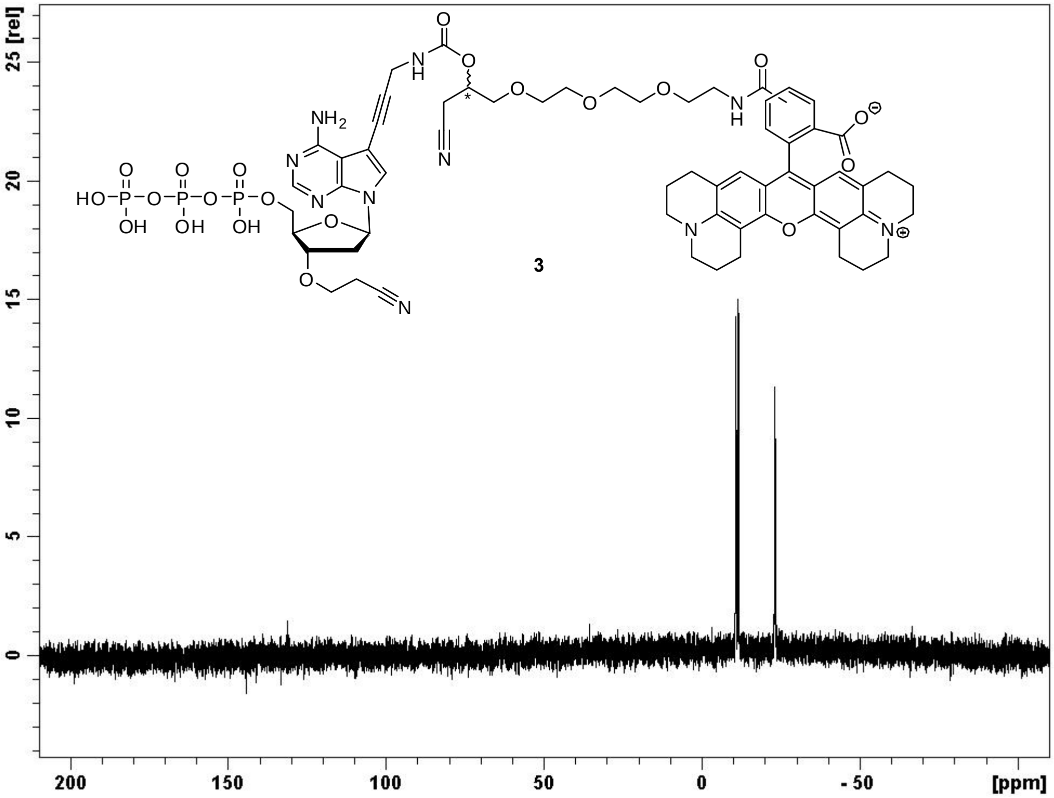

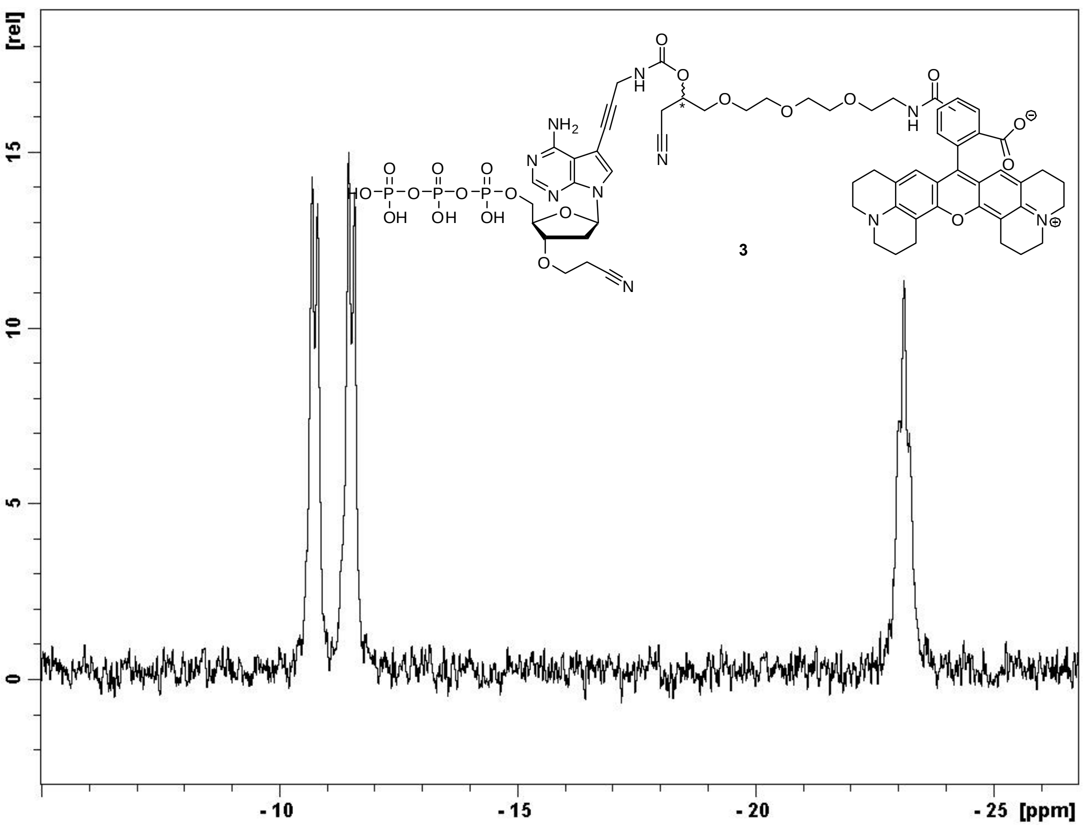
[rel]
15
10
5
0
- 10
- 15
- 20
- 25
[ppm]
3

ESI-MS spectrum:

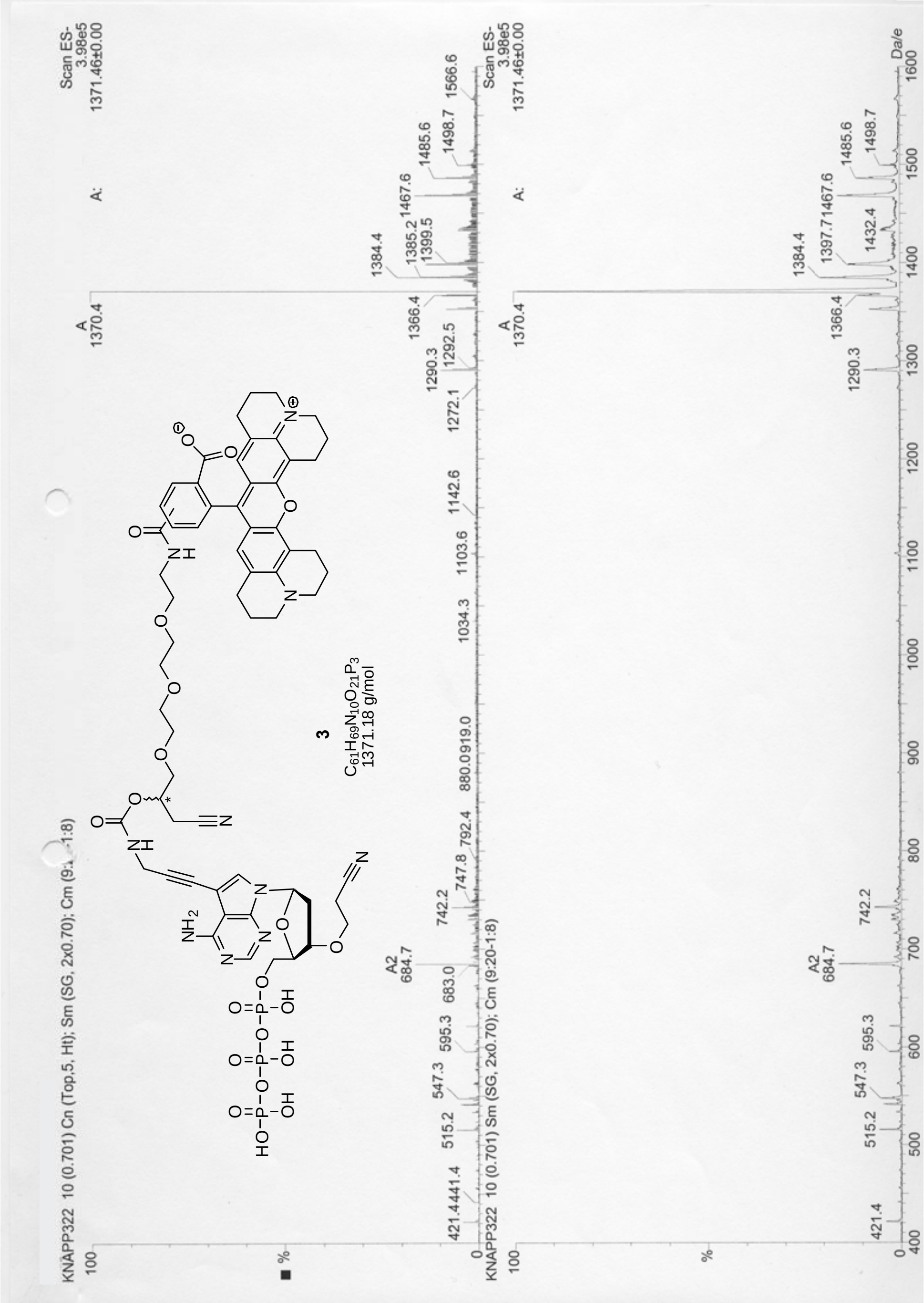

2.5 5-(Prop-2-ynyl)carbamic acid-1-{2-[2-(2-*tert*-butoxycarbonylaminoethoxy)ethoxy]-ethoxymethyl}-2-cyanoethyl ester-3'-*O*-(2-cyanoethyl)-2'-deoxycytidine 5'-triphosphate 29

Analytical RP-HPLC chromatogram:

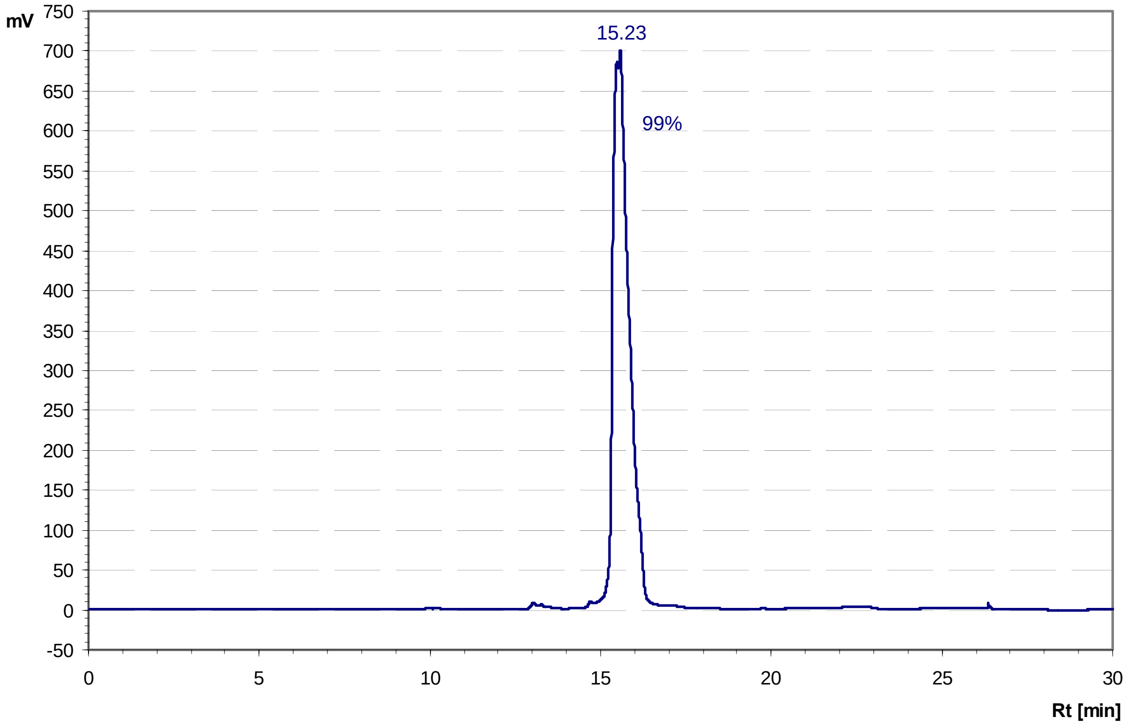

^{1}H-NMR (D_2O):

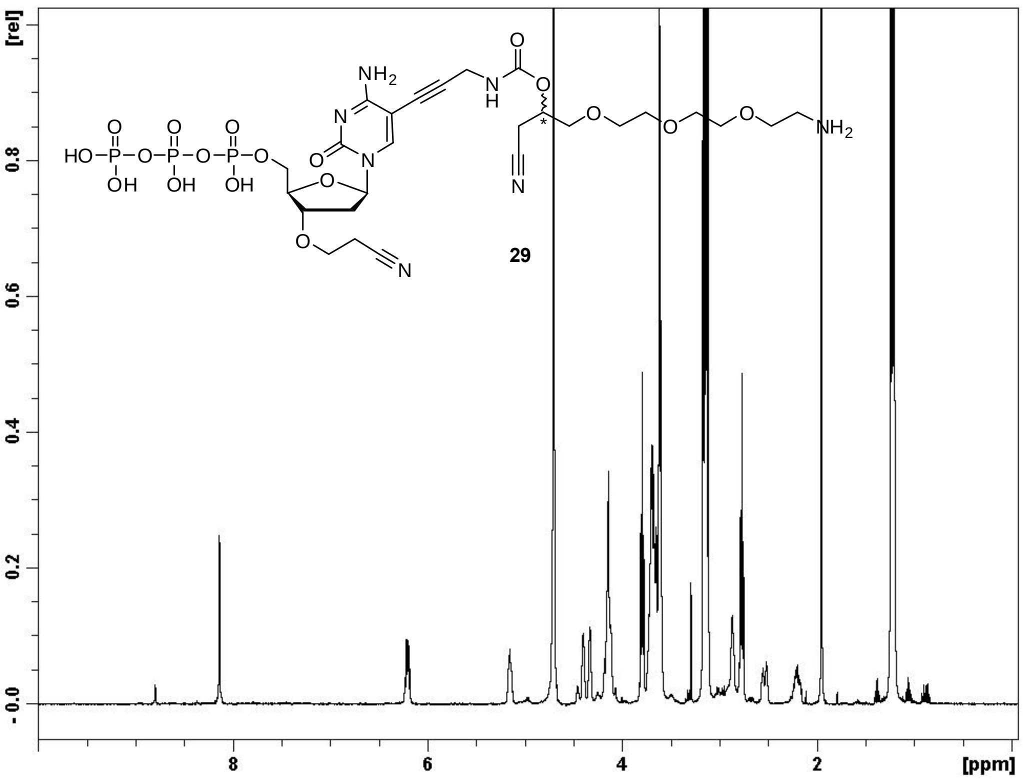

^{13}C-NMR (D_2O):

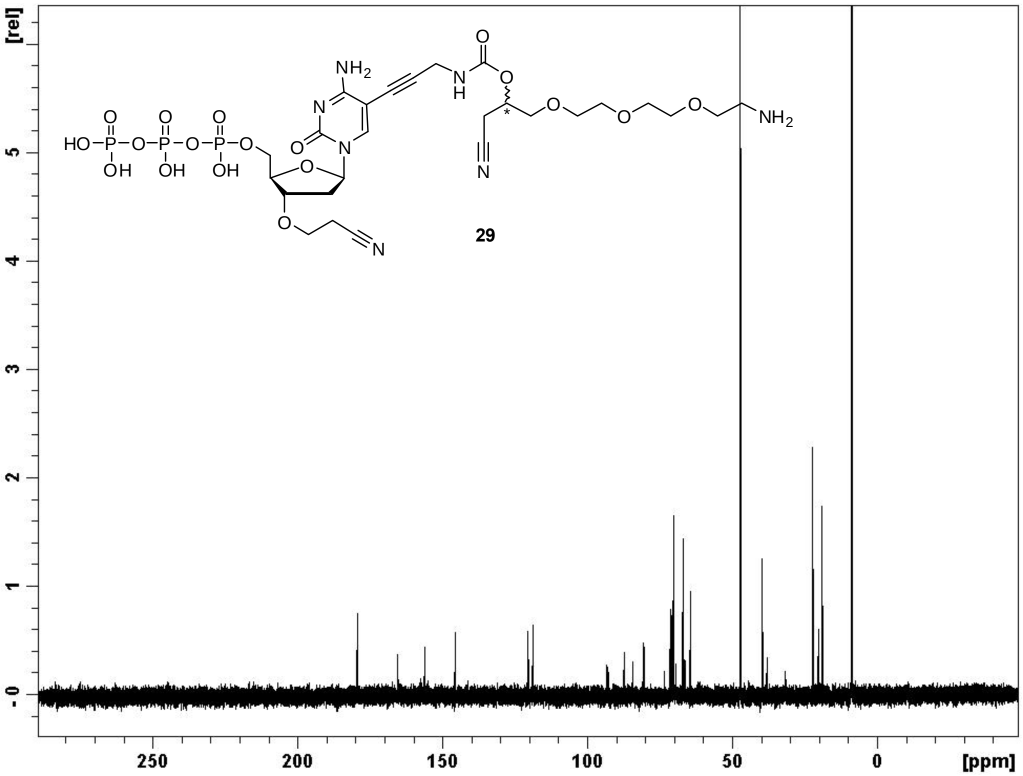

^{31}P-NMR (D_2O):

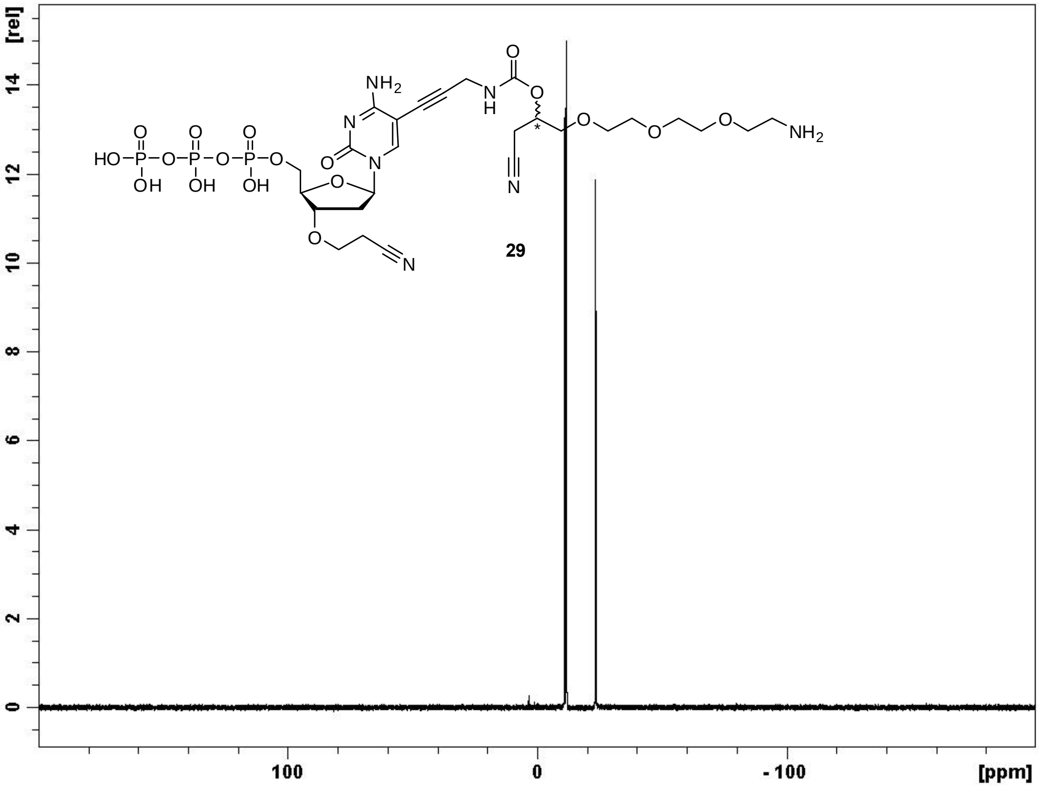

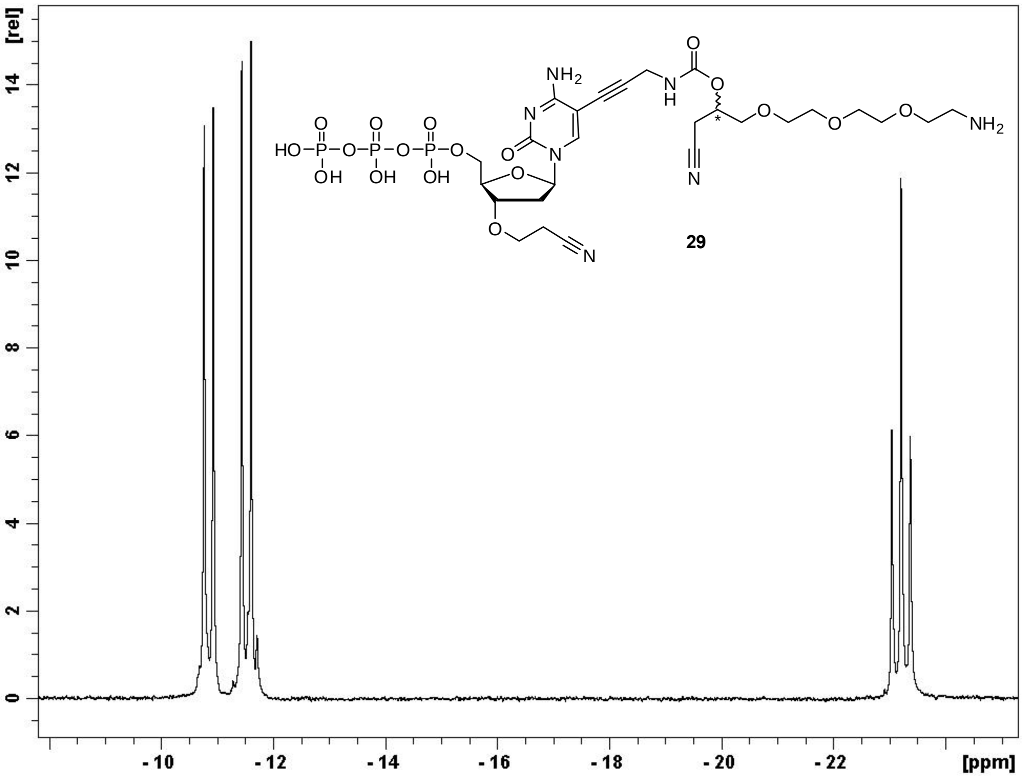

ESI-MS spectrum:

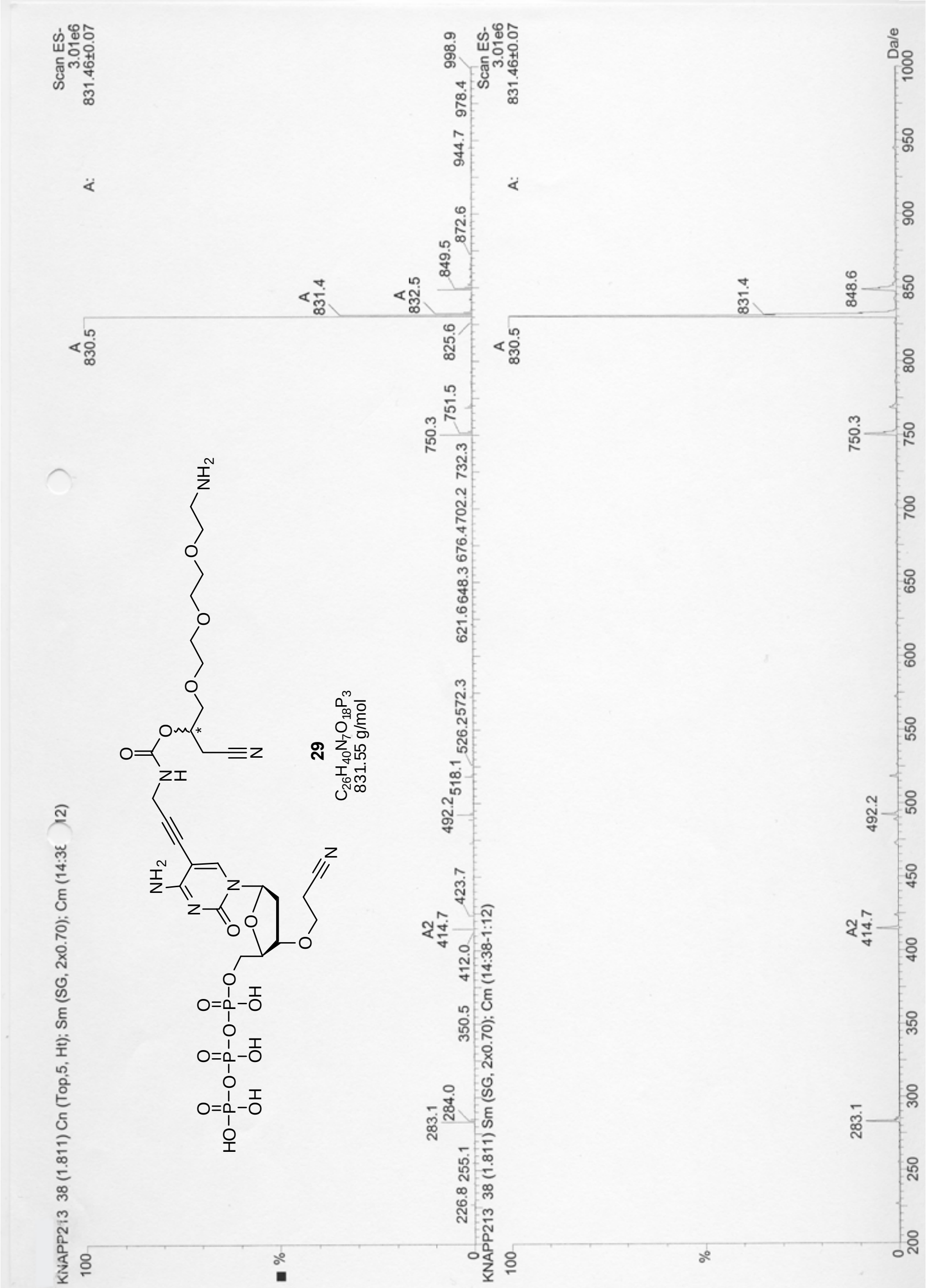

2.6 5-(Prop-2-ynyl)carbamic acid-1-{2-[2-(2-Cy 3.0-amidoethoxy)ethoxy]ethoxymethyl}-2-cyanoethyl ester-3'-*O*-(2-cyanoethyl)2'-deoxycytidine 5'-triphosphate 4

Analytical RP-HPLC chromatogram:

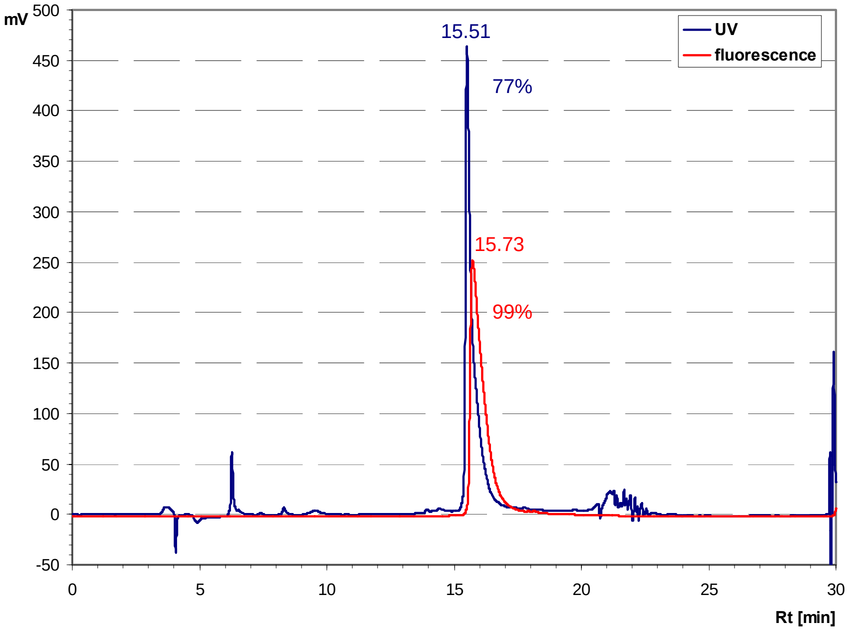

Fluorescence spectrum (acquisition at 25 °C in deionised water):

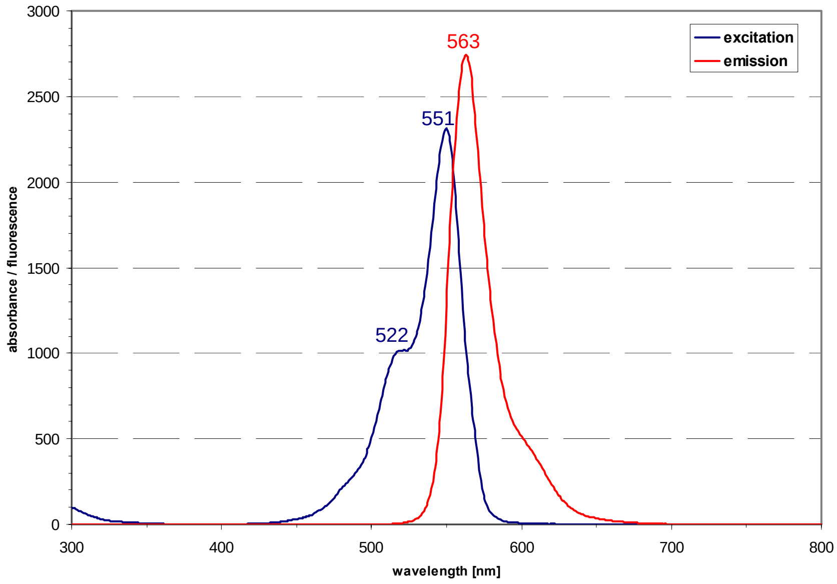

^{1}H-NMR (D_2O):

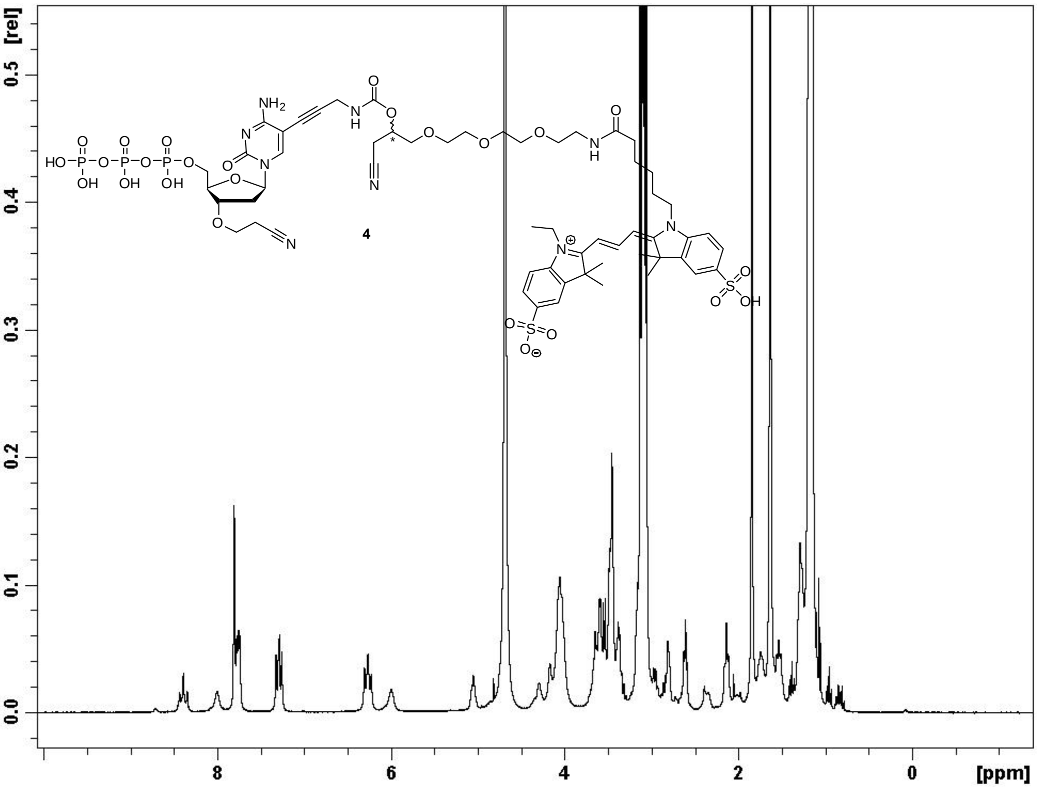

^{31}P-NMR (D_2O):

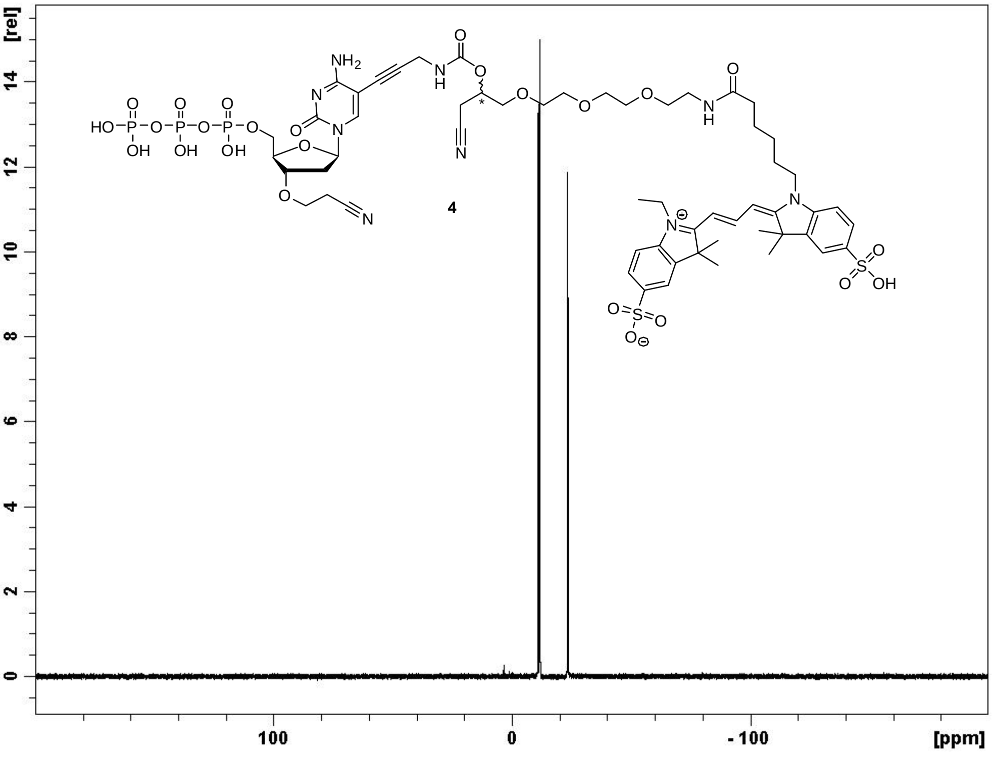

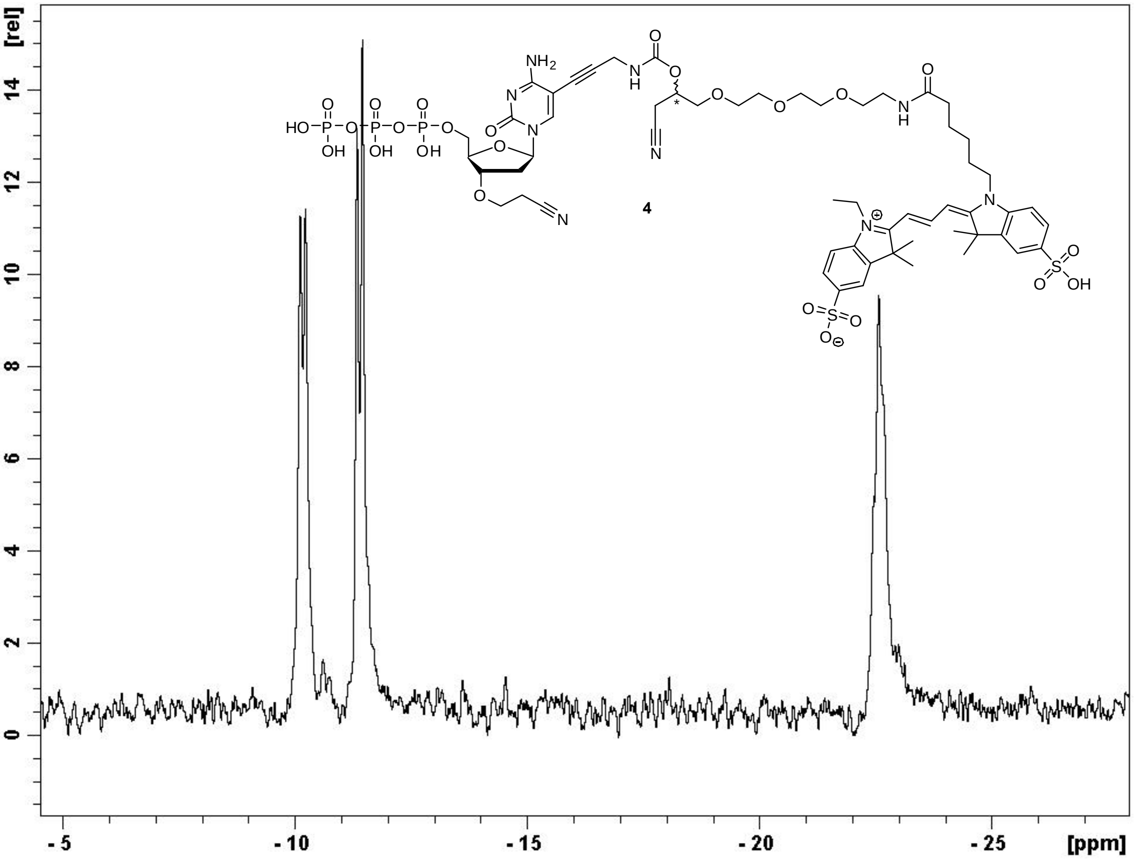
[rel]
14
12
10
8
6
4
2
0
- 5
- 10
- 15
- 20
- 25
[ppm]
4

ESI-MS spectrum:

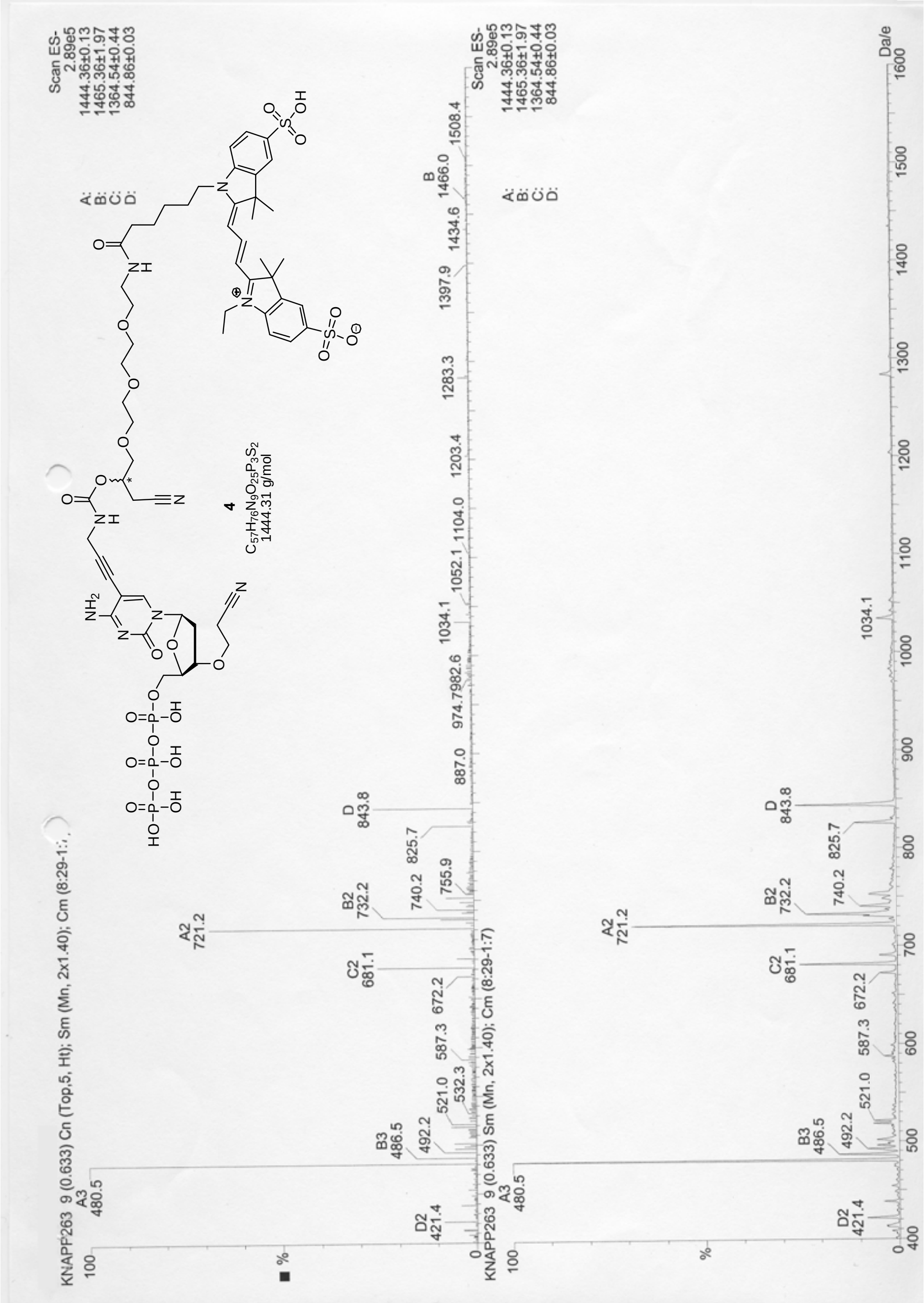

2.7 7-Deaza-7-(prop-2-ynyl)carbamic acid-1-{2-[2-(2-*tert*-butoxycarbonylaminoethoxy)ethoxy]ethoxymethyl}-2-cyanoethyl ester-3'-*O*-(2-cyanoethyl)-2'-desoxy-guanosine 5'-triphosphate 31

Analytical RP-HPLC chromatogram:

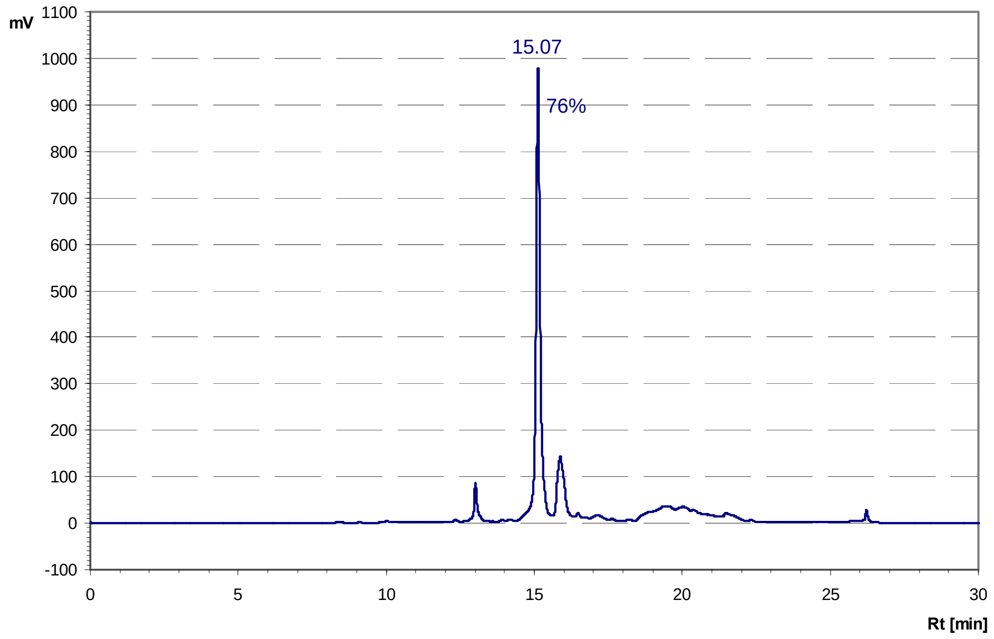

^{1}H-NMR (D_2O):

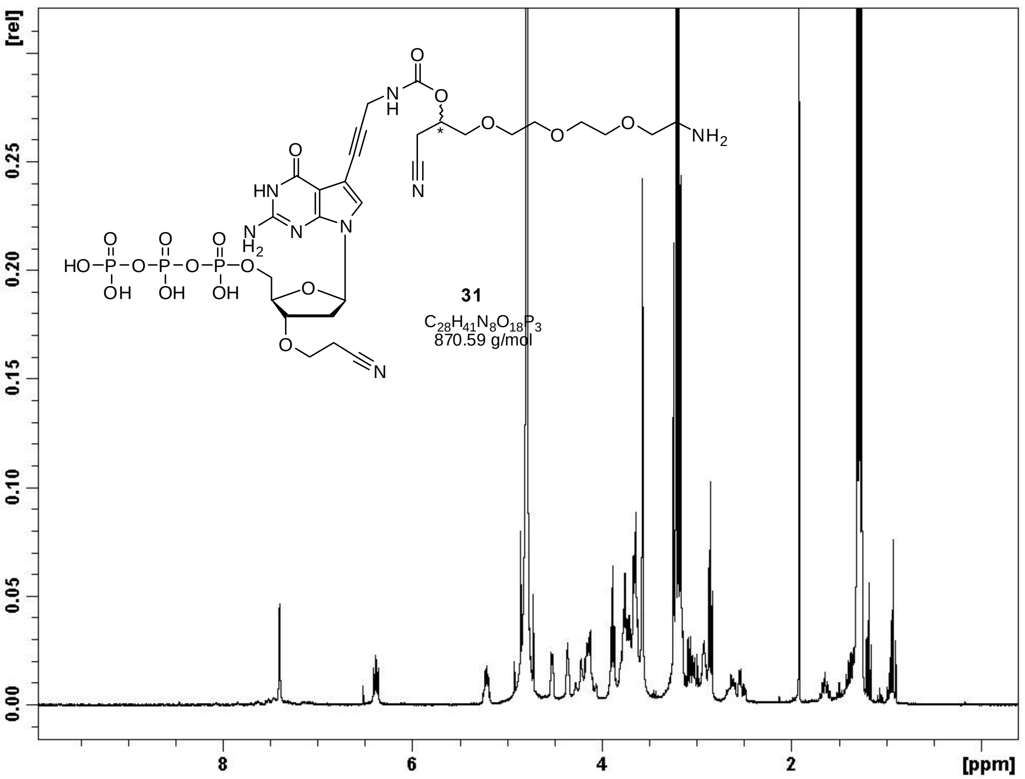

^{13}C-NMR (D_2O):

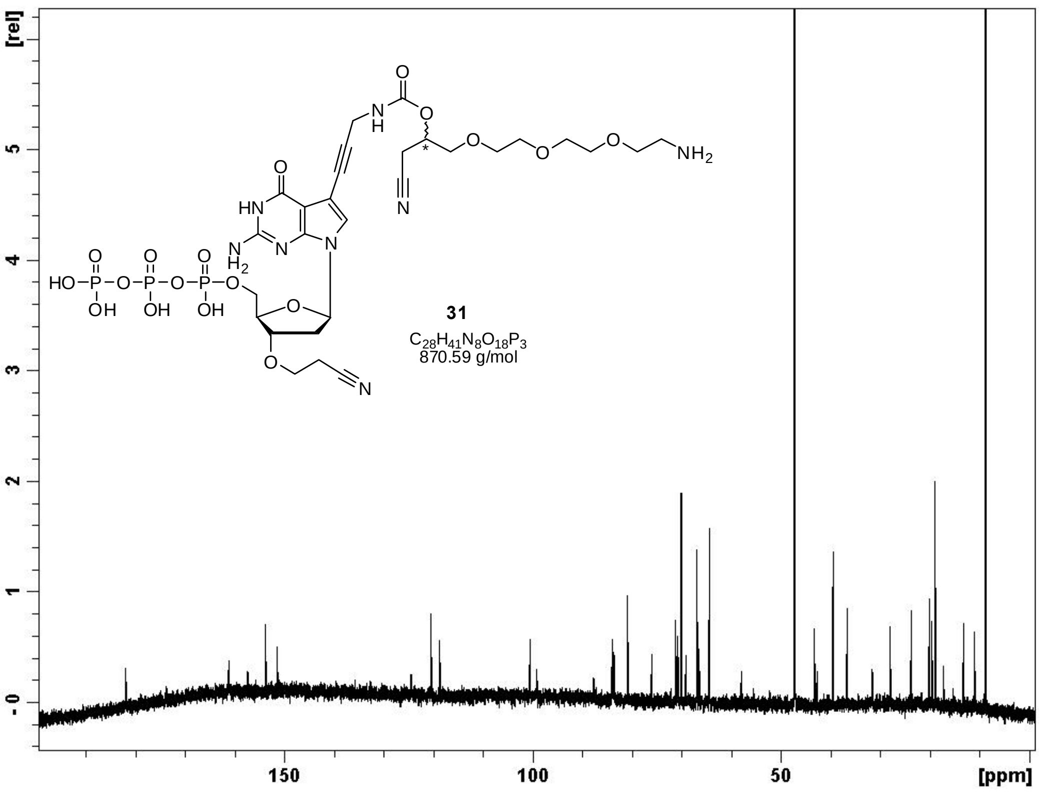

^{31}P-NMR (D_2O):

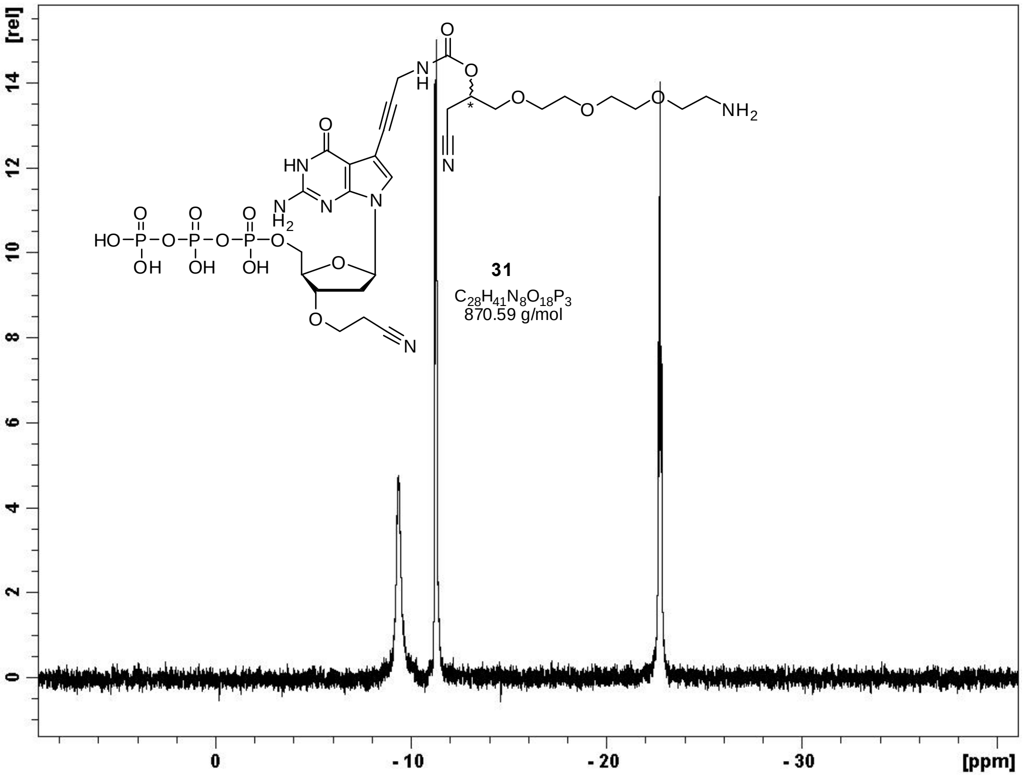

ESI-MS spectrum:

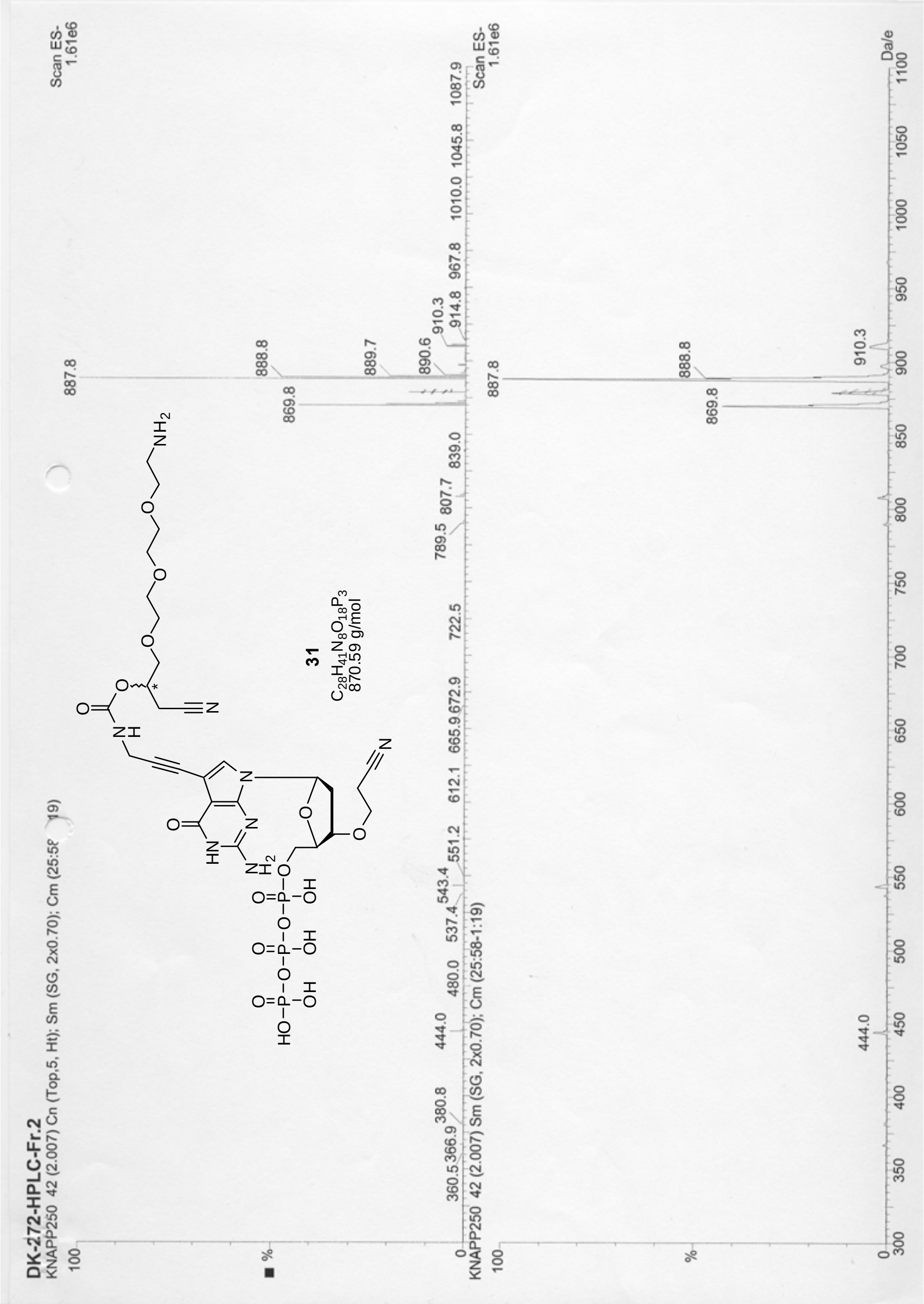

2.8 7-Deaza-7-(prop-2-ynyl)carbamic acid-1-{2-[2-(2-Cy 5.0-amidoethoxy)ethoxy]ethoxymethyl}-2-cyanoethyl ester-3'-*O*-(2-cyanoethyl)-2'-desoxyguanosine 5'-triphosphate 5

Analytical RP-HPLC chromatogram:

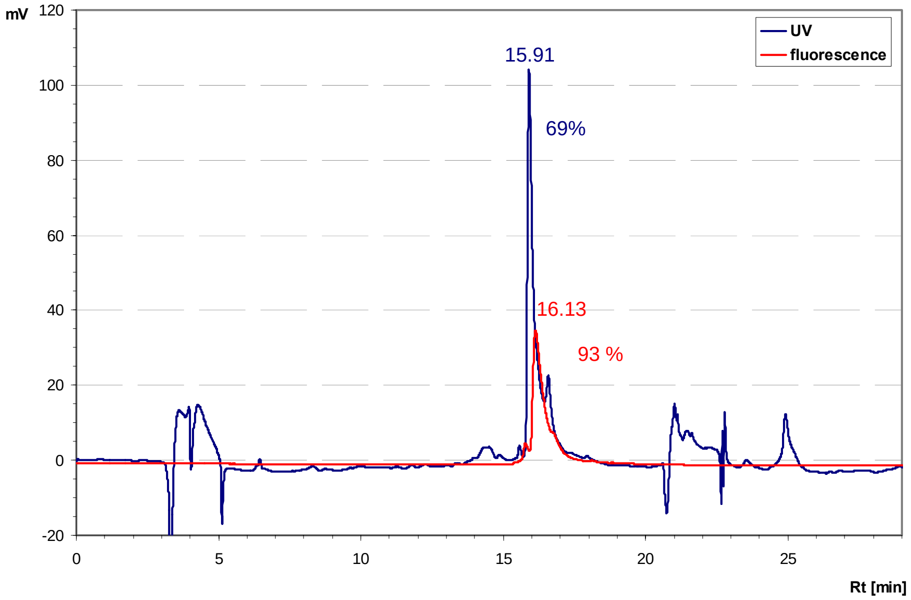

Fluorescence spectrum (acquisition at 25 °C in deionised water):

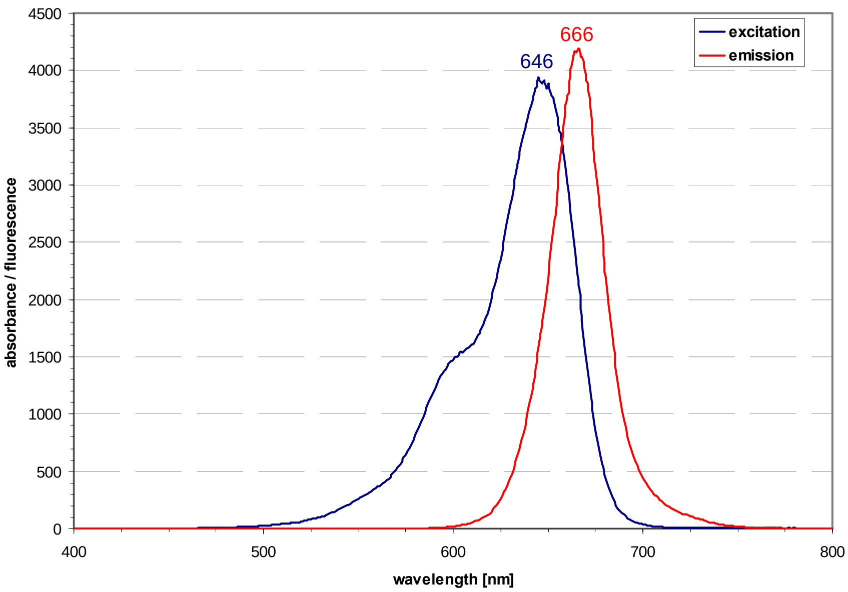

^{1}H-NMR (D_2O):

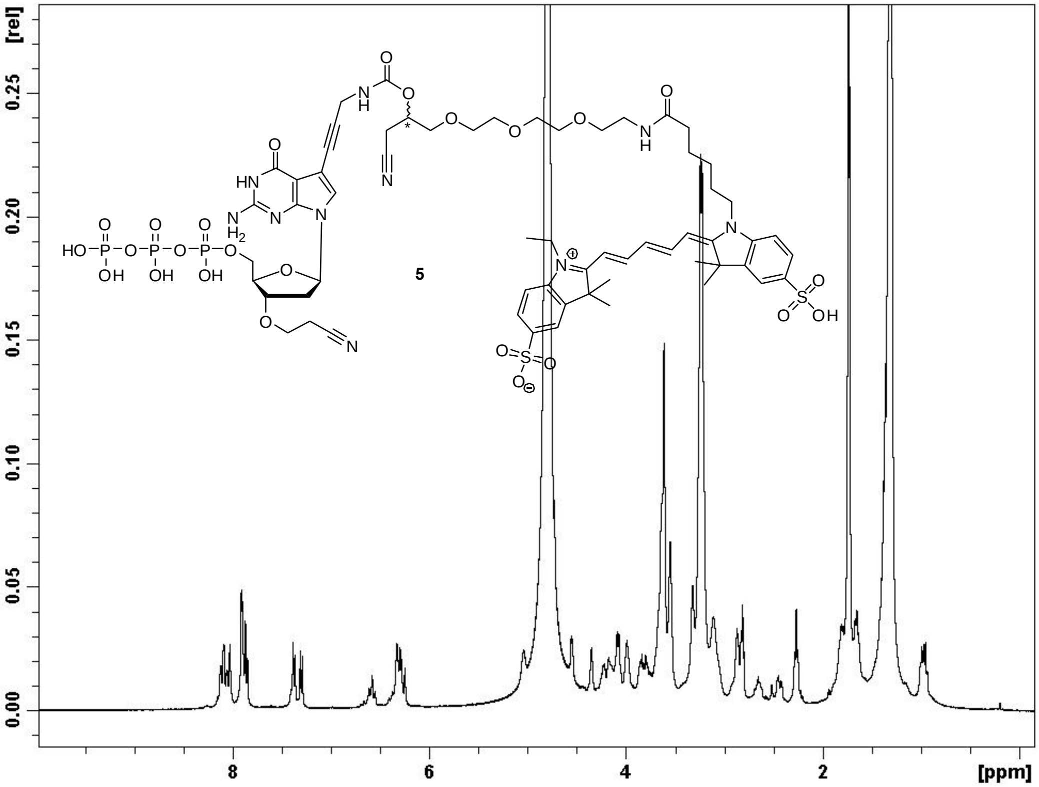

^{31}P-NMR (D_2O):

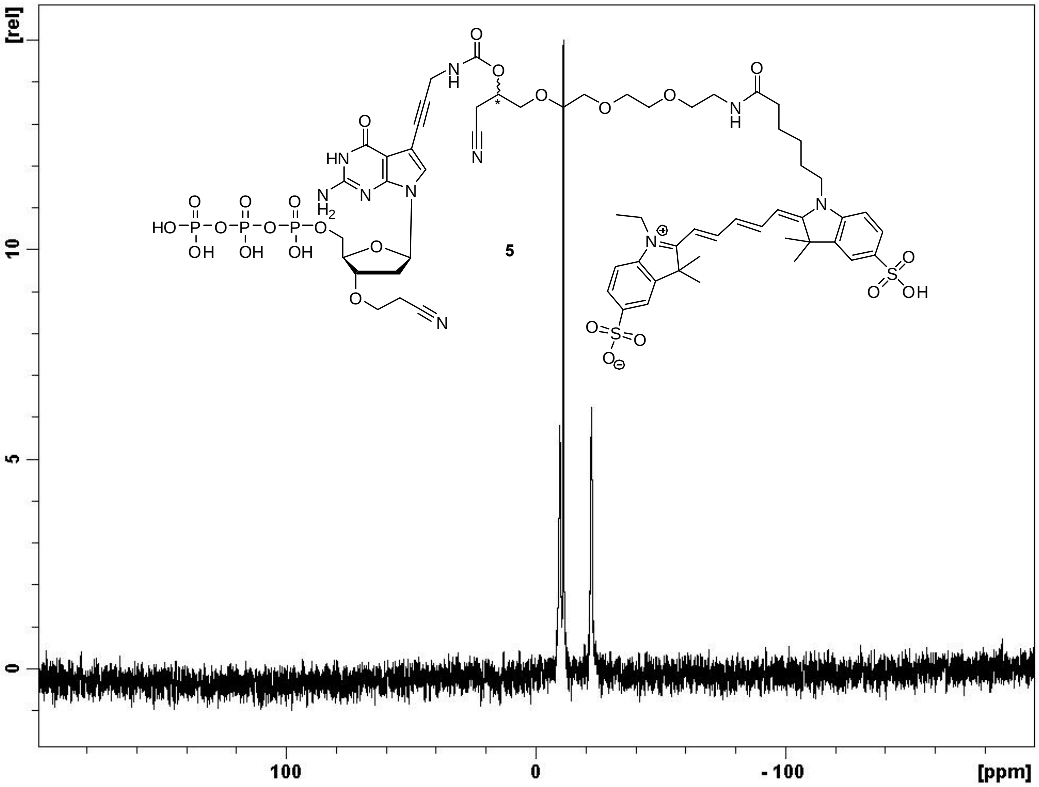

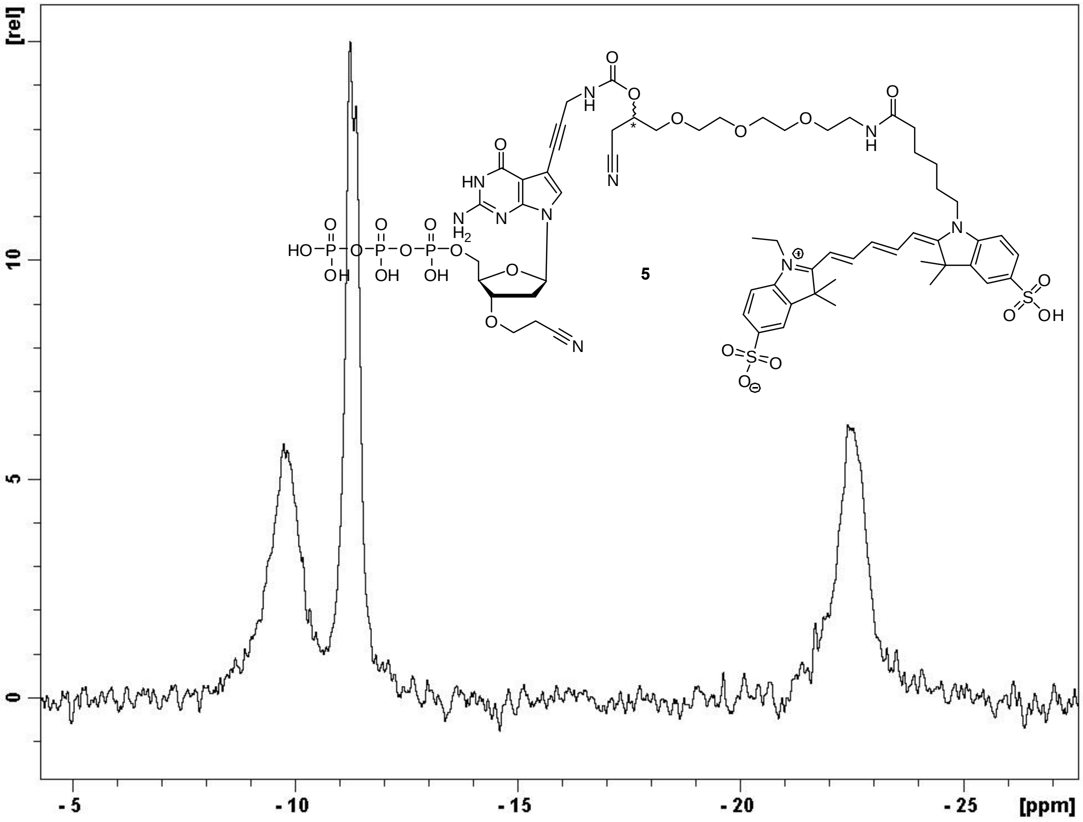

[rel]
10
5
0
- 5
- 10
- 15
- 20
- 25
[ppm]
5

ESI-MS spectrum:

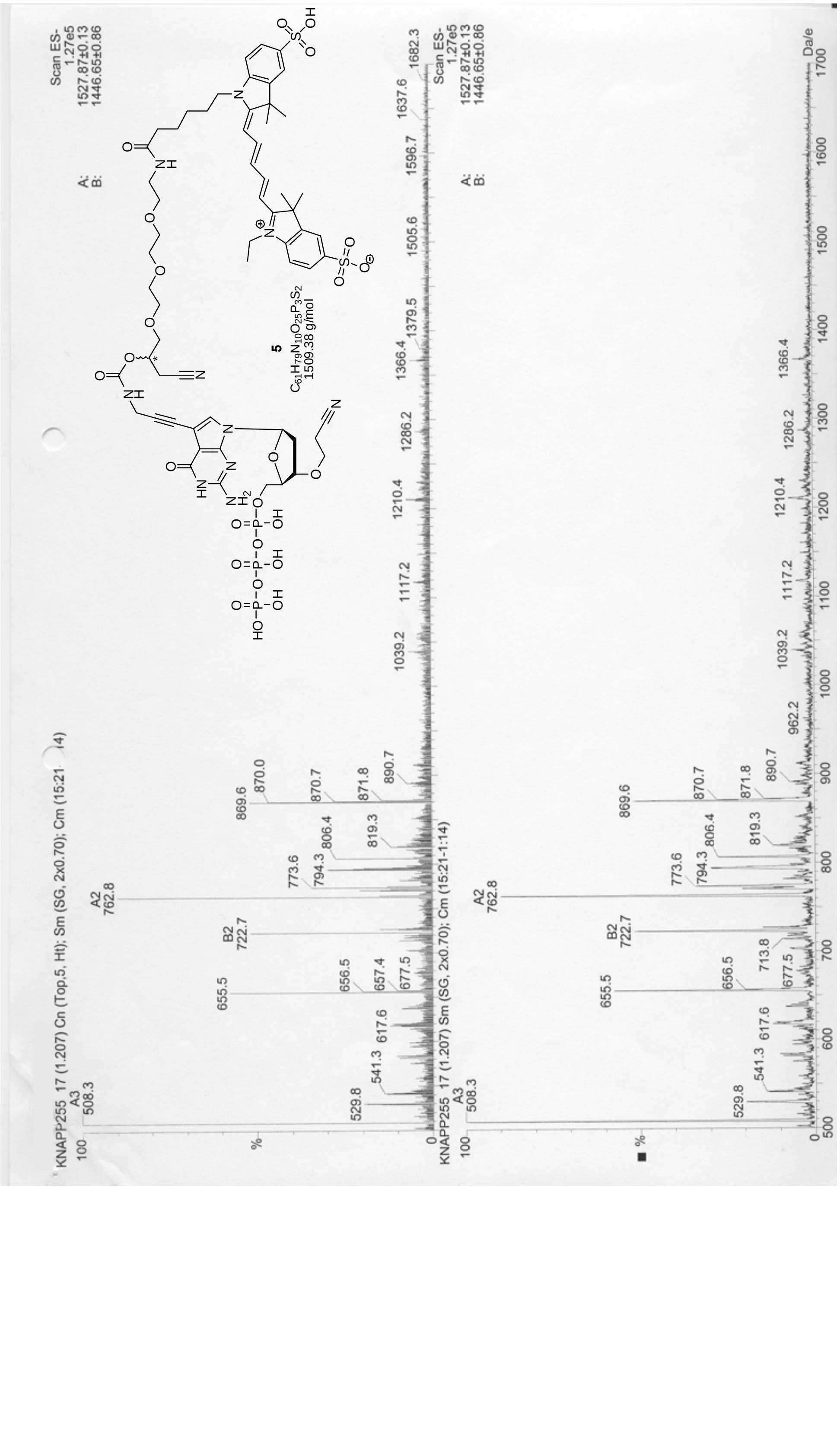

Supplement: Supplementary file 1 [file chem0017-2903-SD1.pdf]
